# Coordinated DNA 5-mC and RNA m^5^C methylation epigenetically regulates MZF1 splice variants to drive EGFR-TKI resistance

**DOI:** 10.1038/s12276-026-01758-4

**Published:** 2026-07-01

**Authors:** Huan Zhang, Yutong Pang, Bin Liu, Dongfeng Wang, Niying Zhao, Xiaojue Wang, PanJian Wei, Jie Lin, Shuye Lin

**Affiliations:** 1 Cancer Research Center, Beijing Chest Hospital, Capital Medical University, Beijing Tuberculosis and Thoracic Tumor Research Institute, Beijing, China; 2 Department of Gastroenterology, Beijing Chest Hospital, Capital Medical University, Beijing Tuberculosis and Thoracic Tumor Research Institute, Beijing, China; 3 Department of Thoracic Surgery, Dongying People’s Hospital, Dongying, Shandong China; 4 College of Computer Science and Cyber Security, Fujian Normal University, Fuzhou, Fujian China; 5 Laboratory for Clinical Medicine, Capital Medical University, Beijing, China

**Keywords:** Epigenetics, Pathogenesis

## Abstract

Acquired resistance to epidermal growth factor receptor-tyrosine kinase inhibitors (EGFR-TKIs) severely limits clinical efficacy. Myeloid zinc finger 1 (MZF1) inhibited EGFR phosphorylation and internalization by maintaining zinc homeostasis, which suggested its potential as a therapeutic target for resensitization. The two MZF1 splice variants exhibited functional divergence. MZF1^S^ demonstrated significantly reduced Zn^2+^-binding capacity compared with MZF1^L^ owing to C_2_H_2_ domain deletion. Although MZF1^S^ was specifically upregulated in resistant patients, MZF1^L^ predominated in treatment-sensitive cohorts, the mechanisms regulating their differential expression remained uncharacterized. We identified NSUN7/YBX1-mediated RNA m^5^C methylation as a critical driver of resistance. This modification promoted competitive binding of SRSF1 over SRSF3 to MZF1 pre-mRNA and enhanced MZF1^S^ production. Overlapping loci co-regulated by RNA m^5^C and DNA 5-methylcytosine methylation in the MZF1 5ʹ-untranslated region suggested coordinated epigenetic control of alternative splicing. Characterization of these loci revealed a UHRF1/DNMT1-NSUN7/YBX1 axis that synergistically regulated splice variant selection. Targeting this axis suppressed MZF1^S^ while restoring MZF1^L^ expression, which resensitized resistant cells to EGFR-TKIs. Our findings established a dual-layer epigenetic mechanism governing alternative splicing in drug resistance and proposed methylation-regulated splice variants as biomarkers and therapeutic targets for overcoming EGFR-TKI resistance in non-small-cell lung cancer.

## MZF1 splice variants drive resistance to EGFR-TKIs

Lung cancer, particularly non-small-cell lung cancer (NSCLC), remains a leading cause of cancer-related deaths globally. This study explores the role of epigenetic changes in acquired resistance to epidermal growth factor receptor tyrosine kinase inhibitors, a common treatment for NSCLC. Researchers identified that the zinc finger protein, myeloid zinc finger 1 (MZF1), with its two isoforms MZF1^L^ and MZF1^S^, has a crucial role in this resistance. MZF1^L^, which maintains zinc homeostasis, enhances drug sensitivity, whereas MZF1^S^ promotes resistance. The study used CRISPR/Cas9 technology and RNA methylation assays to demonstrate that NSUN7-mediated RNA m^5^C methylation and DNA methylation at the MZF1 5ʹ-untranslated region regulate these isoforms. These findings highlight MZF1 as a potential biomarker and therapeutic target for overcoming epidermal growth factor receptor-tyrosine kinase inhibitor resistance. Future research could focus on targeting these epigenetic modifications to improve treatment outcomes for patients with NSCLC.

This summary was initially drafted using artificial intelligence, then revised and fact-checked by the author.

## Introduction

Lung cancer remains one of the most prevalent malignancies worldwide, with non-small-cell lung cancer (NSCLC) constituting the predominant histological subtype^1^. The identification of epidermal growth factor receptor (EGFR) gene mutations as pivotal oncogenic drivers in NSCLC has ushered in a new era of biomarker-guided precision therapy for advanced-stage patients^2^. Successive generations of EGFR tyrosine kinase inhibitors (EGFR-TKIs), including first-generation gefitinib and third-generation osimertinib as representative agents, have been clinically developed. Nevertheless, acquired resistance persists as a critical limitation to the clinical efficacy of EGFR-TKIs. Emerging studies revealed that patients developing EGFR-TKI resistance exhibit substantial epigenetic alterations beyond the conventional understanding of genetic mutations^3,4^. Unlike irreversible genetic mutations, dynamic and reversible epigenetic modifications progressively modulate cellular differentiation trajectories and malignant transformation processes, offering novel perspectives for constructing resistance surveillance systems and identifying therapeutic sensitization targets^5,6^.

During acquired resistance development, EGFR undergoes ligand-independent phosphorylation, accompanied by disrupting Zn^2+^ homeostasis. This disruption was associated with enhanced EGFR phosphorylation while inducing its internalization, ultimately leading to marked reduction in drug sensitivity^7,8^. Our preliminary studies revealed that the expression of methylation-sensitive zinc finger protein myeloid zinc finger 1 (MZF1) is epigenetically regulated. Through Zn^2+^ binding that helped maintain zinc homeostasis and correlates with suppressed EGFR phosphorylation, MZF1 emerges as a potential epigenetic biomarker for monitoring acquired resistance to EGFR-TKIs and a therapeutic target for enhancing drug sensitivity. However, between the two isoforms (MZF1^L^ and MZF1^S^), MZF1^L^ encodes intact zinc finger domains whereas MZF1^S^ exhibits significantly diminished Zn^2+^ binding capacity owing to C_2_H_2_ domain deletion^9^. Clinical cohort analyses demonstrated predominant MZF1^S^ expression in resistant patients versus predominant MZF1^L^ expression in treatment-sensitive groups, although the precise regulatory mechanisms underlying their differential expression remain elusive. More importantly, we identified overlapping genomic loci in the MZF1 5’-untranslated region (UTR) containing both RNA m^5^C differential methylation sites and DNA CpGs. The frequent co-occurrence of these two epigenetic modifications at shared targets may establish a unique gene expression regulatory paradigm: integration of DNA 5-methylcytosine (5-mC)-mediated transcriptional effects with RNA m^5^C-dependent post-transcriptional regulation. Whether this combinatorial epigenetic influence correlates with alternative splicing mechanisms remains to be elucidated.

In this study, we reported differential expression of MZF1 transcripts that exhibited distinct functional and clinical implications in acquired EGFR-TKI resistance. NSUN7/YBX1-mediated RNA m^5^C methylation facilitated competitive binding of SRSF1 over SRSF3 to MZF1 pre-mRNA, which promoted MZF1^S^ expression and drove acquired resistance to EGFR-TKIs. Furthermore, we identified overlapping loci in the MZF1 5’-UTR where differentially methylated RNA m^5^C regions coincided with DNA CpG islands. We characterized the UHRF1/DNMT1-NSUN7/YBX1 regulatory axis that coordinated DNA 5-mC and RNA m^5^C methylation and inhibited the modification of the axis to promote MZF1^L^ expression and enhance cellular sensitivity to EGFR-TKIs. These findings elucidated the cooperative mechanism through which RNA m^5^C and DNA methylation synergistically regulated MZF1 alternative splicing, establishing the molecular foundation for clinical translation of MZF1 splice variants and methylation-regulated alternative splicing hot spots as resistance-monitoring biomarkers and anticancer therapeutic targets for sensitivity enhancement.

## Materials and methods

### Oligonucleotides, antibodies, and reagents

All oligonucleotide sequences and cDNA clones used to construct recombinant plasmids, antibodies, and the reagents used in this study are listed in Supplementary Tables 1–3, respectively.

### Cells

The human NSCLC cell lines HCC827 (CRL-2868) and PC9 (1101HUM-PUMC001694) were purchased from American Type Culture Collection (ATCC, USA) and National Biomedical Experiment Cell Resource Bank (BMCR, China). All cell lines were authenticated by the short tandem repeat method. *TransSafe*^*TM*^ Mycoplasma Prevention Reagent (cat. no. FM501-01, TransGene) was used to prevent mycoplasma contamination. Gefitinib-resistant (GR) and osimertinib-resistant (OR) cell lines were established by progressively exposing HCC827 and PC9 cells to escalating concentrations of gefitinib (5 nM–1 μM) or osimertinib (1–500 nM), respectively, following established protocols^10^. The cells were grown in 90% RPMI 1640 (cat. no. A1049101, Gibco) with 10% fetal bovine serum (cat. no. 10099141, Gibco).

### Construction

Genomic deletion of the MZF1 transcripts (MZF1-KO) was using a CRISPR-Cas9 bivector lentivirus that was custom-ordered from GeneChem (Shanghai). The single guide RNA (sgRNA) was AGGGCTCCATCTTCTCTGAT. Targeted methylation editing of MZF1 was achieved using a dual-vector CRISPR-Cas9 lentiviral system and the NSUN7, YBX1, and UHRF1 knockdown (shRNA) and overexpression plasmids were custom-engineered by GeneChem (Shanghai). Stable cell pools were selected through puromycin (cat. no. A3740, ApexBio Technology) treatment at 2 μg/ml for 14 days. For isoform-specific overexpression, HCC827 and PC9 cells were transfected with MZF1^L^ or MZF1^S^ expression constructs followed by G418 selection (0.5 mg/ml, cat. no. 11811031, Thermo Fisher Scientific) over a 2-week period. All transfections were performed using Lipofectamine 3000 (cat. no. L3000015, Thermo Fisher Scientific), following manufacturer’s standardized protocol.

### Patients and specimens

This study was conducted with ethical approval from the Institutional Review Board (protocols JYS-2021-013 and JYS-2021-033), and written informed consent was obtained from all participants. Three independent clinical cohorts were analyzed. The first cohort included 57 subjects comprising chronic pneumonia (CP, *n* = 10), patients before treatment (BT, *n* = 22), GR (*n* = 13) and OR (*n* = 12) (Supplementary Table 5). The second cohort included 179 patients stratified into chronic pneumonia or paracancerous tissue (CP, *n* = 61), patients BT (*n* = 47), GR (*n* = 38) and OR (*n* = 33) (Supplementary Table 6). Cohort 3 consisted of 60 patients, including CP (*n* = 10), patients BT (*n* = 27), GR (*n* = 15), and OR (*n* = 8) (Supplementary Table 11). Bronchoalveolar lavage fluid (BALF) specimens and in situ tissue samples were obtained through bronchoscopy or radical resection, respectively. BALF specimens were collected using the QIAsymphony PAXgene Blood ccfDNA Kit (cat. no. 768536, QIAGEN). The tissue samples were snap-frozen in liquid nitrogen in preparation for RNA extraction. Clinicopathological parameters were systematically extracted from institutional medical records, with NSCLC staging rigorously classified according to the American Joint Committee on Cancer 8th Edition guidelines. All experimental protocols adhered to standardized biospecimen handling and data annotation procedures.

### Western blotting

Protein extraction was performed using RIPA lysis buffer (cat. no. P0013B, Beyotime Biotech, China). Protein quantification was conducted with the Compat-Able™ BCA Assay Kit (cat. no. 23229, Thermo Scientific), following manufacturer's specifications. Protein samples were separated via SDS–PAGE (12% resolving gel) and electrophoretically transferred to polyvinylidene fluoride membranes using a Bio-Rad Mini PROTEAN 3 system. Membranes were blocked with 5% non-fat dry milk in PBS containing 0.1% Tween-20 (PBST) for 1 h at ambient temperature. Subsequent immunoblotting involved overnight incubation with primary antibodies at 4 °C, followed by 1 h room-temperature incubation with species-matched horseradish peroxidase-conjugated secondary antibodies (1:5,000 dilution). Protein bands were detected using the Amersham ECL Western Blotting Detection System under non-saturating exposure conditions. β-Actin immunoblotting served as internal loading control for normalization. All washing steps used PBST buffer with three 10-min cycles between antibody incubations.

### Immunofluorescence (IF), immunohistochemistry (IHC), and multiplexed immunohistochemistry (mIHC)

IF and IHC were performed as described previously^11^. For IF analysis, cells were fixed in 4% paraformaldehyde for 10 min at ambient temperature, permeabilized with 0.1% Triton X-100 in PBS, and blocked with 5% bovine serum albumin. The cells were incubated overnight at 4 °C with the primary antibodies followed by incubation with CoraLite 488/549-conjugated secondary antibodies for 1 h. 4',6-Diamidino-2-phenylindole was used for nuclear staining (10 μg/ml in PBS) (Invitrogen and Life Technologies). Images were then recorded by laser confocal microscopy (Olympus FV1000 Laser Scanning Confocal Microscope).

For conventional IHC, formalin-fixed paraffin-embedded tumor specimens were sectioned at 5 μm slices. Slices were incubated with the primary antibodies overnight at 4 °C. Dako REAL EnVision Detection System, peroxidase/diaminobenzidine (cat. no. K500711, DAKO), was used to visualize staining in accordance with the manufacturer’s instructions. Sections were counterstained with hematoxylin and eosin.

mIHC analysis of the tissue array was performed using a 5-color fluorescent IHC kit (cat. no. 10002100100; Panovue), according to manufacturer’s instructions. The tissue array slides were first dewaxed and subjected to heat-induced antigen retrieval at 95 °C for 20 min and then incubated overnight with primary antibodies. The antibodies were stripped by microwave treatment before the next primary antibody was added. Finally, the slides were mounted after counterstaining with 4',6-diamidino-2-phenylindole for 5 min. After washing, the slides were incubated at room temperature for 1 h with antimouse or antirabbit secondary antibodies labeled with horseradish peroxidase. For opal signal generation, one of the following fluorophore tyramines was added to the slides to detect antibody staining: Opal Polaris 480, 520, and 620 for MZF1^L^, MZF1^S^, and Pan-CK (dilution 1:2 to 1:1,000, Supplementary Table 2). Images of the multiple-color opal slides were obtained using the TissueFAXS Spectra S system (TissueGnostics), and quantitative analysis of protein expression was performed using an automated quantitative analysis system (TissueGnostics, StrataQuest_7.0.1.165).

### Cell viability

The viability of HCC827 and PC9 cells seeded into 96-well plates at 2 × 10^3^ cells/well was measured every day using a 3-(4,5-dimethylthiazol-2-yl)-2,5-diphenyltetrazolium bromide assay kit (cat. no. ab211091, Abcam). A microplate reader was used to detect the absorbance at 490 nm, as described previously^11,12^.

### Colony formation

The cells were seeded in 6-well tissue culture plates (0.3 × 10^3^ cells/well) in triplicate. Colonies containing more than 50 cells were counted after 1 week. The cells were fixed with 75% ethanol for 30 min and stained with 0.2% crystal violet (cat. no. C0121, Beyotime Biotech) for 20 min following a previous protocol^11,12^.

### Cell migration

Membranes with 8-μm pores (cat. no. 3115, Corning) were inserted into a Transwell apparatus. The upper chamber of the apparatus was coated with Matrigel and seeded with 2 × 10^4^ cells, which were incubated for 24 h (refs. ^11,12^).

### RNA isolation and RT–qPCR

RNA was isolated using TRIzol Reagent (cat. no. 15596018, Invitrogen), and first-strand cDNA was synthesized with the Superscript First-Strand Synthesis System (cat. no. 11904018, Invitrogen). mRNA expression levels were analyzed using 2× SYBR Green-based quantitative PCR (qPCR) reagent on an ABI 7500 qPCR machine (Applied Biosystems). The relative expression level of each gene was normalized to that of the same cDNA using the ${2}^{-\mathrm{\varDelta \varDelta }{C}_{t}}$ method and compared with its own control.

### DNA analysis, quantitative PCR (MSP-qPCR), and MassARRAY

Genomic DNA was extracted using the QIAamp DNA Mini Kit (cat. no. 51304, QIAGEN). DNA was extracted from the BALF and tissue samples using the QIAsymphony PAXgene Blood ccfDNA Kit (cat. no. 768536, Qiagen), following the manufacturer’s standard protocol. A NanoDrop 1000 spectrophotometer (Thermo Fisher Scientific) was used for DNA quantification. DNA was bisulfite-modified using the Zymo DNA Methylation Kit (cat. no. D5006, Zymo Research) and further analyzed by qPCR using methylated or unmethylated primer pairs. The relative levels of methylated and unmethylated MZF1 determined by qPCR were normalized to β-actin using the ${2}^{-\mathrm{\varDelta \varDelta }{C}_{t}}$ method^11^. The methylation of MZF1 at the CpG site was also detected using the MassARRAY Compact system (Sequenom) provided by Oebiotech Company in Beijing. MassARRAY EpiTYPER (Sequenom) was used for data analysis to generate quantitative results for each CpG site or an aggregate of the results for multiple CpG sites.

### Methylated DNA immunoprecipitation (MeDIP) and crosslinking immunoprecipitation (CLIP)

For MeDIP, genomic DNA was extracted from lung cancer cells. After sonication, the fragmented DNA was immunoprecipitated with a monoclonal antibody against 5-methylcytidine or nonspecific IgG at 4 °C overnight. For CLIP, cells were supplemented with 4-thiouridine for 14 h, followed by ultraviolet light crosslinking. Ten percent of the sonicated DNA was saved as an input control. Immunoprecipitated DNA fragments were released by proteinase K digestion and then purified using a QIAquick purification kit (cat. no. 28104, Qiagen). The enrichment of the precipitated DNA was determined by qPCR and is represented as “% of input” after normalization to the genomic DNA pulled down by nonspecific IgG.

### Methylated RNA immunoprecipitation (MeRIP) and RNA immunoprecipitation (RIP)

For MeRIP, RNA was extracted from lung cancer cells. The fragmented RNA was immunoprecipitated with a monoclonal antibody against 5-methylcytidine or nonspecific IgG at 4 °C overnight. Ten percent of RNA was saved as an input control. Immunoprecipitated RNA fragments were purified using RNAeasy Animal RNA Isolation Kit (cat. no. R0024, Beyotime Biotech). The enrichment of the precipitated RNA was determined by qPCR.

RNA was precleared with protein A/G beads. Aliquots of a precleared solution, named IP fractions, were incubated with 2 μg of antibody against NSUN7 or YBX1 or preimmune rabbit IgG on a rotating platform at 4 °C overnight. About 1% of the IP fraction served as the RIP input control. The antibody-enriched protein–RNA complexes were precipitated with protein A/G beads from the IP fractions. Immunoprecipitated RNA fragments were purified using RNAeasy Animal RNA Isolation Kit (cat. no. R0024, Beyotime Biotech). The immunoprecipitated RNA fractions were analyzed by qPCR.

### RNA-Bis-Seq

RNA-bisulfite-sequencing (RNA-BiS) libraries were constructed by Seqhealth Technology Co., Ltd (Wuhan, China). Total RNA was treated with DNase I (37 °C, 30 min) to remove genomic DNA contamination. Poly(A)+ mRNA enrichment was performed using the Dynabeads™ mRNA Purification Kit (Thermo Fisher Scientific), following manufacturer’s protocol. Bisulfite conversion of purified RNA was conducted with the EZ RNA Methylation Kit (cat. no. 5006, Zymo Research) under optimized thermal conditions (70 °C for 5 min and 54 °C for 45 min), followed by purification via Zymo-Spin IC Columns. Reverse transcription of bisulfite-converted RNA was performed using ACT random hexamers and Superscript II Reverse Transcriptase (Invitrogen). The synthesized cDNA was processed for library preparation, with quality control performed using an Agilent 2100 Bioanalyzer and qPCR. Final libraries were sequenced on the Illumina NovaSeq 6000 platform (paired-end, 150 bp reads).

### Co-immunoprecipitation (co-IP)

The protein was prepared using RIPA Lysis Buffer (cat. no. P0013B, Beyotime Biotech). Protein was precleared with protein A beads, and then protein was measured using a BCA Protein Assay Kit (cat. no. 23229, Thermo Scientific). Protein was incubated with 5 μg of specific anti-NSUN7, anti-YBX1 or pre-immune rabbit IgG on a rotation platform at 4 °C overnight. The antibody-enriched protein–protein complexes were precipitated with protein A beads from the IP fractions. A final PBS wash was followed by the addition of 60 μl of 2× loading buffer and 5 min incubation at 100 °C. Immunoprecipitated proteins were analyzed by western blotting.

### Intracellular Zn^2+^ analysis

Intracellular zinc levels were measured using Zinc Assay Kit (Beyotime Biotech, S2201), according to manufacturer’s instructions. For cultured cells, pellets were lysed in BeyoLysis Buffer A for Metabolic Assay (100 μl per 1 × 10^6^ cells) and homogenized on ice. Tissue samples were homogenized in the same buffer (100 μl per 10 mg tissue). All samples were deproteinized by adding an equal volume of deproteinization solution, incubated at room temperature for 5 min, and centrifuged at 14,000×*g* for 10 min at 4 °C. Supernatants were collected and 50 μl aliquots were mixed with 200 μl of working solution (zinc probe: zinc reagent buffer = 4:1) in 96-well plates. After 10 min of incubation at room temperature, absorbance was measured at 560 nm. Zinc concentrations were calculated against a standard curve.

### Tumorigenicity

The animal handling protocols and all in vivo experimental procedures were approved by the Institutional Animal Ethics Committee of the Beijing Chest Hospital. HCC827, HCC827/GR, and HCC827/OR cells (4 × 10^6^) transfected with MZF1^L^, MZF1^S^, or control vector (Vector) in 0.1 ml of PBS were subcutaneously injected into both flanks of 4-week-old BALB/C female athymic mice (Vital River Laboratories, Beijing, China). After 8 days of injection, mice were treated with gefitinib, osimertinib, 5-Aza-2ʹ-deoxycytidine (5-Aza-CdR), and DATS. The tumor diameters and body weights of the NSCLC xenograft tumor-bearing nude mice were measured and documented every 8 days until the animals were sacrificed on day 40. NSCLC tumor xenografts were isolated and weighed. Tumor volume was calculated by measuring the longest (*a*) and shortest (*b*) dimensions of the tumor and calculated by the formula *ab*2/2.

### Culture of tumor organoids

The tissues of appropriate dimensions underwent mincing, washing, and subsequent incubation at 37 °C with Tumor Tissue Digestion Solution (K601003, BioGenous Technologies, Suzhou, China) on an orbital shaker for 1–2 h. The resultant suspension was filtered through a 100-μm nylon cell strainer to remove tissue fragments. After centrifugation and resuspension, cells were seeded onto Matrigel Organoid Culture ECM (M315066, BioGenous Technologies) within 24-well plates and overlaid. The culture medium was replenished every 4 days, and organoids were passaged every 1–4 weeks.

### Statistical analyses

The data are expressed as mean ± standard deviation (SD) of at least three experiments. The expression of the MZF1 transcripts and their epigenetic alterations was analyzed using the two‑tailed Student’s *t* test or one‑way analysis of variance with Tukey’s post hoc test. The relationships among the expression of MZF1^L^, MZF1^S^, and clinicopathologic characteristics were measured by χ^2^ exact tests. The Spearman rank test and Fisher’s exact test were used to analyze clinicopathological correlations. All statistical analyses were performed using SPSS (RRID:SCR_002865) version 23.0 (IBM Corp.) and R package (R Studio).

## Result

### MZF1 splicing variants were divergently expressed in EGFR-TKI-resistant cell lines and tissues

**Fig. 1:**
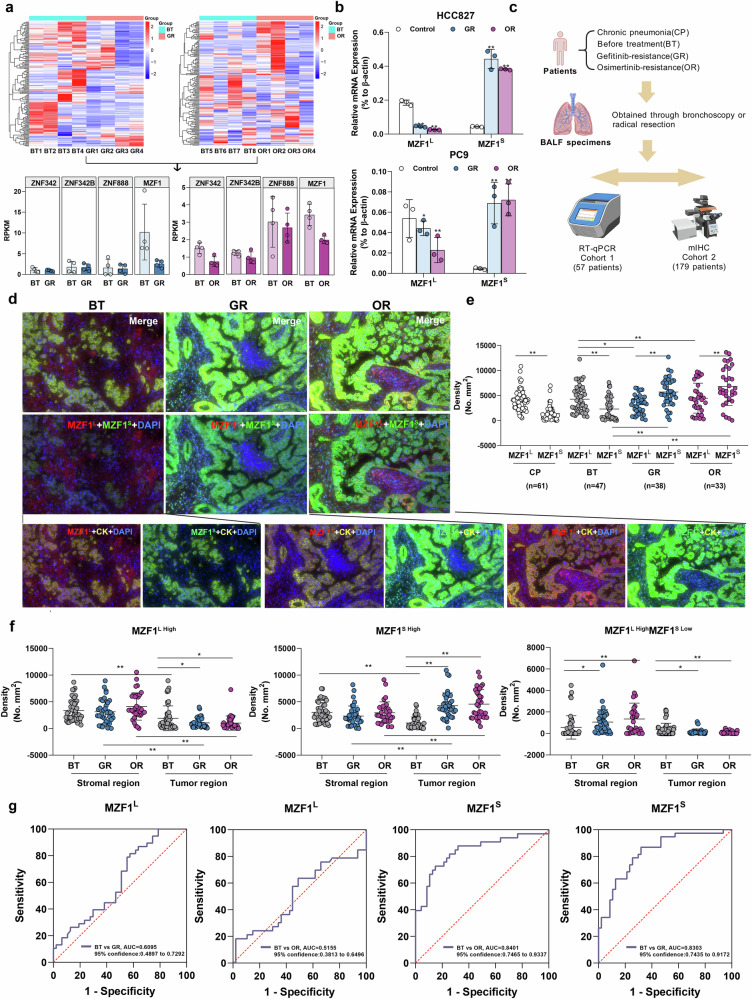
Differential expression of MZF1 transcripts in gefitinib-resistant/osimertinib-resistant cells and tissues. **a** Heatmap visualizing *Z*-scores of transcriptome data. **b** Reverse transcription (RT)–quantitative PCR (qPCR) analysis of the mRNA expression of MZF1^L^ and MZF1^S^ in GR/OR HCC827 and PC9 cells. **c** Schematic diagram of the clinical cohorts. **d** Multiplexed immunohistochemistry (mIHC) determination of the MZF1 isoforms in a commercial tissue array. MZF1 isoforms stained with antibodies specifically against MZF1^L^ (pseudocolored red), MZF1^S^ (pseudocolored green), and pan-CK (pseudocolored yellow) were used to stain and identify bound tumor cells (pseudocolored yellow). 4',6-Diamidino-2-phenylindole (DAPI)-stained nuclei are shown in blue. The images are shown at original magnifications of ×400. **e** Quantitative analysis of the protein levels of the MZF1 isoforms in the tissue array specimens. **f** Quantitative analysis of the protein levels of the MZF1 isoforms in the tissue array specimens from stromal region and tumor region. **g** The receiver operating characteristic curve compared between the patient BT group and the drug-resistant patient group. *P*-values were calculated by one‑way analysis of variance with Tukey’s post hoc test (panels **b**, **e**, and **f**). AUC, area under the curve; BALF, bronchoalveolar lavage fluid; MZF1, myeloid zinc finger 1. *P < 0.05 and **P < 0.01, significant differences from the control.

To investigate the potential role of Zn^2+^ concentration regulators in EGFR-TKI resistance, we collected four specimens each from patients treated with gefitinib and osimertinib before and after drug resistance development, followed by transcriptome sequencing to explore expression changes in zinc finger protein family members. ZNF342, ZNF342B, ZNF888, and MZF1 showed consistent trends in both the GR and OR groups, with MZF1 exhibiting the most significant change among these four genes (Fig. 1a). Considering that different transcript variants of MZF1 exhibit distinct Zn^2+^ transport capacities, we analyzed the expression of MZF1 transcripts (MZF1^L^ and MZF1^S^) in EGFR-TKI-resistant cell lines. Reverse transcription (RT)–qPCR with transcript-specific primers (Supplementary Fig. 1a) revealed differential MZF1 expression between EGFR-TKI-sensitive and EGFR-TKI-resistant cell lines (Fig. 1b). MZF1^L^ was predominantly expressed in sensitive cells, whereas MZF1^S^ was predominantly expressed in EGFR-TKI-resistant (GR/OR) cell lines. Clinical validation across two independent cohorts demonstrated this expression (Fig. 1c). In cohort 1 (BALF specimens, *n* = 57), MZF1^L^ levels were significantly reduced in GR (*n* = 13) and OR (*n* = 12) patients compared with patients BT (*n* = 22), whereas MZF1^S^ showed inverse correlation (Supplementary Tables 4 and 5 and Supplementary Fig. 1b). Progressive MZF1^L^ downregulation paralleled advancing tumor malignancy (Supplementary Fig. 1c), confirming transcript-specific dysregulation in EGFR-TKI resistance.

To resolve isoform-specific protein expression, we developed custom antibodies targeting unique MZF1^L^/MZF1^S^ epitopes (Supplementary Fig. 1a). Western blotting confirmed MZF1^L^ restriction to sensitive cells, whereas MZF1^S^ dominated resistant lines (Supplementary Fig. 1d). Immunofluorescence revealed distinct subcellular partitioning: MZF1^L^ exhibited cytoplasmic/nuclear localization versus nuclear-enriched MZF1^S^ (Supplementary Fig. 1e). mIHC analysis of cohort 2 (*n* = 179 tissues) demonstrated spatial segregation, and MZF1^L^ localized to CK-positive (CK^+^) tissue area, contrasting to MZF1^S^ accumulation in CK-negative (CK^−^) tissue area (Table 1, Supplementary Table 6, Fig. 1d, and Supplementary Fig. 1f). Quantitative analysis revealed high expressed MZF1^S^ (MZF1^S High^) but low expressed MZF1^L^ (MZF1^L Low^) in EGFR-TKI-resistant tissues compared with tissues BT (Fig. 1e). This is consistent with MZF1^S High^ in tumor area and MZF1^L High^ in stromal tumor area (Fig. 1f). Receiver operating characteristic curve analysis demonstrated that elevated expression of MZF1^S^ may serve as a predictive factor for drug resistance (Fig. 1g). These results suggested that the divergent expression of the MZF1 transcripts MZF1^S High^ MZF1^L Low^ in EGFR-TKI-resistant tissues compared with MZF1^S Low^ MZF1^L High^ in BT is distinct not only at the mRNA level but also at the protein level. These findings establish coordinated mRNA–protein level dysregulation of MZF1 isoforms during resistance development.

**Table 1:** Association of myeloid zinc finger 1 (MZF1) expression with clinicopathologic parameters in non-small-cell lung cancer (tissues, *n* = 179).

Characteristics	Cases	MZF1^L^ expression			MZF1^S^ expression		
		High (%)	Low (%)	*P*-value	High (%)	Low (%)	*P*-value
**Total**	179	93 (51.7)	86 (48.3)		76 (42.2)	103 (57.8)	
**Gender**							
Male	101	55 (54.5)	46 (45.5)		42 (41.6)	59 (58.4)	
Female	78	38 (48.7)	40 (51.3)	0.4462	34 (43.6)	44 (56.4)	0.7878
**Age**							
≥60	116	65 (56.0)	51 (44.0)		54 (46.6)	62 (53.4)	
<60	63	28 (44.4)	35 (55.6)	0.1383	22 (34.9)	41 (65.1)	0.1327
**Pathological type**							
Pneumonia/paracancerous	61	33 (54.1)	28 (45.9)		5 (8.2)	56 (91.8)	
Adenocarcinoma	72	36 (50.0)	36 (50.0)	0.6374	42 (58.3)	30 (41.7)	**<0.01**
Squamous cell carcinomas	46	24 (52.2)	22 (47.8)	0.8434	29 (63.0)	17 (37.0)	**<0.01**
**Tumor invasive depth**							
1	41	18 (43.9)	23 (56.1)		15 (36.6)	26 (63.4)	
2	25	12 (48.0)	13 (52.0)	0.7457	18 (72.0)	7 (28.0)	**0.0053**
3	21	10 (47.6)	11 (52.4)	0.7808	13 (61.9)	8 (38.1)	0.0579
4	28	17 (60.7)	11 (39.3)	0.1702	23 (82.1)	5 (17.9)	**0.0002**
NA	65						
**Lymph node metastasis**							
0	52	24 (46.2)	28 (53.8)		27 (51.9)	25 (48.1)	
1	11	5 (45.5)	6 (54.5)	0.9663	5 (45.5)	6 (54.5)	0.6966
2	31	17 (54.8)	14 (45.2)	0.4440	22 (80.0)	9 (20.0)	0.0879
3	21	11 (52.4)	10 (47.6)	0.6297	15 (71.4)	6 (28.6)	0.1270
NA	65						
**Distant metastasis**							
Yes	80	38 (47.5)	42 (52.5)		42 (52.5)	38 (47.5)	
No	35	19 (54.3)	16 (45.7)	0.5031	27 (77.1)	8 (22.9)	**0.0131**
NA	65						
**Clinical stage**							
I	39	18 (46.2)	21 (53.8)		15 (38.5)	24 (61.5)	
II	6	3 (50.0)	3 (50.0)	0.8605	4 (66.7)	2 (33.3)	0.1929
III	33	16 (48.5)	17 (51.5)	0.8435	27 (81.8)	10 (18.2)	**0.0025**
IV	37	20 (54.1)	17 (45.9)	0.4912	27 (73.0)	10 (27.0)	**0.0025**
NA	65						
**EGFR resistance**							
No	47	23 (48.9)	24 (51.1)		14 (29.8)	33 (70.2)	
GR	38	17 (44.7)	21 (55.3)	0.6998	30 (78.9)	8 (21.1)	**<0.01**
OR	33	20 (60.6)	13 (39.4)	0.3027	27 (81.8)	6 (18.2)	**<0.01**
NA	61						

*EGFR* epidermal growth factor receptor, *GR* gefitinib-resistant, *NA* not available, *OR* osimertinib-resistant. Bold values indicated statistically significant differences at *P* < 0.05.

### MZF1 transcripts display dual functions associated with clinical relevance in patients with EGFR-TKI resistance

**Fig. 2:**
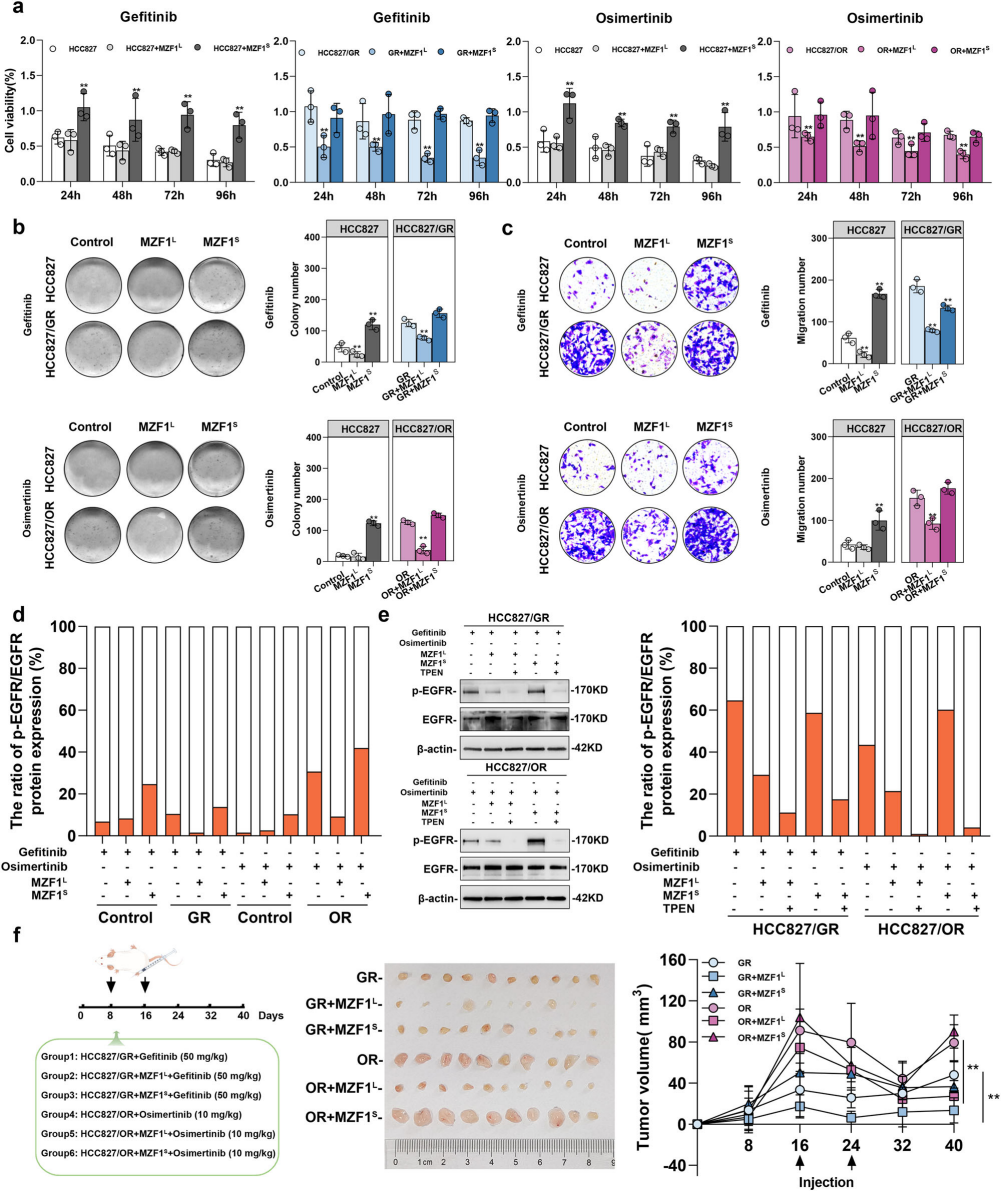
Dual effects of MZF1 transcripts on the malignancy and EGFR-TKI sensitivity of lung cancer cells. **a–c** Overexpression of myeloid zinc finger 1 (MZF1)^L^ and MZF1^S^ had divergent effects on cell proliferation and metastasis in HCC827-sensitive or HCC827-resistant cells with gefitinib (1 μM)/osimertinib (0.1 μM) treatment, as measured by 3-(4,5-dimethylthiazol-2-yl)-2,5-diphenyltetrazolium bromide (part **a**), colony formation (part **b**), and Transwell assays (part **c**), with representative images in the left panels and quantification data presented in the right panels. **d** Western blotting analysis of epidermal growth factor receptor (EGFR) and p-EGFR in HCC827, HCC827/GR, and HCC827/OR cells; cells were treated with gefitinib and osimertinib and/or transfected with MZF1^L^ and MZF1^S^. The quantification protein levels of p-EGFR/EGFR ratio were shown. **e** Western blotting analysis of EGFR and p-EGFR in HCC827, HCC827/GR, and HCC827/OR cells; cells were treated with gefitinib, osimertinib, and TPEN and/or transfected with MZF1^L^ and MZF1^S^. Left panel: representative images. Right panel: quantification protein levels of p-EGFR/EGFR ratio. **f** The effect of MZF1 transcripts on lung tumorigenicity with gefitinib (50 mg/kg)/osimertinib (10 mg/kg) treatment. HCC827/GR or HCC827/OR cells with ectopic expression of MZF1^L^ or MZF1^S^ were transplanted subcutaneously into the left and right flanks of nude mice (*n* = 2 flanks × 5 mice in each group). Left panel: images of dissected xenograft tumors from nude mice. Right panel: tumor volumes of tumor-bearing mice measured at the indicated time points. Data are presented as mean ± standard deviation (*n* = 3). *P*-values were calculated by one‑way analysis of variance with Tukey’s post hoc test (parts **a**–**c**) and two-way analysis of variance with repeated measures (part **f**). *P < 0.05 and **P < 0.01, significant differences from the control. GR, gefitinib-resistant; OR, osimertinib-resistant.

Stable MZF1^L^/MZF1^S^-overexpressing models were established in HCC827 and PC9 cells across EGFR-TKI-sensitive and EGFR-TKI-resistant contexts (Supplementary Fig. 2a). Functional characterization revealed isoform-specific oncogenic regulation: MZF1^S^ exhibited tumor-promoting activity, whereas MZF1^L^ demonstrated tumor-suppressive effects (Supplementary Fig. 2b–d). Next, we measured the functional roles of MZF1 transcripts with gefitinib/osimertinib treatment. MZF1^L^ overexpression significantly inhibited proliferation and migration in GR/OR cells, whereas MZF1^S^ overexpression exacerbated drug-resistant phenotypes by enhancing proliferative and migratory capacities (Fig. 2a–c). To investigate MZF1 transcript functionality in acquired EGFR-TKI resistance, we analyzed EGFR signaling in EGFR-TKI-treated GR/OR cells. Despite gefitinib/osimertinib exposure, HCC827/GR and HCC827/OR maintained elevated p-EGFR levels. Remarkably, MZF1^L^ overexpression significantly reduced the p-EGFR/EGFR ratio, demonstrating restored EGFR-TKI efficacy, whereas MZF1^S^ overexpression showed the opposite effect (Fig. 2d and Supplementary Fig. 3a). Subcellular fractionation revealed that MZF1^L^ promoted membrane EGFR retention but significantly reduced membrane p-EGFR levels in resistant cells, accompanied by concurrent reductions in cytoplasmic EGFR and nuclear EGFR (Supplementary Fig. 3b). These findings demonstrated MZF1^L^-mediated membrane retention of EGFR-enhanced EGFR-TKI sensitivity. Intracellular Zn^2+^ analysis and TPEN (Zn^2+^ chelator) rescue experiments demonstrated that MZF1^L^ binds intracellular Zn^2+^ to reduce free Zn^2+^ concentration, which correlated with decreased EGFR phosphorylation and ultimately enhanced sensitivity to EGFR-TKIs (Fig. 2e and Supplementary Fig. 3c). Subcutaneous xenograft models were established by injecting MZF1^L^/MZF1^S^-overexpressing HCC827/OR and HCC827/GR cells into nude mice with EGFR-TKI treatment. MZF1^L^ overexpression significantly restrained tumor progression in EGFR-TKI-treated groups, whereas MZF1^S^ overexpression accelerated tumor growth (Fig. 2f and Supplementary Fig. 4). These in vivo findings corroborated the isoform-specific therapeutic effects observed in vitro, demonstrating that MZF1^L^ enhanced but MZF1^S^ diminished EGFR-TKI responsiveness.

To explore the functional basis of the MZF1 transcripts, potential MZF1-targeted downstream genes were profiled using network analysis of a dataset available from The Cancer Genome Atlas (Supplementary Fig. 5a). Gain-of-function analyses of the six key hub genes in the network (LENG8, CSAD, MAMDC4, SFRS16, SNRP70, and ZNF446) showed that MZF1^L^ overexpression specifically upregulated CSAD, MAMDC4, and ZNF446, whereas MZF1^S^ overexpression selectively induced LENG8, SFRS16, and SNRP70 (Supplementary Fig. 5b). The Kaplan–Meier survival analysis showed a significantly worse overall survival for patients with lung cancer with LENG8^High^, SFRS16^High^, or SNRNP70^High^ but a favorable outcome with CSAD^High^, MAMDC4^High^, or ZNF446^High^ (Supplementary Fig. 5c), suggesting that the MZF1 transcripts confer diverse functional outcomes by targeting different molecular pathways.

### NSUN7 mediates RNA m^5^C methylation to promote MZF1 alternative splicing

**Fig. 3:**
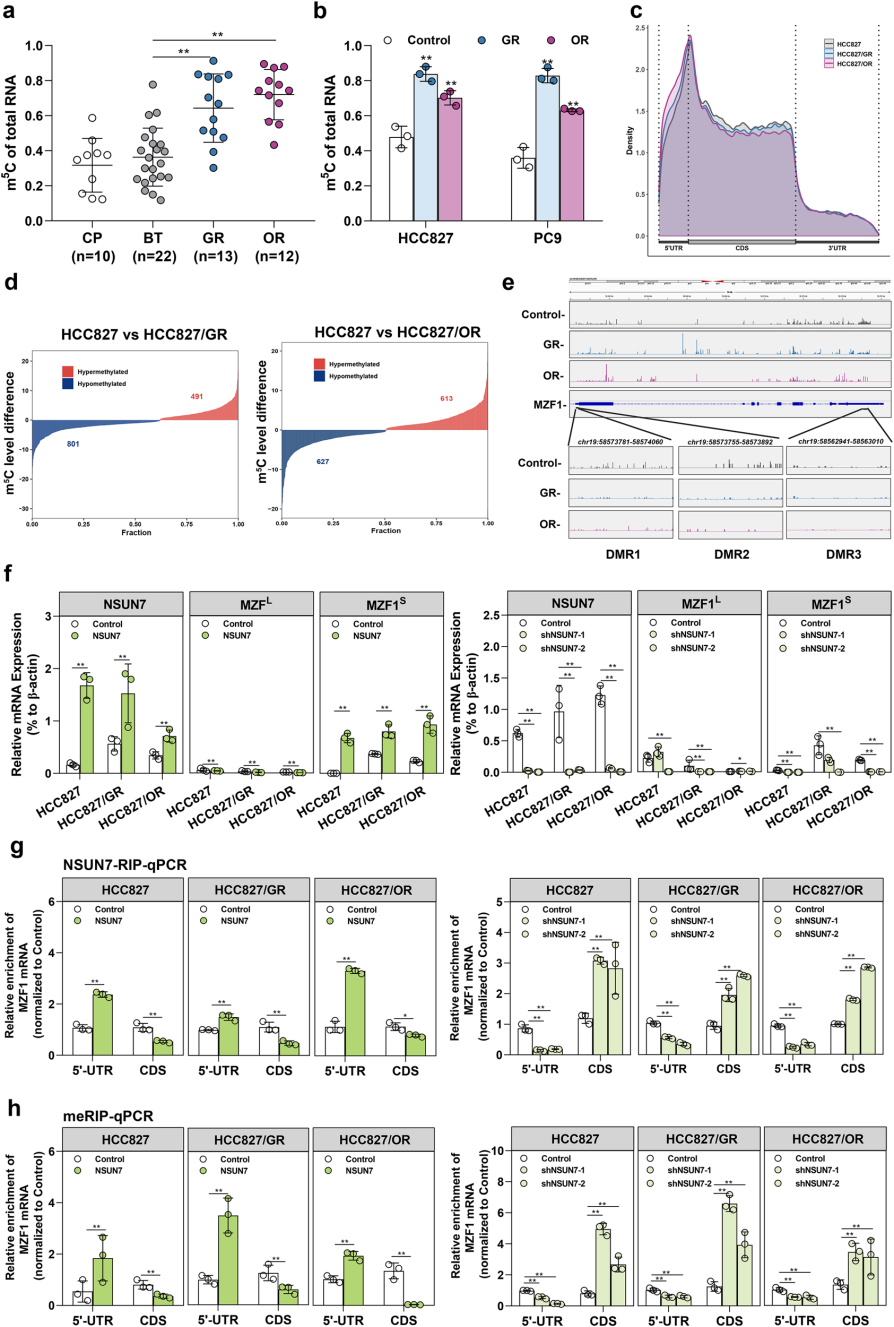
Effect of NSUN7-mediated RNA m^5^C methylation on expression of MZF1 splicing variants. **a** ELISA analysis of RNA m^5^C methylation in gefitinib-resistant/osimertinib-resistant patients. **b** ELISA analysis of RNA m^5^C methylation in gefitinib-resistant/osimertinib-resistant HCC827 and PC9 cells. **c** The m^5^C distributions within different regions in HCC827, HCC827/GR, and HCC827/OR cells. **d** Differential methylation levels of m^5^C sites between HCC827/GR versus HCC827 and HCC827/OR versus HCC827. **e** IGV analysis showed the changes in m^5^C methylation level of myeloid zinc finger 1 (MZF1) in DMR regions. **f** RT–qPCR analysis of the expression of NSUN7, MZF1^L^, and MZF1^S^ in NSUN7-overexpressed/knockdown GR and OR cells. **g** RIP-qPCR analysis of anti-NSUN7-enriched RNA fragments associated with MZF1 in HCC827, HCC827/GR, and HCC827/OR cells stably transfected with NSUN7 or NSUN7 knockdown. **h**, Methylated RNA immunoprecipitation (MeRIP)-qPCR analysis of anti-5-methylcytosine (5-mC)-enriched RNA fragments associated with MZF1 in HCC827, HCC827/GR, and HCC827/OR cells stably transfected with NSUN7 or NSUN7 knockdown. *P*-values were calculated by a *t* test (panels **f–h**) and one‑way analysis of variance with Tukey’s post hoc test (panels **a**, **b**, and **f**–**h**). *P < 0.05 and **P < 0.01, significant differences from the control. BT, before treatment; CP, chronic pneumonia; GR, gefitinib-resistant; OR, osimertinib-resistant; qPCR, quantitative PCR; UTR, untranslated region; IGV Integrative Genomics Viewer, CDS Coding sequence, DMR Differentially Methylated Region, RIP-qPCR RNA Immunoprecipitation combined with quantitative real-time PCR.

To investigate the regulatory mechanisms underlying the differential expression of MZF1^L^/MZF1^S^ in EGFR-TKI resistance in NSCLC, we first detected alterations in RNA m^5^C modification in both EGFR-TKI-resistant patients and cell lines. Our results demonstrated that EGFR-TKI resistance promoted a global increase in RNA m^5^C modification (Fig. 3a, b). Comparative RNA m^5^C methylation profiling in EGFR-TKI-sensitive (HCC827) versus EGFR-TKI-resistant (HCC827/GR and HCC827/OR) cells revealed region-specific epigenetic alterations, 5’-UTR hypermethylation, and CDS hypomethylation in resistant lines, with 3’-UTR remaining stable (Fig. 3c and Supplementary Fig. 6a). Differential methylation analysis identified 801 hypomethylated versus 491 hypermethylated sites in HCC827/GR, and 627 hypomethylated versus 613 hypermethylated sites in HCC827/OR relative to HCC827 cells (Fig. 3d and Supplementary Fig. 6b). Then, we examined the epigenetic contribution to the differential expression of the MZF1 splice variants, respectively. By analysis of the relative amount of RNA m^5^C methylation changes that occurred along the gene body (CDS) and flanking regions (5’-UTR and 3’-UTR) of MZF1, the data showed condensed increases in the methylation of exon and 3’-UTR in HCC827/GR and HCC827/OR cells, respectively, whereas the decreases were mainly underpinned in the 5.-UTR, including the promoter region. Further analysis shows that there are two DMRs (chr19:58573781–58574060 and chr19:58573755–58573892) in the 5ʹ-UTR of MZF1, an overlap was in the location of these two DMRs. In addition, there was a DMR in the CDS region of MZF1 (chr19:58562941–58563010) (Fig. 3e). Functional enrichment of hypermethylated m^5^C sites implicated mRNA splicing machinery, NSCLC pathways, and EGFR-TKI resistance mechanisms (Supplementary Fig. 6c,d and Supplementary Tables 7–10).

To clarify the epigenetic mechanism regulating the expression of MZF1 transcripts, NSUN1–7 members of the NOL1/NOP2/SUN (NSUN) protein family were examined. The results showed that NSUN2, NSUN6, and basally low-expressed NSUN7 were significantly upregulated in EGFR-TKI-resistant HCC827 and PC9 cells (Supplementary Fig. 7a). Consistently, we observed that NSUN7 expression was significantly elevated in the GR and OR groups within cohort 1 tissues (Supplementary Fig. 7b). Functional studies demonstrated NSUN7 specifically suppressed MZF1^L^ while promoting MZF1^S^ expression, whereas NSUN2/6 overexpression showed no regulatory effects. However, NSUN7 knockdown failed to reciprocally modulate MZF1^L^ levels, indicating complex regulatory dynamics requiring further elucidation (Fig. 3f and Supplementary Fig. 7c,d). NSUN7 gain-of-function exacerbated EGFR-TKI-resistant phenotypes (proliferation/migration) in both sensitive and resistant models, whereas loss-of-function attenuated these effects (Supplementary Fig. 7e,f). To investigate the correlation between NSUN7-mediated methylation and the differential expression of MZF1 splice variants, we first monitored the enrichment of NSUN7 at DMRs in the 5ʹ-UTR and CDS regions of MZF1 following NSUN7 overexpression. RIP/MeRIP-qPCR analyses revealed NSUN7-mediated m^5^C enrichment specifically at the MZF1 5ʹ-UTR, with concomitant CDS depletion, establishing spatial regulation of RNA methylation influencing splice variant expression (Fig. 3g, h). These findings preliminarily suggest that the differential expression of MZF1^S^ may be associated with NSUN7-mediated RNA m^5^C regulation. To directly validate the function of MZF1^L^ and examine whether it act as a downstream effector of NSUN7, we performed CRISPR–Cas9-mediated gene knockout (MZF1-KO) combined with isoform-specific rescue (MZF1-KO + MZF1^L^) experiments. This approach allowed to cleanly assess the functional contribution of exogenous MZF1^L^ alone in the absence of interference from endogenous MZF1 background expression. Our results demonstrated that re-expression of MZF1^L^ alone in MZF1-KO cells reduced the p-EGFR/EGFR ratio to the lowest level and significantly inhibited cell viability, whereas co-overexpression of NSUN7 did not further enhance this effect (Supplementary Fig. 8). These results collectively demonstrated the critical role of MZF1^L^ in mediating EGFR-TKI sensitivity, positioning it as a key downstream effector in the epigenetic regulatory axis.

### NSUN7/YBX1 complex promotes the expression of MZF1^S^

**Fig. 4:**
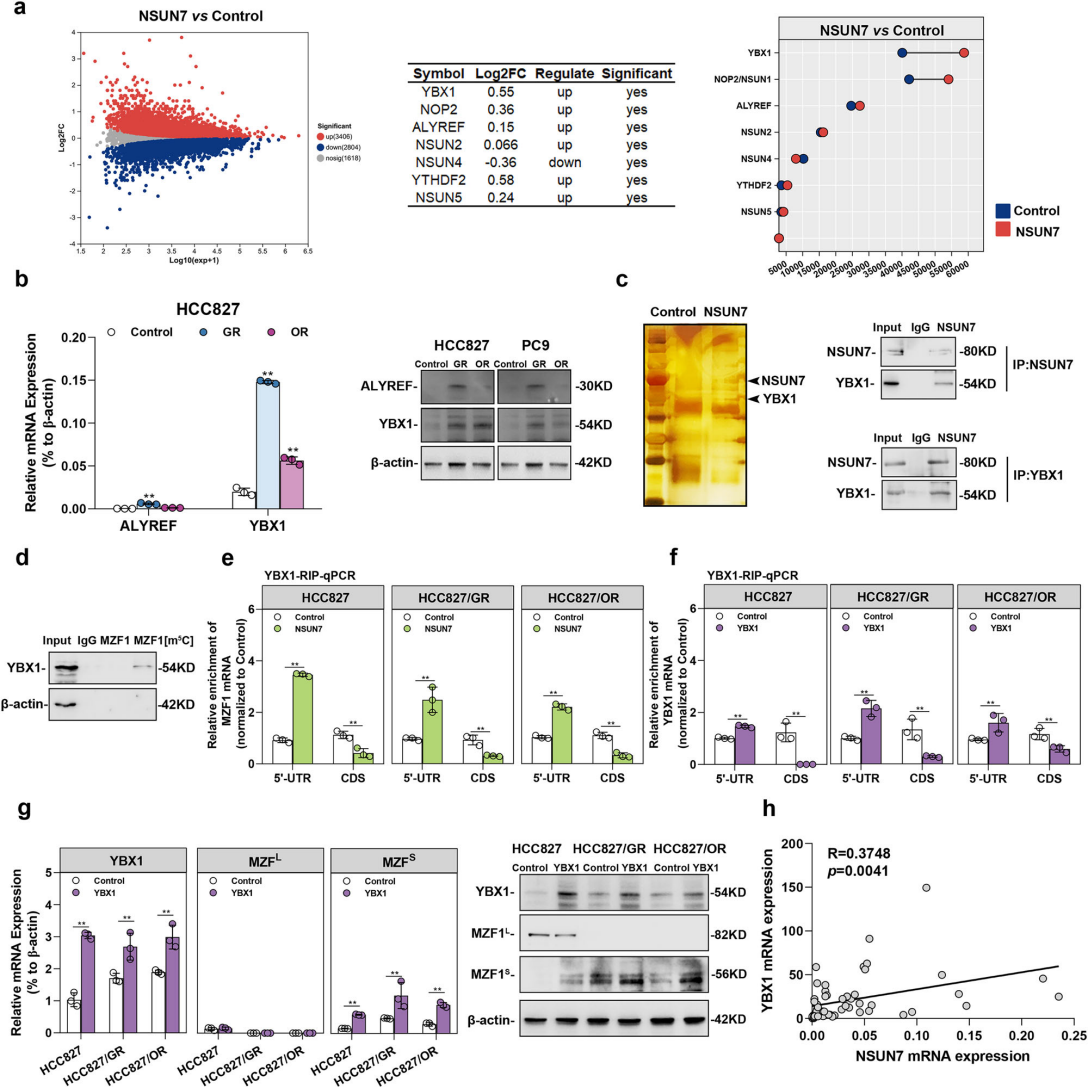
NSUN7 recruited YBX1 to regulate MZF1 alternative splicing. **a** Astral DIA-seq analysis of NSUN7-overexpressing cells. **b** RT–qPCR and western blot analysis of ALYREF and YBX1 mRNA expression in sensitive and resistant cells. **c** Co-immunoprecipitation assay detecting NSUN7–YBX1 protein interaction. **d** RNA pull-down assay confirming YBX1 binding to m^5^C-modified myeloid zinc finger 1 (MZF1). RIP-qPCR analysis of YBX1 enrichment at MZF1 5ʹ-UTR and CDS regions in NSUN7-overexpressing (part **e**) and YBX1-overexpressing (part **f**) sensitive and resistant cells. **g** RT–qPCR and western blot analysis of MZF1 splice variants following YBX1 overexpression. **h** Pearson correlation analysis of NSUN7 and YBX1 expressions in clinical specimens. *P*-values were calculated by a *t* test (parts **e**–**g**) and one‑way analysis of variance with Tukey’s post hoc test (part **b**). FC, fold change; GR, gefitinib-resistant; OR, osimertinib-resistant; qPCR, quantitative PCR; UTR, untranslated region; CDS Coding sequence, RIP-qPCR RNA Immunoprecipitation combined with quantitative real-time PCR. *P < 0.05 and **P < 0.01, significant differences from the control.

We further explored the impact of RNA methylation readers on the differential expression of MZF1 splice variants. Investigation of m^5^C reader proteins revealed YBX1 as a key mediator through proteomic profiling, showing marked upregulation of ALYREF and YBX1 following NSUN7 overexpression (Fig. 4a). Subsequent analysis confirmed elevated YBX1 expression in HCC827/GR and HCC827/OR cells, in which it formed stable protein complexes with NSUN7 (Fig. 4b, c). We preformed RNA pull-down assay using m^5^C-MZF1 RNA probes and confirmed the preferential binding of YBX1 to m^5^C-MZF1 probes by western blot (Fig. 4d). The DMR-specific regulation of MZF1 splicing variants was detected using RIP-qPCR, and NSUN7 was overexpressed in both sensitive and resistant cells. The results indicated a significant increase in the enrichment of YBX1 in the 5ʹ-UTR, whereas the opposite trend was observed in the CDS region, aligning with the enrichment of NSUN7 fragments. These results suggested that YBX1 binds to NSUN7 to exert a regulatory effect (Fig. 4e, f). Furthermore, we found that YBX1 overexpression selectively upregulated MZF1^S^ without affecting MZF1^L^ (Fig. 4g). Clinical validation demonstrated significant NSUN7-YBX1 co-expression, establishing their coordinated regulation in resistance contexts (*R* = 0.3748, *P* = 0.0041; Fig. 4h).

### NSUN7/YBX1 induces competitive binding of SRSF1 and SRSF3 to promoting MZF1^S^ expression

**Fig. 5:**
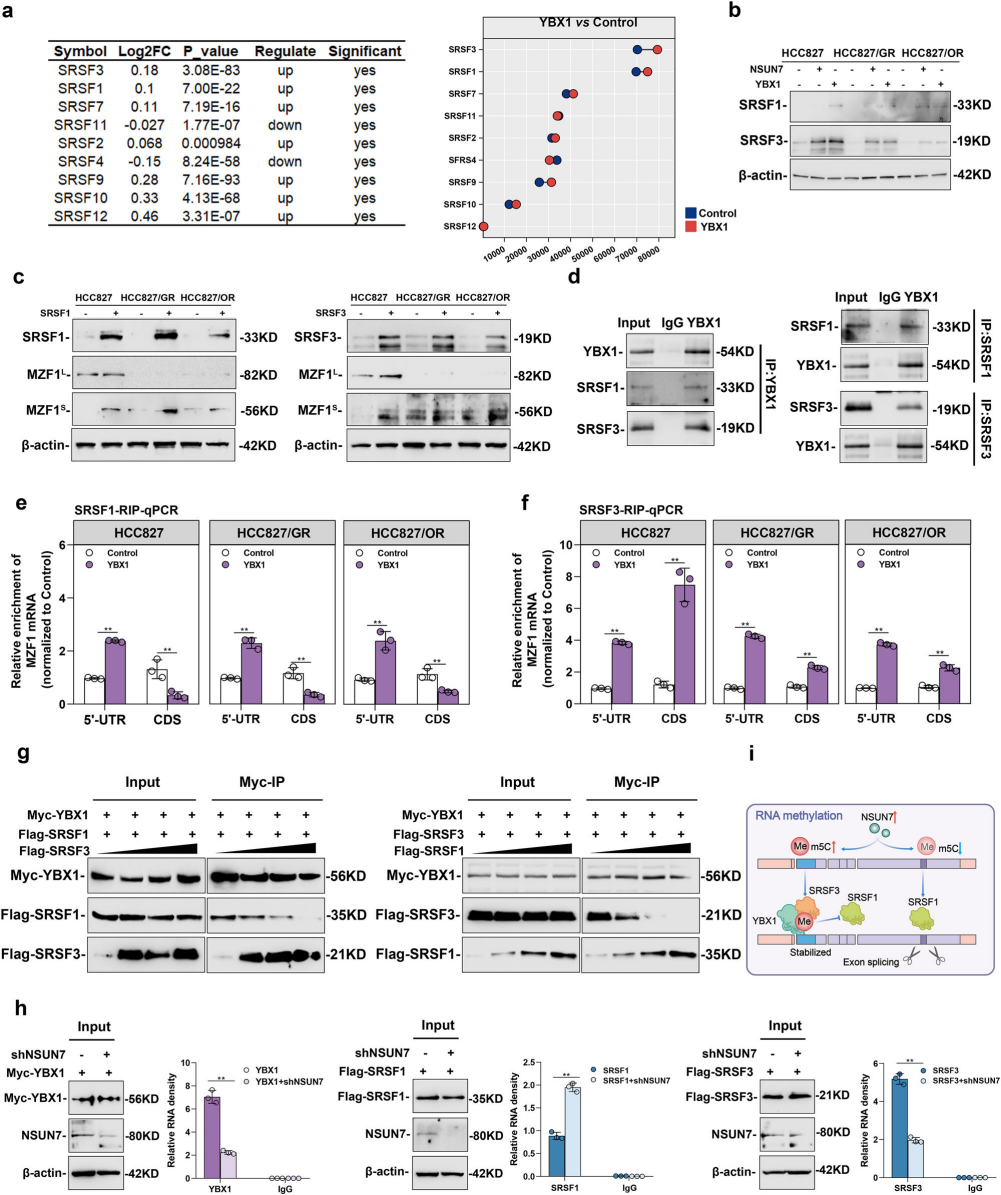
NSUN7/YBX1 recruited SRSF1 and SRSF3 to regulate MZF1 alternative splicing. **a** Quantitative proteomic analysis (DIA-seq) analysis of YBX1-overexpressing cells. **b** Western blot analysis of SRSF1 and SRSF3 expression following NSUN7 or YBX1 overexpression in HCC827, HCC827/GR, and HCC827/OR cells. **c** Western blot analysis of MZF1^L^ and MZF1^S^ expression following SRSF1 or SRSF3 overexpression in HCC827, HCC827/GR, and HCC827/OR cells. **d** Co-immunoprecipitation assay detected YBX1 interactions with SRSF1 and SRSF3. RNA immunoprecipitation (IP)-qPCR analysis showed SRSF1 (part **e**) and SRSF3 (part **f**) enrichment at MZF1 5ʹ-UTR and CDS regions following YBX1 overexpression in sensitive and resistant cells. **g** Competitive binding assay between Flag-SRSF1/Flag-SRSF3 and Myc-YBX1 in vitro. Left: constant Flag-SRSF1 with increasing Flag-SRSF3. Right: constant Flag-SRSF3 with increasing Flag-SRSF1. **h** Western blot analysis of input samples and CLIP-qPCR detection of RNA signal binding following NSUN7 knockdown. **i** Schematic diagram of NSUN7/YBX1-induced competitive binding of SRSF1 and SRSF3 to promoting MZF1^S^ expression. *P-*values were calculated by a *t* test (parts **e**, **f**, and **h**). FC, fold change; GR, gefitinib-resistant; MZF1, myeloid zinc finger 1; OR, osimertinib-resistant; qPCR, quantitative PCR; UTR, untranslated region; CDS Coding sequence; RIP-qPCR RNA Immunoprecipitation combined with quantitative real-time PCR. *P < 0.05 and **P < 0.01, significant differences from the control.

To explore the binding proteins of YBX1, we conducted mass spectrometry analysis on overexpressed YBX1. The expression of SRSF1, SRSF3, SRSF9, and SRSF10 was significantly enhanced, while SRSF4 and SRSF11 was significantly decreased after overexpressing YBX1 (Fig. 5a). Furthermore, RT–qPCR and western blot were used to validate SRSFs with elevated expression, revealing that the expression of SRSF1 and SRSF3 was upregulated by the overexpression of NSUN7 or YBX1 (Fig. 5b and Supplementary Fig. 9). We observed that SRSF1/SRSF3 overexpression selectively enhanced MZF1^S^ expression without altering MZF1^L^ (Fig. 5c). Co-IP results indicated that both SRSF1 and SRSF3 were capable of forming complexes with YBX1 (Fig. 5d). RIP-qPCR analysis revealed SRSF1 preferentially bound to 5ʹ-UTR DMRs with CDS avoidance, mirroring the binding pattern of YBX1, while SRSF3 exhibited pan-regional association (Fig. 5e, f). As both SRSF1 and SRSF3 in the 5’-UTR bind to YBX1, however they exhibited inconsistency in regulating MZF1 alternative splicing, and these suggested that the binding of SRSF3 and SRSF1 to YBX1 might involve a more complex mechanism. In vitro co-IP experiments revealed that SRSF1 and SRSF3 bind to YBX1 competitively (Fig. 5g). To determine whether the binding of SRSF3 and SRSF1 to YBX1 is dependent on NSUN7, we conducted a CLIP-qPCR by interfering expression with NSUN7. CLIP-qPCR under NSUN7-knockdown conditions revealed m^5^C-dependent YBX1/SRSF3 recruitment versus methylation-independent binding of SRSF1 (Fig. 5h). These findings established an NSUN7/YBX1-mediated competitive splicing factor recruitment mechanism governing MZF1 alternative splicing through DMR-specific interactions (Fig. 5i).

### MZF1 alternative splicing is synergistically regulated by DNA methylation and RNA m^5^C methylation modification

**Fig. 6:**
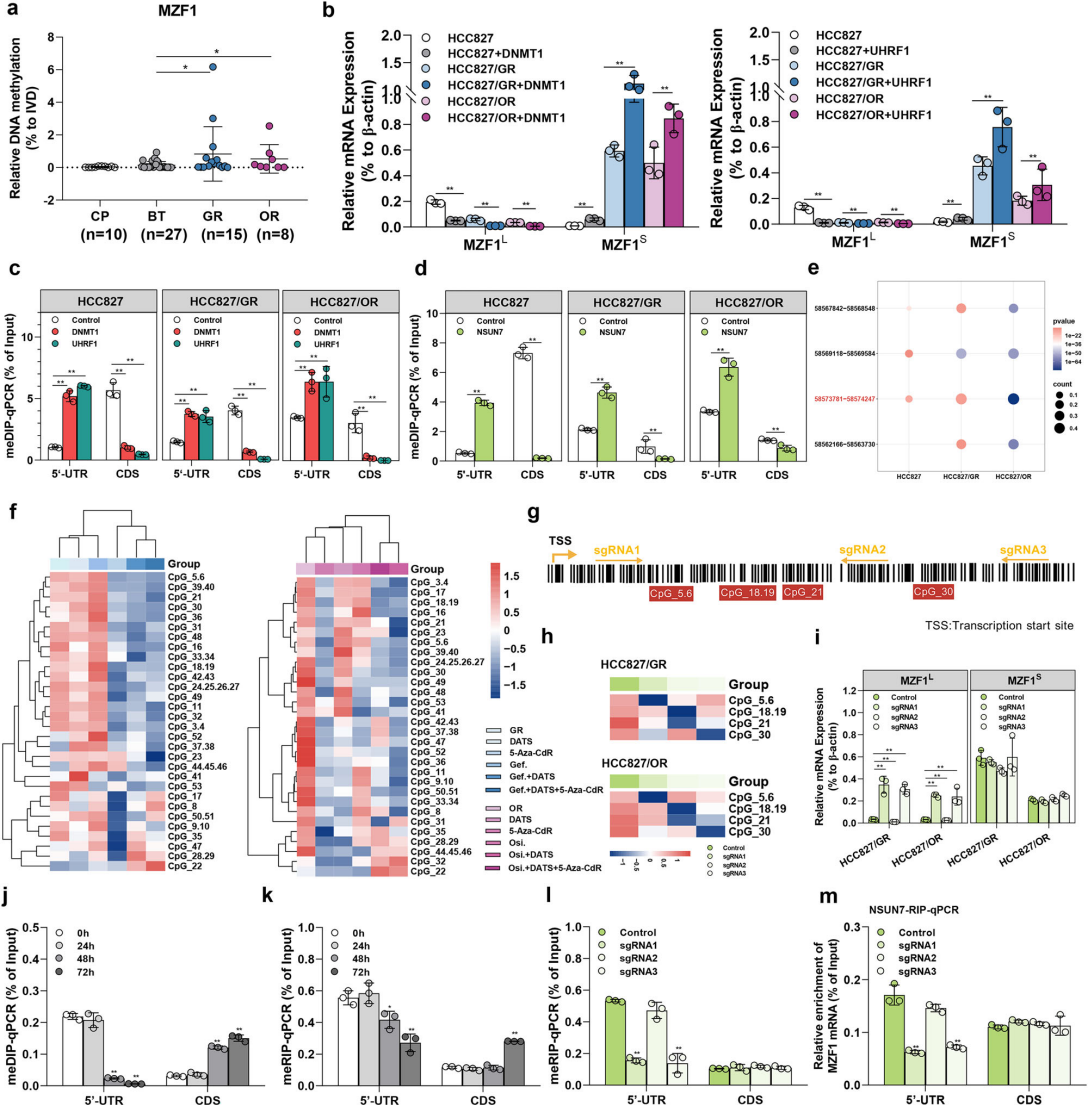
DNA 5-mC and RNA m^5^C co-regulated MZF1 alternative splicing. **a** MS–qPCR detected methylation status of myeloid zinc finger 1 (MZF1) in clinical specimens. **b** RT–qPCR analyzed the effects of UHRF1/DNMT1 overexpression on MZF1 splicing variants. Methylated DNA immunoprecipitation (MeDIP)–qPCR analysis showed 5-methylcytosine (5-mC) enrichment at MZF1 5’-UTR and CDS regions following UHRF1/DNMT1 (part **c**) and NSUN7 (part **d**) overexpression in sensitive and resistant cells. **e** Whole-genome bisulfite sequencing and RNA-Bis-Seq were performed on HCC827, HCC827/GR, and HCC827/OR to analyze the overlapping DMR regions. **f** MassArray evaluated shared DMR regions in HCC827/GR and HCC827/OR after diallyl trisulfide (DATS), 5-Aza-2ʹ-deoxycytidine (5-Aza-CdR), and/or EGFR-TKI treatment. **g**, **h** Locus-specific demethylation at the MZF1 promoter using dCas9-Tet1-CD/single guide RNA (sgRNA) systems in GR/OR cells (MassArray validation). **i** RT–qPCR assessed MZF1 splicing variant expression following site-specific demethylation. MeDIP-qPCR (part **j**) and methylated RNA immunoprecipitation (MeRIP)-qPCR (part **k**) analysis showed 5-mC enrichment at MZF1 5’-UTR and CDS regions after 5-Aza-CdR treatment in HCC827 cells (5 μM at 0, 24, 48, and 72 h). Locus-specific demethylation at the MZF1 promoter using dCas9-Tet1-CD/sgRNA systems in HCC827 cells. MeRIP–qPCR analysis showed 5-mC enrichment at MZF1 5ʹ-UTR and CDS regions (part **l**). RI-qPCR showed NSUN7 enrichment at MZF1 5’-UTR and CDS regions (part **m**). *P*-values were calculated by a *t* test (part **d**) and one‑way analysis of variance with Tukey’s post hoc test (parts **a**–**c** and **i**–**m**). BT, before treatment; CP, chronic pneumonia; GR, gefitinib-resistant; OR, osimertinib-resistant; qPCR, quantitative PCR; UTR, untranslated region; CDS Coding sequence. *P < 0.05 and **P < 0.01, significant differences from the control.

To elucidate the epigenetic mechanisms governing MZF1 splice variant expression, we investigated coordinated regulation between DNA 5-mC and RNA m^5^C methylation. Clinical specimens from GR/OR patients exhibited elevated MZF1 5ʹ-UTR DNA methylation (Fig. 6a and Supplementary Table 11). Our previous research reported that the overall expression of MZF1 was regulated by the UHRF1/DNMT1 (refs. ^11,13^). Therefore, we initially investigated whether alterations in the expression of the UHRF1/DNMT1 would influence the expression of MZF1 splicing variants. UHRF1/DNMT1 gain-of-function promoted MZF1^S^ while suppressing MZF1^L^, whereas UHRF1/DNMT1 knockdown upregulated MZF1^L^ without affecting MZF1^S^ (Fig. 6b and Supplementary Fig. 10a). MeDIP-qPCR analysis demonstrated that UHRF1/DNMT1 or NSUN7 overexpression enhanced 5-mC enrichment at the MZF1 5ʹ-UTR (Fig. 6c, d), indicating methylation crosstalk between these epigenetic modifiers. Integrative analysis of whole-genome bisulfite sequencing and RNA m^5^C bisulfite sequencing (RNA-Bis-Seq) data from HCC827, HCC827/GR, and HCC827/OR identified four consensus DMRs between resistant and sensitive cells. Cross-referencing methylation landscapes revealed a co-methylated locus (chr19:58573781–58574060, 5ʹ-UTR) exhibiting hypermethylation in resistant cells (Fig. 6e). MassArray analysis following 5-Aza-CdR treatment identified CpG_ 5.6, 18.19, 21, and 30 as dynamically regulated methylation hot spots (Fig. 6f). dCas9-Tet1-mediated locus-specific demethylation (sgRNA1 for CpG_5.6; sgRNA2 for CpG_18.19 and 21; sgRNA3 for CpG_21 and 30) confirmed methylation-dependent splicing regulation (Fig. 6g, h and Supplementary Fig. 10b). sgRNA suppressed transcription of alternative exons within the DMR locus, selectively upregulating MZF1^L^ without altering MZF1^S^ expression (Fig. 6i), confirming that DNA methylation at the MZF1 5ʹ-UTR directly represses MZF1^L^ expression. To elucidate the underlying mechanism, we investigated the hierarchical relationship between DNA methylation and RNA m^5^C modification. Time-course experiments revealed that under 5-Aza-CdR treatment, the decrease in DNA 5-mC levels preceded the reduction in RNA m^5^C levels (Fig. 6j, k). dCas9-Tet1-mediated locus-specific DNA demethylation significantly reduced RNA m^5^C levels in the 5ʹ-UTR and diminished NSUN7 recruitment to this region, whereas NSUN7 enrichment in the CDS region remained unaffected (Fig. 6l, m). These results demonstrate that DNA methylation acts as an upstream event directing RNA m^5^C deposition, forming a hierarchical regulatory network, and suggest that targeting this epigenetic axis holds therapeutic potential for restoring EGFR-TKI sensitivity.

**Fig. 7:**
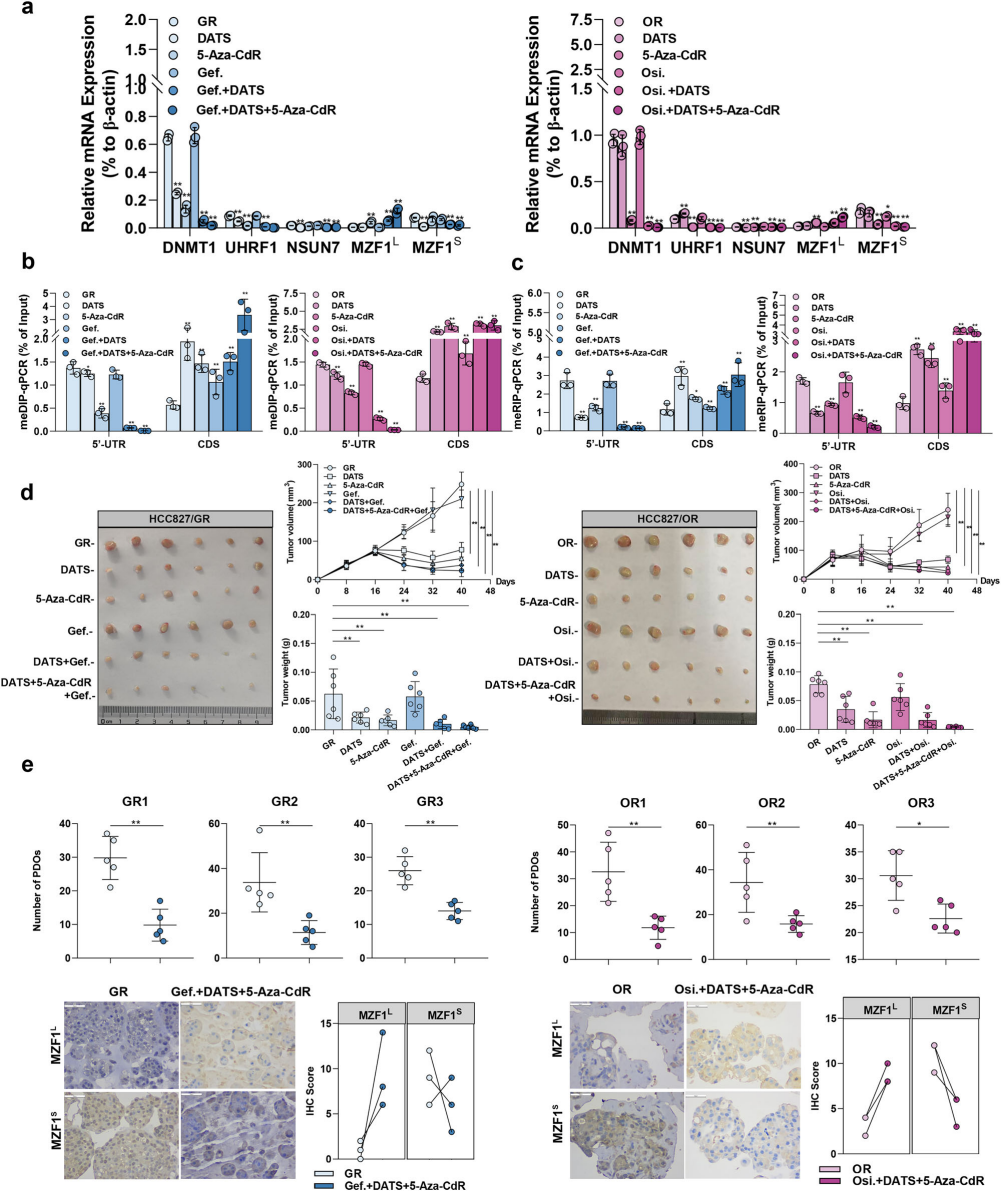
DATS combined with 5-Aza-CdR suppressed EGFR-TKIs resistance. **a** RT–quantitative PCR (qPCR) detected DNMT1, UHRF1, NSUN7, and myeloid zinc finger 1 (MZF1) splice variants after diallyl trisulfide (DATS), 5-Aza-2ʹ-deoxycytidine (5-Aza-CdR), and/or EGFR-TKI treatment. Methylated DNA immunoprecipitation (MeDIP)-qPCR (part **b**) and methylated RNA immunoprecipitation (MeRIP)-qPCR (part **c**) analysis showed 5-methylcytosine enrichment at MZF1 5’-UTR, and CDS regions after DATS, 5-Aza-CdR, and/or EGFR-TKI treatment. **d** The effect of DATS, 5-Aza-CdR, and/or EGFR-TKI treatment in vivo. Left panel: images of dissected xenograft tumors from nude mice. Right panel: tumor volume and wight of tumor-bearing mice measured. **e** Quantification of number of patient-derived organoids (PDOs) and immunohistochemistry determination of the MZF1 isoforms in PDOs. The images are shown at original magnifications of ×400. *P-*values were calculated by a *t* test (part **e**) and one‑way analysis of variance with Tukey’s post hoc test (parts **a**–**d**). GR, gefitinib-resistant; IHC, immunohistochemistry; OR, osimertinib-resistant; UTR, untranslated region; CDS Coding sequence. *P < 0.05 and **P < 0.01, significant differences from the control.

**Fig. 8:**
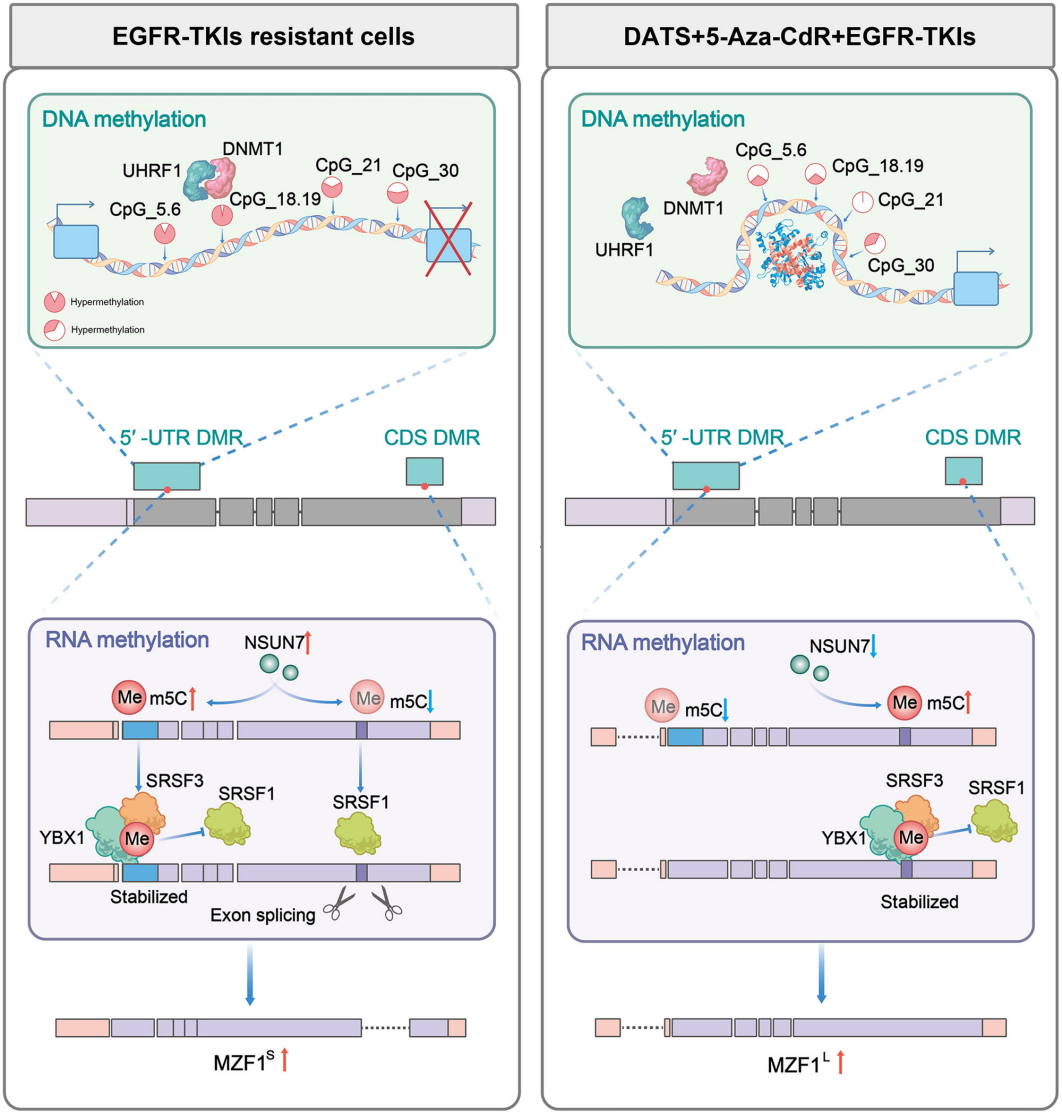
Schematic model of the dual epigenetic regulatory mechanism controlling MZF1 isoform switching in EGFR-TKI resistance. In EGFR-TKI-resistant cells, DNA methylation regulator UHRF1 recruited DNMT1 to methylate the 5ʹ-UTR, which suppressed transcription of alternative cassette exons. Concurrently, NSUN7 facilitated YBX1 binding to m^5^C-modified transcripts, forming a complex with SRSF3 that localized to m^5^C-enriched mRNA-binding domains near the 5ʹ-UTR, thereby enhancing mRNA stability in these regions. SRSF1 bound to CDS regions and mediated exon splicing, promoting MZF1^S^ expression while suppressing MZF1^L^. DATS + 5-Aza-CdR demethylation enabled methylation-sensitive zinc finger domains of transcription factors to anchor to DMR-associated motifs, restoring MZF1^L^ expression and inhibiting MZF1^S^ production. EGFR-TKI, epidermal growth factor receptor-tyrosine kinase inhibitor; MZF1, myeloid zinc finger 1; UTR, untranslated region; 5-Aza-CdR, 5-Aza-2ʹ-deoxycytidine.

Building on our previous findings that garlic-derived diallyl trisulfide (DATS) functions as an epigenetic modulator of MZF1, we treated TKI-resistant HCC827/GR and HCC827/OR with DATS, observing NSUN7 downregulation and MZF1^S^ suppression. 5-Aza-CdR monotherapy specifically enhanced MZF1^L^ expression (Fig. 7a and Supplementary Fig. 10c). Combinatorial treatment (DATS + 5-Aza-CdR) synergistically upregulated MZF1^L^ while suppressing MZF1^S^, with combination index (CI = 0.58893) confirming strong antiproliferative synergy (Supplementary Fig. 10d and Supplementary Tables 12–14). MeDIP-qPCR and MeRIP-qPCR analyses revealed that combinatorial treatment reduced 5’-UTR 5-mC enrichment (Fig. 7b, c), concomitant with suppressed proliferation/migration in resistant cells (Supplementary Fig. 11). Notably, xenograft tumor formation in nude mice and patient-derived organoids derived from resistant tissues recapitulated the combinatorial therapeutic effects observed in vitro, indicating MZF1^L^ upregulation accompanied by MZF1^S^ suppression (Fig. 7d, e). DATS and 5-Aza-CdR treatment effectively restricted the size of both the xenografts and patient-derived organoids. Gain-of-function and loss-of-function experiments confirmed that NSUN7 overexpression partially rescued, while NSUN7 knockdown potentiated, the DATS-induced reduction in RNA m^5^C modification levels and enhanced its tumor-suppressive function, demonstrating that the antitumor effects of DATS are specifically mediated through the NSUN7–RNA m^5^C epigenetic axis rather than through nonspecific pathways (Supplementary Fig. 12). These results demonstrate that the coordinated DNA 5-mC (UHRF1/DNMT1) and RNA m^5^C (NSUN7/YBX1) methylation machinery synergistically regulates MZF1 splicing through epigenetic axis crosstalk (Fig. 8).

## Discussion

Acquired resistance to EGFR-TKIs evolves through multilayered dynamic processes. Current paradigms emphasize two principal mechanisms: on-target kinase modifications (for example, EGFR C797S mutation^14^) and downstream signaling bypass via BRAF fusions, KRAS/NRAS mutations, or MAP2K1 alterations in the RAS-MAPK pathway^15,16^. Although most studies focus on genetic aberrations, emerging evidence reveals therapy-induced epigenetic reprogramming involving methylation/acetylation modifications during resistance development^17^. Such epigenetic alterations in tumor suppressor genes may represent druggable targets for resensitization strategies^18^.

Notably, ligand-independent EGFR phosphorylation during acquired resistance is accompanied by Zn^2+^ efflux and disrupted zinc homeostasis. Both hyper-zinc and hypo-zinc conditions are associated with enhanced EGFR phosphorylation and internalization, ultimately reducing TKI sensitivity^7,8^. Zinc finger proteins such as MZF1, a Krüppel-like transcription factor regulating proliferation/differentiation, maintain zinc homeostasis through Zn^2+^-dependent structural stabilization^19^. Our prior work identified MZF1 as an epigenetic tumor suppressor and inhibited EGFR phosphorylation/internalization^10,11,13^. Specifically, MZF1 overexpression sustains zinc homeostasis by enhancing Zn^2+^ chelation, positioning it as both a potential epigenetic biomarker and a therapeutic target for overcoming TKI resistance. The MZF1 gene produces two splice variants, MZF1^L^ (full-length isoform 1) containing functional domains (acidic, SCAN, transactivation, and 13 C_2_H_2_ zinc fingers recognizing CG-rich motifs) as a methylation-sensitive transcription factor, and MZF1^S^ (truncated isoform 2), lacking C_2_H_2_ zinc fingers^9,13^. Our prior work demonstrated distinct DNA-binding capacities between these two isoforms owing to differential zinc finger integrity, critically influencing transcriptional regulation^13^. Given the Zn^2+^-dependent clustering of MZF1 zinc fingers and their methylation sensitivity, we analyzed isoform-specific regulatory roles in EGFR phosphorylation. Clinical analyses of BALF and tumor tissues revealed MZF1^S^ upregulation in TKI-resistant patients versus predominant MZF1^L^ expression in treatment-sensitive cohorts. Functional studies confirmed that MZF1^S^ promoted acquired resistance by enhancing EGFR phosphorylation/internalization, whereas MZF1^L^ overexpression restored drug sensitivity through EGFR pathway suppression. MZF1^L^ binds intracellular Zn^2+^, leading to reduced free Zn²⁺ concentration, correlated with decreased EGFR phosphorylation and ultimately enhanced sensitivity to EGFR-TKIs. These findings underscored the biological significance of dissecting tissue-specific MZF1 splice variant regulation, offering dual diagnostic biomarkers and therapeutic targets for precision NSCLC management.

It should be noted that Zn^2+^ may influence EGFR phosphorylation through multiple indirect pathways. Zn^2+^ has been shown to activate the non-receptor tyrosine kinase c-Src, which in turn induced EGFR phosphorylation via Src-mediated transactivation^20,21^. Zn^2+^ also enhanced EGFR phosphorylation signaling by inhibiting the activity of protein tyrosine phosphatases such as PTP1B and SHP-1. Previous study demonstrated in vitro that IC_50_ values of zinc for PTP1B and SHP-1 were as low as 17 nM and 93 nM, and that an increase in intracellular zinc concentration significantly elevated global protein tyrosine phosphorylation levels^22,23^. This mechanism suggested that Zn^2+^ led to sustained activation of EGFR and Src by suppressing the negative regulatory function of PTPs. Furthermore, Zn^2+^ might indirectly activate EGFR by promoting the shedding of the EGFR ligand heparin-binding EGF-like growth factor (HB-EGF). Studies showed that Zn^2+^ exposure induced HB-EGF release from cells, an effect that was significantly blocked by an anti-HB-EGF-neutralizing antibody. Zn^2+^ stimulation activated matrix metalloproteinase (MMP)-3, and inhibition of MMP-3 activity abrogated Zn^2+^-induced EGFR phosphorylation and HB-EGF release, suggesting that Zn^2+^ triggers paracrine/autocrine activation of EGFR through MMP-3-mediated HB-EGF shedding^24^. Collectively, these mechanisms indicated that the regulation of EGFR phosphorylation by Zn^2+^ was a multifaceted process involving intracellular signaling kinase activation, phosphatase inhibition, and extracellular ligand shedding. Although our findings established a clear association between MZF1 isoform-driven Zn^2+^ homeostasis changes and EGFR phosphorylation status, the precise molecular link between Zn^2+^ and EGFR through the aforementioned signaling intermediates remained to be determined.

In contrast to irreversible genetic mutations, the progressive and reversible nature of epigenetic alterations holds significant potential for therapeutic monitoring and intervention in tumorigenesis and drug resistance^25^. Although post-transcriptional RNA modifications such as m^6^A have been extensively studied in alternative splicing regulation and oncogenesis, emerging evidence implicates aberrant RNA m^5^C methylation in tumor progression and therapeutic resistance^26^. The mechanistic interplay between RNA and DNA methylation remained unclear, with only four studies reporting m^6^A-DNA methylation crosstalk^27–30^: two demonstrated co-occurrence of m^6^A and 5-mC at identical genomic loci^27,28^, whereas others linked m^6^A readers to TET-mediated DNA hydroxymethylation^29,30^. Notably, the synergistic regulation of specific gene transcription through coordinated RNA m^5^C and DNA 5-mC methylation remained unreported. Although both modifications influenced alternative splicing^13,31,32^, their potential cooperation in modulating splice variants to drive cancer resistance constituted a critical unresolved question in epigenetic oncology.

Our study revealed that EGFR-TKI resistance correlates with elevated RNA m^5^C modification and differential MZF1 splice variant expression. Although NSUN2/NSUN6 are established m^5^C methyltransferases^33,34^, we identified low-abundance NSUN7 as the predominant regulator of MZF1 splicing, suppressing MZF1^L^ while promoting MZF1^S^. However, NSUN7 knockdown incompletely reversed this phenotype, suggesting auxiliary regulatory mechanisms. The enzymatic targets of NSUN7 remained undefined^35^. DMR analysis demonstrated NSUN7-mediated m^5^C enrichment at the MZF1 5ʹ-UTR with concurrent CDS depletion, implicating spatial methylation patterns in MZF1^S^ regulation. The m^5^C reader YBX1, which preferentially binds methylated RNA^36^, formed complexes with NSUN7 to modulate MZF1^S^ expression. Despite consistent YBX1 binding to MZF1 DMRs across sensitive/resistant cells, isoform regulation diverged. Proteomic screening identified competitive interactions between YBX1 and SR family splicing factors^37^: SRSF1 (exon retention) versus SRSF3 (exon skipping). Our findings suggested that SRSF1/SRSF3 competes for NSUN7/YBX1 binding to differentially regulate MZF1^S^ expression.

We further investigated the regulatory mechanisms underlying differential MZF1^L^ expression. Previous studies demonstrated that RNA methylation induced adjacent DNA demethylation to modulate chromatin accessibility and gene expression^29^, whereas DNA methylation reciprocally regulates RNA methyltransferase activity^38^. Clinical specimens from resistant cohorts exhibited elevated DNA methylation at the MZF1 5ʹ-UTR, with UHRF1/DNMT1 knockdown specifically restoring MZF1^L^ expression. Integrative analysis of whole-genome bisulfite sequencing and RNA m^5^C Bis-seq identified co-methylated DMRs (DNA 5-mC/RNA m^5^C overlapped). Site-specific methylation editing at these loci suppressed alternative cassette exon transcription, reducing MZF1^L^ levels. These results suggested coordinated DNA/RNA methylation silences MZF1^L^, establishing a unique regulatory paradigm combining DNA 5-mC-mediated transcriptional control with RNA m^5^C-dependent post-transcriptional regulation. Elucidating this synergistic epigenetic mechanism governing MZF1 alternative splicing might yield dual-purpose biomarkers for EGFR-TKI resistance and therapeutic targets for resensitization strategies.

The DNA-hypomethylating agent 5-Aza-2ʹ-deoxycytidine (5-Aza-CdR) has demonstrated clinical utility in cancer therapy^39,40^. Preclinical studies indicated synergistic effects between EGFR-TKI and 5-Aza-CdR, enhancing therapeutic response depth^41^. However, 5-Aza-CdR’s nonspecific DNA demethylation induces genome-wide epigenetic drift, potentially reactivating oncogenes while demethylating tumor suppressors^42,43^. Our prior work revealed its limited efficacy at constitutively methylated CpGs within critical regulatory regions, such as methylation-sensitive transcription factor binding motifs^13^. Concurrently, RNA m^5^C modifications promoted 5-Aza-CdR resistance, with clinically resistant specimens showing elevated RNA m^5^C levels and NSUN1/BRD4-associated chromatin activation^44^, posing challenges for epigenetic interventions.

The garlic-derived compound DATS, a recognized anticancer agent^45,46^, lacks direct DNA demethylation activity but enhances precision of 5-Aza-CdR at recalcitrant CpGs^11,13,46^. These recalcitrant CpGs frequently reside in tissue-specific regulatory regions (for example, DMRs). DATS co-treatment promotes targeted demethylation, facilitating DNA looping to anchor methylation-sensitive transcription factors. Our study found that DATS suppressed the RNA m^5^C complex NSUN7/YBX1. Combined 5-Aza-CdR/DATS treatment synergistically upregulated MZF1^L^ while suppressing MZF1^S^, significantly restoring EGFR-TKI sensitivity in resistant models. Our study uncovered a hierarchical epigenetic regulatory mechanism underlying EGFR-TKI resistance, namely, the hierarchical coupling between DNA methylation and RNA m⁵C modification. Although previous studies investigated DNA methylation and RNA methylation as independent regulatory events, our findings demonstrated that during the acquisition of drug resistance, DNA 5-mC methylation preceded and hierarchically directed the deposition of RNA m^5^C modifications. This *cis-*regulatory pattern differed from previously reported linear models and proposed a novel paradigm of cross-layer epigenetic cascade regulation. More importantly, we revealed that this hierarchical regulatory axis directly mediated drug resistance by driving alternative splicing isoform switching of MZF1. In contrast to existing studies that had primarily focussed on the silencing or activation of gene expression levels, we demonstrated that the UHRF1/DNMT1–NSUN7/YBX1 axis regulated the methylation status of MZF1, thereby achieving a switch from the functional MZF1^L^ to the truncated MZF1^S^. This resistance mechanism provided a novel perspective for understanding the complexity of EGFR-TKI resistance. From a translational perspective, this hierarchical regulatory axis offered a targetable splicing switch. Intervention with DATS combined with 5-Aza-CdR restored MZF1^L^ expression and reversed drug resistance, suggesting that targeting this epigenetic axis might represent a novel strategy for overcoming EGFR-TKI resistance.

In conclusion, by dissecting the non-genomic contributions of MZF1 transcript variants to functional heterogeneity, we found that NSUN7/YBX1 elevated RNA m^5^C methylation, recruited SRSF1 and SRSF3 for competitive binding, and collaborated with DNA 5-mC to enhance the expression of MZF1^S^, thereby facilitating EGFR-TKI resistance. The natural product DATS in combination with the epigenetic drug 5-Aza-CdR was capable of inhibiting RNA m^5^C and DNA 5-mC methylation, promoting the expression of MZF1^L^, and reversing the acquired resistance of EGFR-TKIs.

## Supplementary information

Supplementary Information

## Data Availability

Data are available from the corresponding author upon reasonable request.

## References

[1] Siegel, R. L., Giaquinto, A. N. & Jemal, A. Cancer statistics, 2024. *CA Cancer J. Clin.* **74**, 12–49 (2024). 38230766 10.3322/caac.21820

[2] Cooper AJ, Sequist LV, Lin JJ. Third-generation EGFR and ALK inhibitors: mechanisms of resistance and management. *Nat Rev Clin. Oncol.*10.1038/s41571-022-00639-9 (2022). 10.1038/s41571-022-00639-9 PMC9621058 35534623

[3] Chen, Z. et al. Long non-coding RNA CASC9 promotes gefitinib resistance in NSCLC by epigenetic repression of DUSP1. *Cell Death Dis.* **11**, 858 (2020). 33056982 10.1038/s41419-020-03047-y PMC7560854

[4] Raoof, S. et al. Targeting FGFR overcomes EMT-mediated resistance in EGFR mutant non-small cell lung cancer. *Oncogene* **38**, 6399–6413 (2019). 31324888 10.1038/s41388-019-0887-2 PMC6742540

[5] Avram, G. E. et al. Changes in global DNA methylation and hydroxymethylation in oral mucosa according to tobacco smoke exposure. *J. Int. Med. Res.* **48**, 300060520954677 (2020). 32938281 10.1177/0300060520954677 PMC7503033

[6] Cheng, F. J. et al. Cigarette smoke-induced LKB1/AMPK pathway deficiency reduces EGFR TKI sensitivity in NSCLC. *Oncogene* **40**, 1162–75 (2021). 33335306 10.1038/s41388-020-01597-1 PMC7878190

[7] Barth LM, Rink L, Wessels I. Increase of the intracellular zinc concentration leads to an activation and internalisation of the epidermal growth factor receptor in A549 cells. *Int. J. Mol. Sci.*10.3390/ijms22010326 (2020). 10.3390/ijms22010326 PMC7795919 33396916

[8] Scheiermann E, Puppa MA, Rink L, Wessels I. Zinc status impacts the epidermal growth factor receptor and downstream protein expression in A549 cells. *Int. J. Mol. Sci.*10.3390/ijms23042270 (2022). 10.3390/ijms23042270 PMC8876057 35216384

[9] Brix, D. M., Bundgaard Clemmensen, K. K. & Kallunki, T. Zinc finger transcription factor MZF1-A specific regulator of cancer invasion. *Cells* **9**, 223 (2020). 31963147 10.3390/cells9010223 PMC7016646

[10] Lin, S. et al. Acquired resistance to EGFR-TKIs in NSCLC mediates epigenetic downregulation of MUC17 by facilitating NF-κB activity via UHRF1/DNMT1 complex. *Int. J. Biol. Sci.* **19**, 832–851 (2023). 36778111 10.7150/ijbs.75963 PMC9910003

[11] Lin, S. et al. Transcription factor myeloid zinc-finger 1 suppresses human gastric carcinogenesis by interacting with metallothionein 2A. *Clin. Cancer Res.* **25**, 1050–62 (2019). 30301827 10.1158/1078-0432.CCR-18-1281

[12] Lin, B.et al. Epigenetic silencing of PRSS3 provides growth and metastasis advantage for human hepatocellular carcinoma. *J. Mol. Med.* **95**, 1237–49 (2017). 28844099 10.1007/s00109-017-1578-5 PMC8171496

[13] Lin, S. et al. UHRF1/DNMT1-MZF1 axis-modulated intragenic site-specific CpGI methylation confers divergent expression and opposing functions of PRSS3 isoforms in lung cancer. *Acta Pharm. Sin. B* **13**, 2086–2106 (2023). 37250150 10.1016/j.apsb.2023.02.015 PMC10213989

[14] Thress, K. S. et al. Acquired EGFR C797S mutation mediates resistance to AZD9291 in non-small cell lung cancer harboring EGFR T790M. *Nat. Med.* **21**, 560–562 (2015). 25939061 10.1038/nm.3854 PMC4771182

[15] Schoenfeld, A. J. et al. Tumor analyses reveal squamous transformation and off-target alterations as early resistance mechanisms to first-line osimertinib in EGFR-mutant lung cancer. *Clin. Cancer Res.* **26**, 2654–2663 (2020). 31911548 10.1158/1078-0432.CCR-19-3563 PMC7448565

[16] Pailler, E. et al. Acquired resistance mutations to ALK inhibitors identified by single circulating tumor cell sequencing in ALK-rearranged non-small-cell lung cancer. *Clin. Cancer Res.* **25**, 6671–6682 (2019). 31439588 10.1158/1078-0432.CCR-19-1176

[17] Su, SF. et al. Genome-wide epigenetic landscape of lung adenocarcinoma links HOXB9 DNA methylation to intrinsic EGFR-TKI resistance and heterogeneous responses. *JCO Precis. Oncol.*10.1200/po.20.00151 (2021). 10.1200/PO.20.00151 PMC8140798 34036228

[18] Hou, T. et al. Decitabine reverses gefitinib resistance in PC9 lung adenocarcinoma cells by demethylation of RASSF1A and GADD45β promoter. *Int. J. Clin. Exp. Pathol.* **12**, 4002–4010 (2019). 31933796 PMC6949799

[19] Liang, J. et al. Transcription factor ZNF263 enhances EGFR-targeted therapeutic response and reduces residual disease in lung adenocarcinoma. *Cell Rep.* **43**, 113771 (2024). 38335093 10.1016/j.celrep.2024.113771

[20] Kocyła A, Krężel A. Zinc-mediated dynamics of CD4/CD8α co-receptors and Lck kinase: implications for zinc homeostasis, immune response, and biotechnological innovations. *Metallomics*. 10.1093/mtomcs/mfaf018 (2025). 10.1093/mtomcs/mfaf018 PMC12198760 40504517

[21] Hirano, T., Murakami, M., Fukada, T., Nishida, K., Yamasaki, S. & Suzuki, T. Roles of zinc and zinc signaling in immunity: zinc as an intracellular signaling molecule. *Adv. Immunol.* **97**, 149–176 (2008). 18501770 10.1016/S0065-2776(08)00003-5

[22] Samet, J. M., Dewar, B. J., Wu, W. & Graves, L. M. Mechanisms of Zn(2+)-induced signal initiation through the epidermal growth factor receptor. *Toxicol. Appl. Pharm.* **191**, 86–93 (2003). 10.1016/s0041-008x(03)00219-9 12915106

[23] Haase, H. & Maret, W. Intracellular zinc fluctuations modulate protein tyrosine phosphatase activity in insulin/insulin-like growth factor-1 signaling. *Exp. Cell Res.* **291**, 289–298 (2003). 14644152 10.1016/s0014-4827(03)00406-3

[24] Wu, W. et al. Heparin-binding epidermal growth factor cleavage mediates zinc-induced epidermal growth factor receptor phosphorylation. *Am. J. Respir. Cell Mol. Biol.* **30**, 540–547 (2004). 12972402 10.1165/rcmb.2003-0233OC

[25] Kim, D., Lee, Y. S., Kim, D. H. & Bae, S. C. Lung cancer staging and associated genetic and epigenetic events. *Mol. Cells* **43**, 1–9 (2020). 31999917 10.14348/molcells.2020.2246 PMC6999714

[26] Song, H. et al. Biological roles of RNA m(5)C modification and its implications in cancer immunotherapy. *Biomark. Res.* **10**, 15 (2022). 35365216 10.1186/s40364-022-00362-8 PMC8973801

[27] Xu, W. et al. METTL3 regulates heterochromatin in mouse embryonic stem cells. *Nature* **591**, 317–321 (2021). 33505026 10.1038/s41586-021-03210-1

[28] Quarto, G. et al. Fine-tuning of gene expression through the Mettl3–Mettl14–Dnmt1 axis controls ESC differentiation. *Cell*10.1016/j.cell.2024.12.009 (2025). 10.1016/j.cell.2024.12.009 PMC12160003 39826545

[29] Deng, S. et al. RNA m(6)A regulates transcription via DNA demethylation and chromatin accessibility. *Nat. Genet.* **54**, 1427–1437 (2022). 36071173 10.1038/s41588-022-01173-1

[30] Sun, T., Xu, Y., Xiang, Y., Ou, J., Soderblom, E. J. & Diao, Y. Crosstalk between RNA m6A and DNA methylation regulates transposable element chromatin activation and cell fate in human pluripotent stem cells. *Nat. Genet.* **55**, 1324–1335 (2023). 37474847 10.1038/s41588-023-01452-5 PMC10766344

[31] Neri, F. et al. Intragenic DNA methylation prevents spurious transcription initiation. *Nature* **543**, 72–77 (2017). 28225755 10.1038/nature21373

[32] Mendel, M. et al. Splice site m(6)A methylation prevents binding of U2AF35 to inhibit RNA splicing. *Cell* **184**, 3125–3142.e25 (2021). 33930289 10.1016/j.cell.2021.03.062 PMC8208822

[33] Fang, L. et al. CIGAR-seq, a CRISPR/Cas-based method for unbiased screening of novel mRNA modification regulators. *Mol. Syst. Biol.* **16**, e10025 (2020). 33251765 10.15252/msb.202010025 PMC7701898

[34] Selmi, T. et al. Sequence- and structure-specific cytosine-5 mRNA methylation by NSUN6. *Nucleic Acids Res.* **49**, 1006–1022 (2021). 33330931 10.1093/nar/gkaa1193 PMC7826283

[35] Aguilo, F. et al. Deposition of 5-methylcytosine on enhancer RNAs enables the coactivator function of PGC-1α. *Cell Rep.* **14**, 479–492 (2016). 26774474 10.1016/j.celrep.2015.12.043 PMC4731243

[36] Su, J. et al. NSUN2-mediated RNA 5-methylcytosine promotes esophageal squamous cell carcinoma progression via LIN28B-dependent GRB2 mRNA stabilization. *Oncogene* **40**, 5814–5828 (2021). 34345012 10.1038/s41388-021-01978-0 PMC8484015

[37] Zhu, Y. et al. Molecular mechanisms for CFIm-mediated regulation of mRNA alternative polyadenylation. *Mol. Cell* **69**, 62–74.e4 (2018). 29276085 10.1016/j.molcel.2017.11.031 PMC5756121

[38] Ortiz-Barahona, V. et al. Epigenetic inactivation of the 5-methylcytosine RNA methyltransferase NSUN7 is associated with clinical outcome and therapeutic vulnerability in liver cancer. *Mol. Cancer* **22**, 83 (2023). 37173708 10.1186/s12943-023-01785-z PMC10176850

[39] Bowler, E. H., Bell, J., Divecha, N., Skipp, P. & Ewing, R. M. Proteomic analysis of azacitidine-induced degradation profiles identifies multiple chromatin and epigenetic regulators including Uhrf1 and Dnmt1 as sensitive to azacitidine. *J. Proteome Res.* **18**, 1032–1042 (2019). 30672294 10.1021/acs.jproteome.8b00745

[40] Xu, P. et al. UHRF1 regulates alternative splicing by interacting with splicing factors and U snRNAs in a H3R2me involved manner. *Hum. Mol. Genet.* **30**, 2110–2122 (2021). 34196368 10.1093/hmg/ddab178

[41] Maiti, A., Cortes, J. E., Brown, Y. D. & Kantarjian, H. M. Phase I/II study of low-dose azacytidine in patients with chronic myeloid leukemia who have minimal residual disease while receiving therapy with tyrosine kinase inhibitors. *Leuk. Lymphoma* **58**, 722–725 (2017). 27658536 10.1080/10428194.2016.1207767 PMC5250544

[42] Liu, R., Zhao, E., Yu, H., Yuan, C., Abbas, M. N. & Cui, H. Methylation across the central dogma in health and diseases: new therapeutic strategies. *Signal Transduct. Target Ther.* **8**, 310 (2023). 37620312 10.1038/s41392-023-01528-y PMC10449936

[43] Liu, Y. C. et al. Demethylation and up-regulation of an oncogene after hypomethylating therapy. *N. Engl. J. Med.* **386**, 1998–2010 (2022). 35613022 10.1056/NEJMoa2119771 PMC9514878

[44] Cheng, J. X. et al. RNA cytosine methylation and methyltransferases mediate chromatin organization and 5-azacytidine response and resistance in leukaemia. *Nat. Commun.* **9**, 1163 (2018). 29563491 10.1038/s41467-018-03513-4 PMC5862959

[45] Pan, Y. et al. Epigenetic upregulation of metallothionein 2A by diallyl trisulfide enhances chemosensitivity of human gastric cancer cells to docetaxel through attenuating NF-κB activation. *Antioxid. Redox Signal* **24**, 839–854 (2016). 26801633 10.1089/ars.2014.6128 PMC4876530

[46] Zhang H, Wang H, Qin L, Lin S. Garlic-derived compounds: epigenetic modulators and their antitumor effects. *Phytother Res.*10.1002/ptr.8108 (2024). 10.1002/ptr.8108 38194996

