# Supplementary material for: Coordinated DNA 5-mC and RNA m5C methylation epigenetically regulates MZF1 splice variants to drive EGFR-TKI resistance
Source: Exp Mol Med. 2026 Jul 1;58(7):2138–54. doi: 10.1038/s12276-026-01758-4 (PMC13434211; doi:10.1038/s12276-026-01758-4)
Supplement: Supplementary file 1 [file 12276_2026_1758_MOESM1_ESM.pdf]

Supplementary Materials for

Coordinated DNA 5-mC and RNA m^5C Methylation Epigenetically Regulates *MZF1* Splice Variants to Drive EGFR-TKIs Resistance

Running title: Coordinated Epigenetically Regulates *MZF1* Splice Variants

Huan Zhang[1, 2#], Yutong Pang[1,2#], Bin Liu[1#], Dongfeng Wang[3#], Niying Zhao[1,2],
Xiaojue Wang[1], PanJian Wei[1], Jie Lin[4*], Shuye Lin[1,2,5*]

Correspondence to: Shuye Lin, E-mail: linshuye@foxmail.com. Jie Lin, E-mail: linjie891@163.com

This PDF file includes:

Supplementary Fig. 1 to 12

Supplementary Table 1 to 14

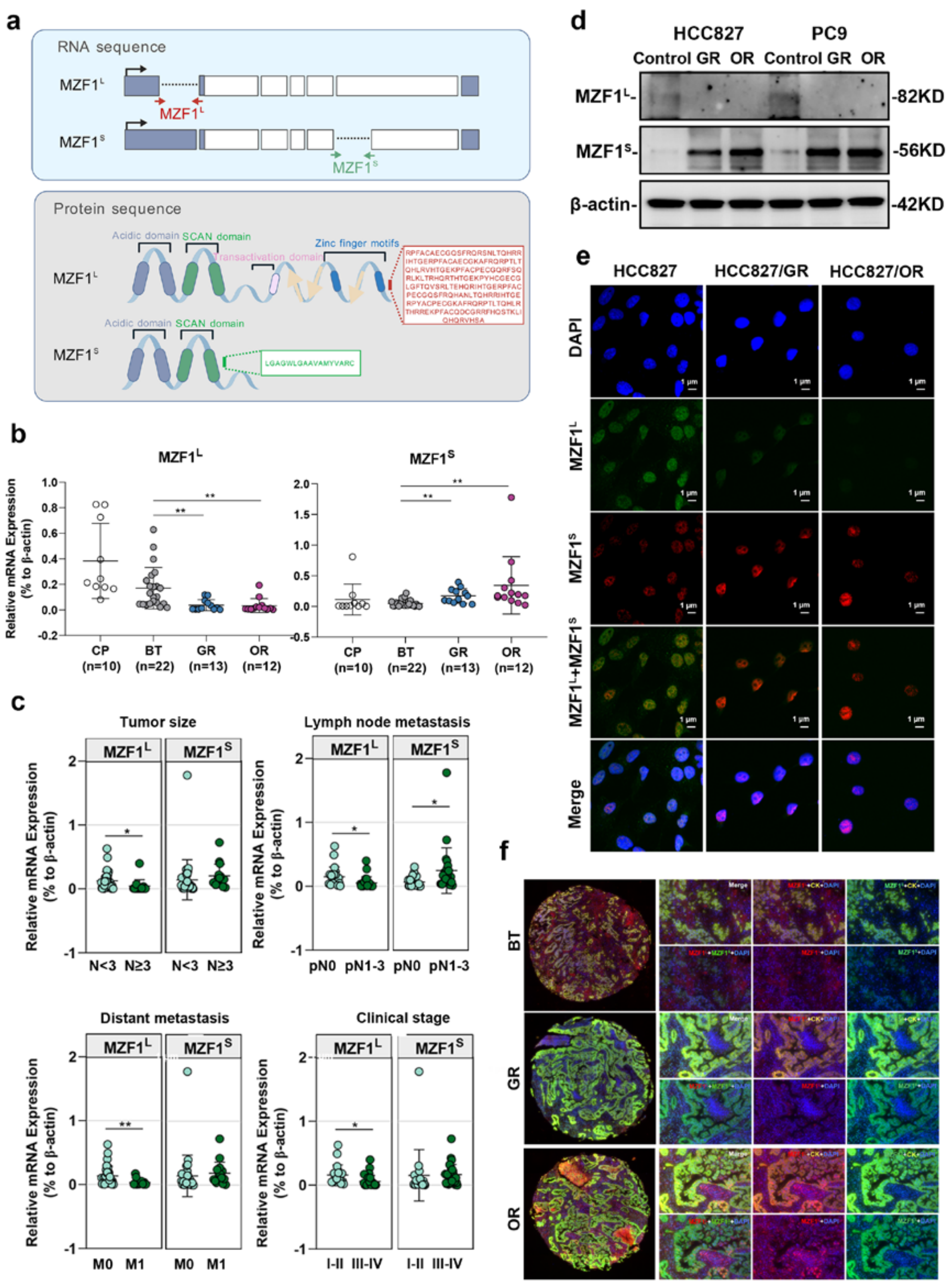

Supplementary Fig. 1 Divergent expression of MZF1 splicing variants in lung cancer cell lines and tissues. (a) Schematic of the MZF1 transcript variants and the designed RT–qPCR primers. **(b)** RT–qPCR analysis of the MZF1 transcripts expressed in BALF from patients with CP (chronic pneumonia, n=10), BT (patient before treatment, n=22), GR (gefitinib resistant patient, n=13) and OR (osimertinib resistant

patient, n=12). **(c)** RT–qPCR of MZF1 transcripts expression in BALF from patients with tumor size, lymph node metastasis, distant metastasis and clinical stage. **(d)** Western blot of the expression of $MZF1^{L}$ and $MZF1^{S}$ in gefitinib/osimertinib-resistant HCC827 and PC9 cells. **(e)** Representative immunofluorescence (IF) image showing the expression and distribution of $MZF1^{L}$(green) and $MZF1^{S}$(red) in HCC827, HCC827/GR and HCC827/OR cells. DAPI counterstaining (blue) was used to visualize nuclear DNA. Scale bar: 1 μM. **(f)** Multiplexed immunohistochemistry (mIHC) determination of the MZF1 isoforms in a commercial tissue array. MZF1 isoforms were stained with antibodies specifically against $MZF1^{L}$ (pseudocolored red), $MZF1^{S}$ (pseudocolored green), and pan-CK (pseudocolored yellow) was used to stain and identify bound tumor cells. DAPI-stained nuclei are shown in blue. The images are shown at original magnifications of × 200 and × 400. *$P < 0.05$, **$P < 0.01$, significant differences from the control.

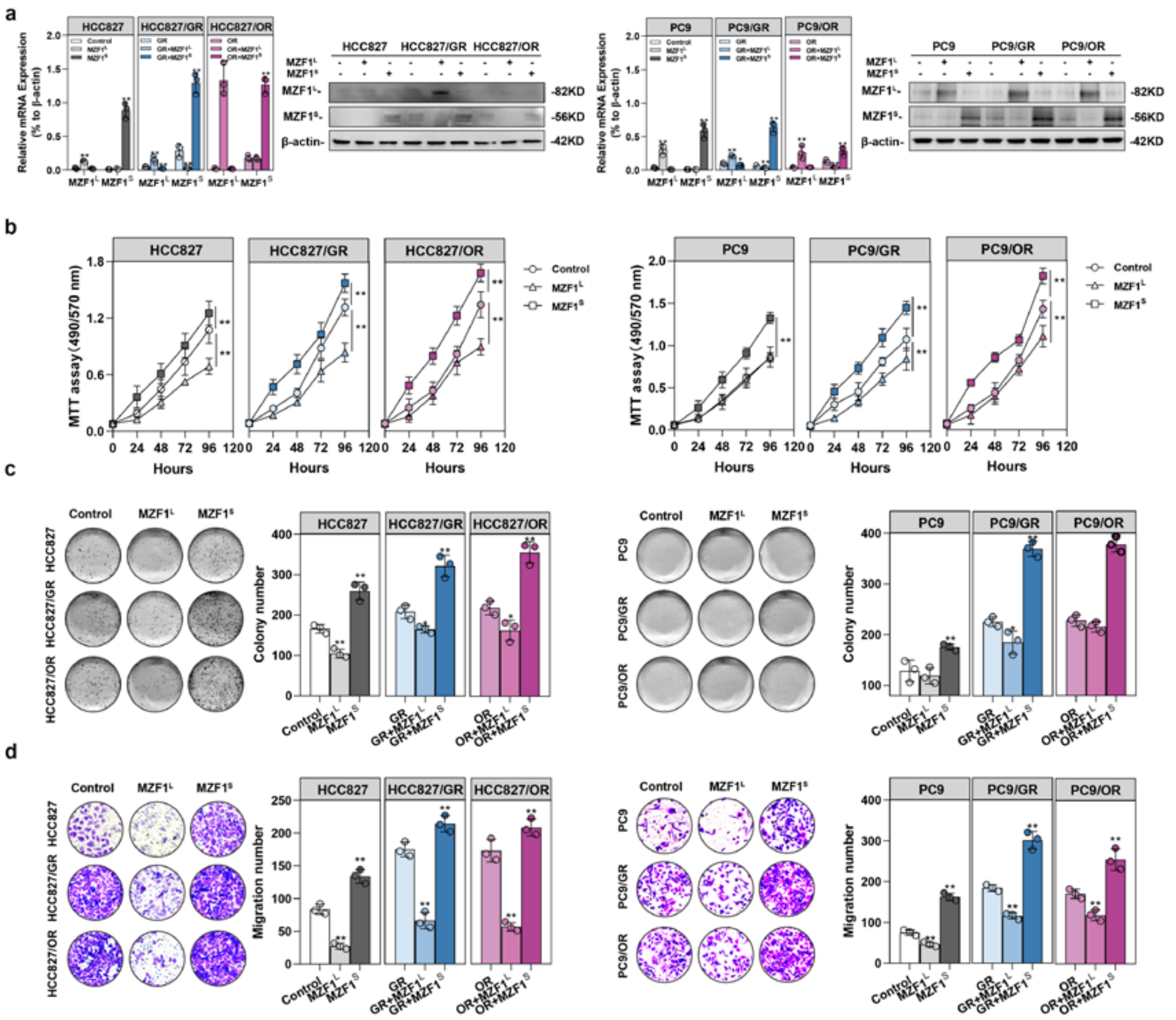

Supplementary Fig. 2 Function of MZF1 splice variant overexpression in drug-resistant cells. (a) RT-qPCR and western blot detected MZF1 splice variant expression following exogenous overexpression in sensitive and resistant cells. **(b)-(d)** Stable MZF1 splice variant-overexpressing models were established in EGFR-TKI-sensitive and -resistant cell lines. The MTT assay (b), colony formation (c), and Transwell migration assays (d) evaluated proliferative and migration capacities. Representative images (left panels) and quantitative data (right panels) are shown. **P* < 0.05, ***P* < 0.01, significant differences from the control.

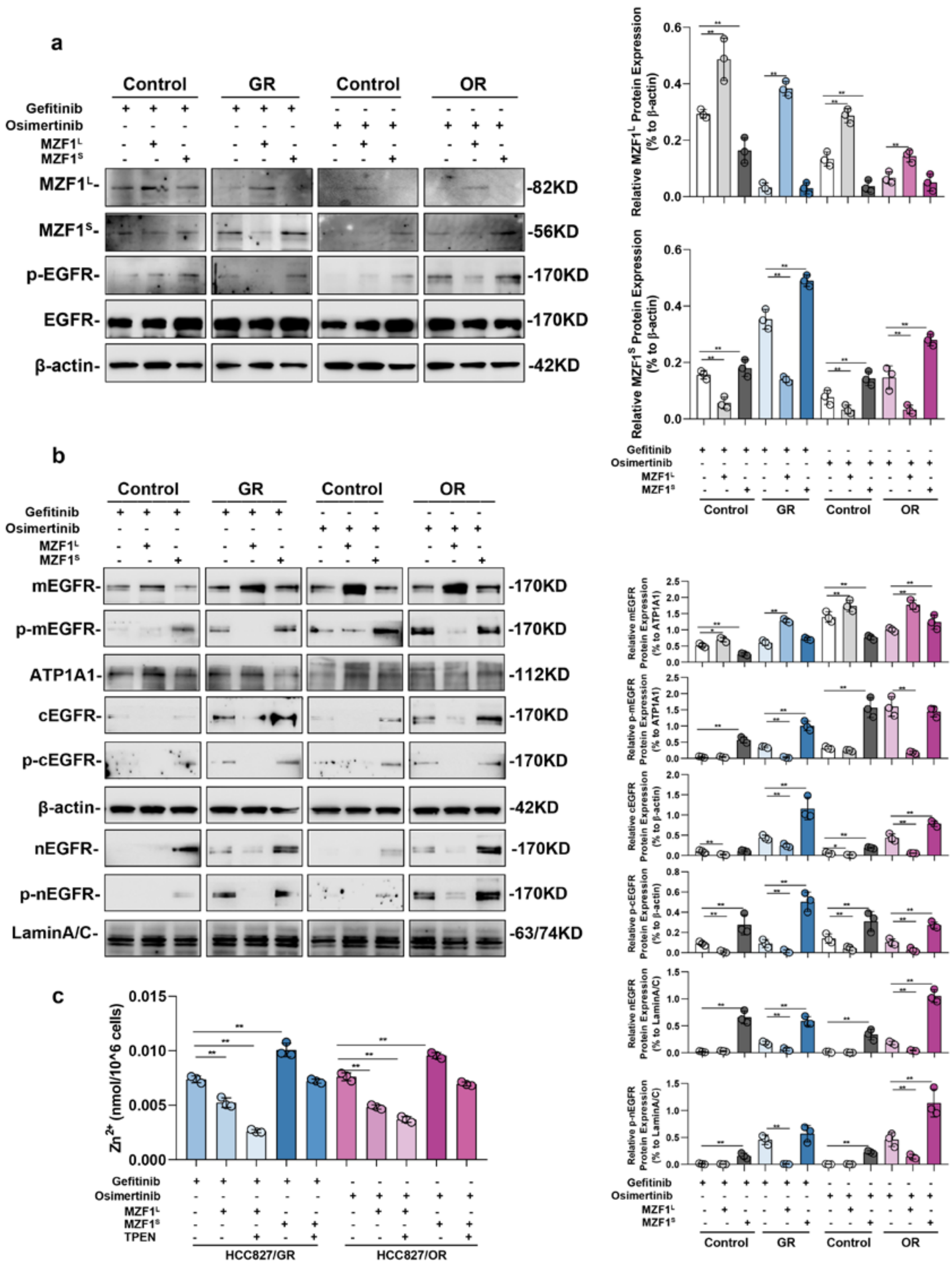

Supplementary Fig. 3 MZF1 splice variant overexpression differentially modulated EGFR and p-EGFR in EGFR-TKIs resistance cells. (a) Western blot and quantification of MZF1^{L}, MZF1^{S}, EGFR, and p-EGFR in GR/OR cells treated with gefitinib/osimertinib**. (b)** The protein level of EGFR and p-EGFR was shown in membrane extract (mEGFR/p-mEGFR), cytoplasmic extracts (cEGFR/p-cEGFR) and nuclear extracts (nEGFR/p-nEGFR). ATP1A1 served as membrane, β-actin as

cytoplasmic, and Lamin A/C as nuclear protein loading controls, respectively. **(c)** Intracellular Zn^{2+} analysis of EGFR and p-EGFR in HCC827, HCC827/GR and HCC827/OR were treated with gefitinib, osimertinib, TPEN and/or transfected with $MZF1^{L}$ and $MZF1^{S}$. *$P < 0.05$, **$P < 0.01$, significant differences from the control.

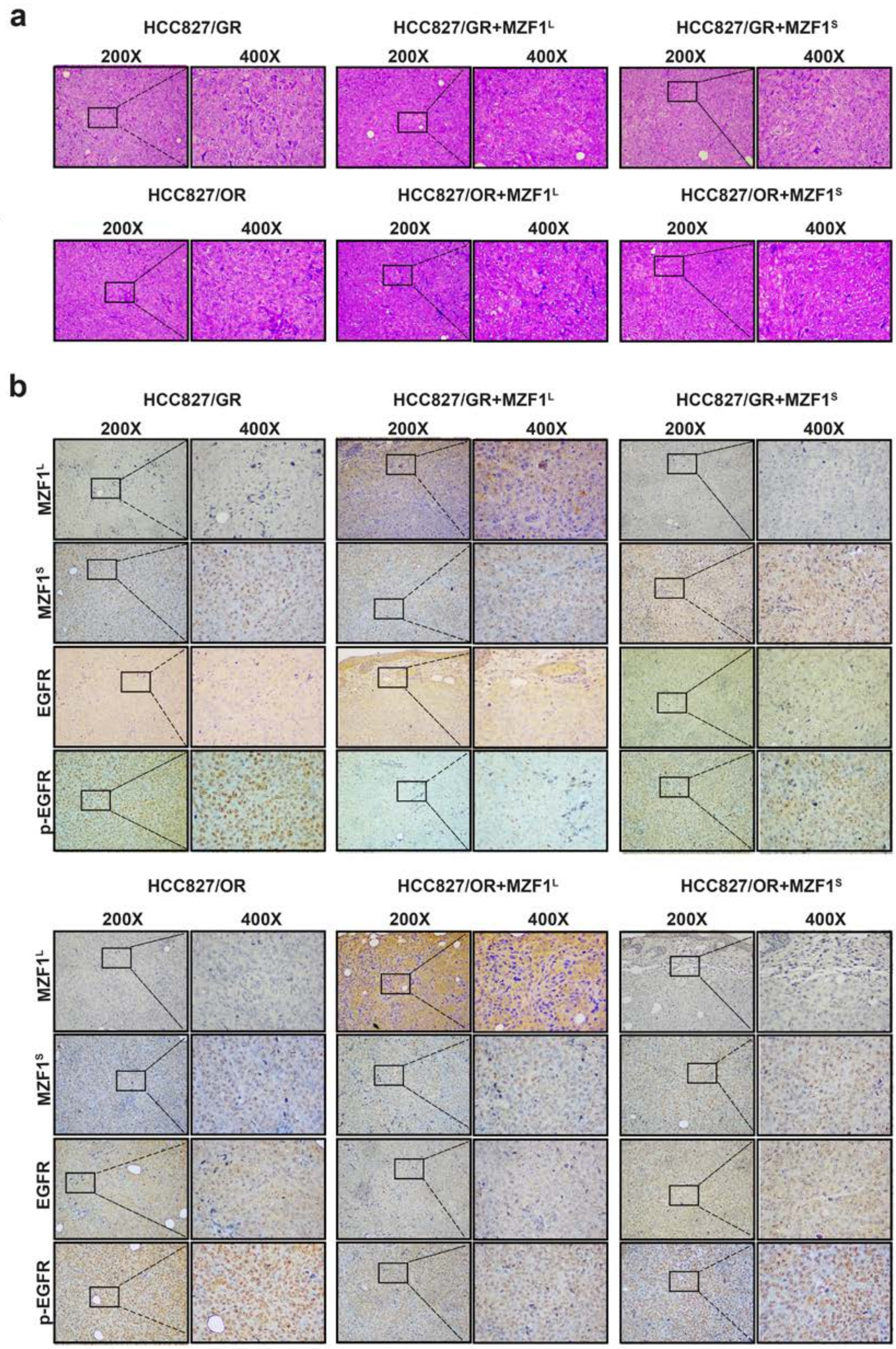

Supplementary Fig. 4 The effect of MZF1 isoforms on tumorigenicity in vivo. (a)-(b) H&E and Immunohistochemical staining of $MZF1^L$, $MZF1^S$, EGFR and p-EGFR in isolated xenograft tumors. Brown staining indicates the expression of protein. Each image is shown at a magnification of 200× and × 400.

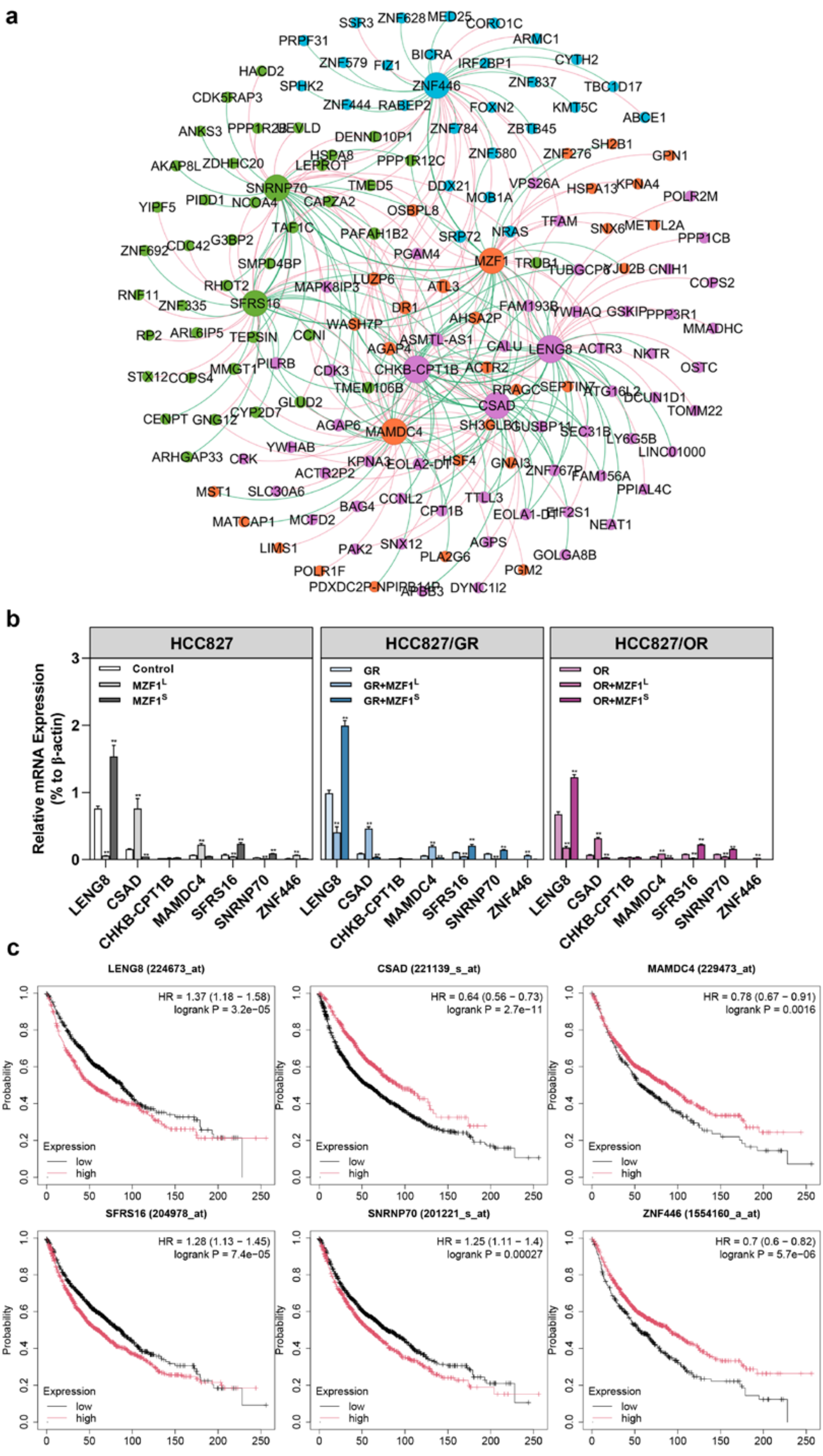
a
SSR3
ZNF628
MED25
CORO1C
PRPF31
ARMC1
BICRA
HACD2
ZNF579
FIZ1
IRF2BP1
CYTH2
SPHK2
ZNF446
ZNF837
TBC1D17
CDK5RAP3
ZNF444
RABEP2
FOXN2
KMT5C
ABCE1
ANKS3
DENND10P1
ZNF784
ZBTB45
SH2B1
HSPA8
PPP1R12C
ZNF580
ZNF276
GPN1
AKAP8L
ZDHHC20
LEPROT
SNRNP70
TMED5
DDX21
VPS26A
HSPA13
KPNA4
POLR2M
YIPF5
PIDD1
NCOA4
CAPZA2
MOB1A
OSBPL8
TFAM
SNX6
METTL2A
PPP1CB
TAF1C
PAFAH1B2
SRP72
NRAS
ZNF692
CDC42
G3BP2
PGAM4
MZF1
TRUB1
TUBGCP3
YJU2B
CNIH1
SMPD4BP
COPS2
RHOT2
LUZP6
ATL3
RNF11
MAPK8IP3
SFRS16
ZNF835
DR1
FAM193B
YWHAQ
GSKIP
PPP3R1
MMADHC
AHSA2P
RP2
ARL6IP5
TEPSIN
CCNI
WASH7P
ASMTL-AS1
CALU
LENG8
ACTR3
NKTR
AGAP4
OSTC
PILRB
CDK3
CHKB-CPT1B
ACTR2
STX12
COPS4
MMGT1
TMEM106B
RRAGC
SEPTIN7
ATG16L2
DCUN1D1
GLUD2
CSAD
TOMM22
CENPT
GNG12
CYP2D7
AGAP6
MAMDC4
SH3GLB1
GUSBP11
SEC31B
LY6G5B
YWHAB
LINC01000
ARHGAP33
CRK
KPNA3
EOLA2-DT
HSF4
GNAI3
ZNF767P
FAM156A
ACTR2P2
MST1
SLC30A6
PPIAL4C
BAG4
CCNL2
TTLL3
CPT1B
EOLA1-DT
EIF2S1
NEAT1
MATCAP1
MCFD2
LIMS1
PAK2
SNX12
AGPS
PLA2G6
GOLGA8B
POLR1F
PGM2
PDXDC2P-NPIPB14P
APBB3
DYNC1I2
b
HCC827
Control
MZF1^L
MZF1^S
HCC827/GR
GR
GR+MZF1^L
GR+MZF1^S
HCC827/OR
OR
OR+MZF1^L
OR+MZF1^S
Relative mRNA Expression (% to β-actin)
0
1
2
3
LENG8
CSAD
CHKB-CPT1B
MAMDC4
SFRS16
SNRNP70
ZNF446
c
LENG8 (224673_at)
HR = 1.37 (1.18 − 1.58)
logrank P = 3.2e−05
CSAD (221139_s_at)
HR = 0.64 (0.56 − 0.73)
logrank P = 2.7e−11
MAMDC4 (229473_at)
HR = 0.78 (0.67 − 0.91)
logrank P = 0.0016
SFRS16 (204978_at)
HR = 1.28 (1.13 − 1.45)
logrank P = 7.4e−05
SNRNP70 (201221_s_at)
HR = 1.25 (1.11 − 1.4)
logrank P = 0.00027
ZNF446 (1554160_a_at)
HR = 0.7 (0.6 − 0.82)
logrank P = 5.7e−06
Probability
Expression
low
high
0.0
0.2
0.4
0.6
0.8
1.0
0
50
100
150
200
250

Supplementary Fig. 5 Downstream target genes of the MZF1 isoforms. (a) Directed network analysis of MZF1 downstream target genes. **(b)** RT-qPCR analysis of MZF1-targeted genes in $MZF1^L$/$MZF1^S$ overexpression cell. **(c)** Prognostic prediction value of LENG8, CSAD, MAMDC4, SFRS16, SNRP70, ZNF446 expression in lung cancer patients. Data were obtained from Kaplan-Meier plotter (https://kmplot.com/). $^*P < 0.05$, $^{**}P < 0.01$, significant differences from the control.

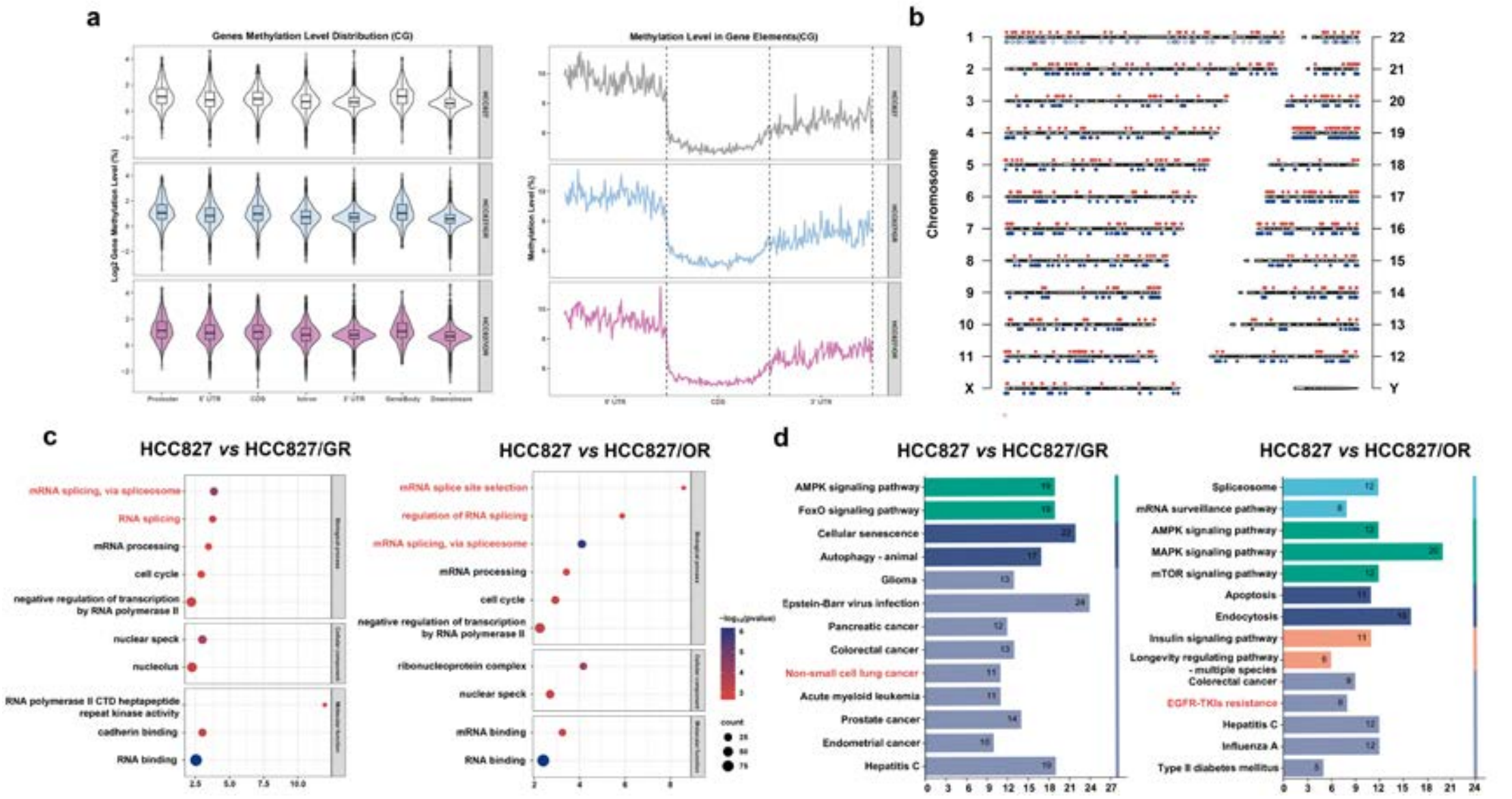

Supplementary Fig. 6 RNA Bis-seq in EGFR-TKIs resistant cells. (a) RNA m^{5}C modification status across 5'-UTR, CDS, and 3'-UTR regions in resistant cells. **(b)** Chromosomal distribution map of DMRs between sensitive and resistant cell groups. **(c)- (d)** Functional enrichment analysis of DMR-related genes, Gene Ontology (GO) (c) and KEGG pathway (d).

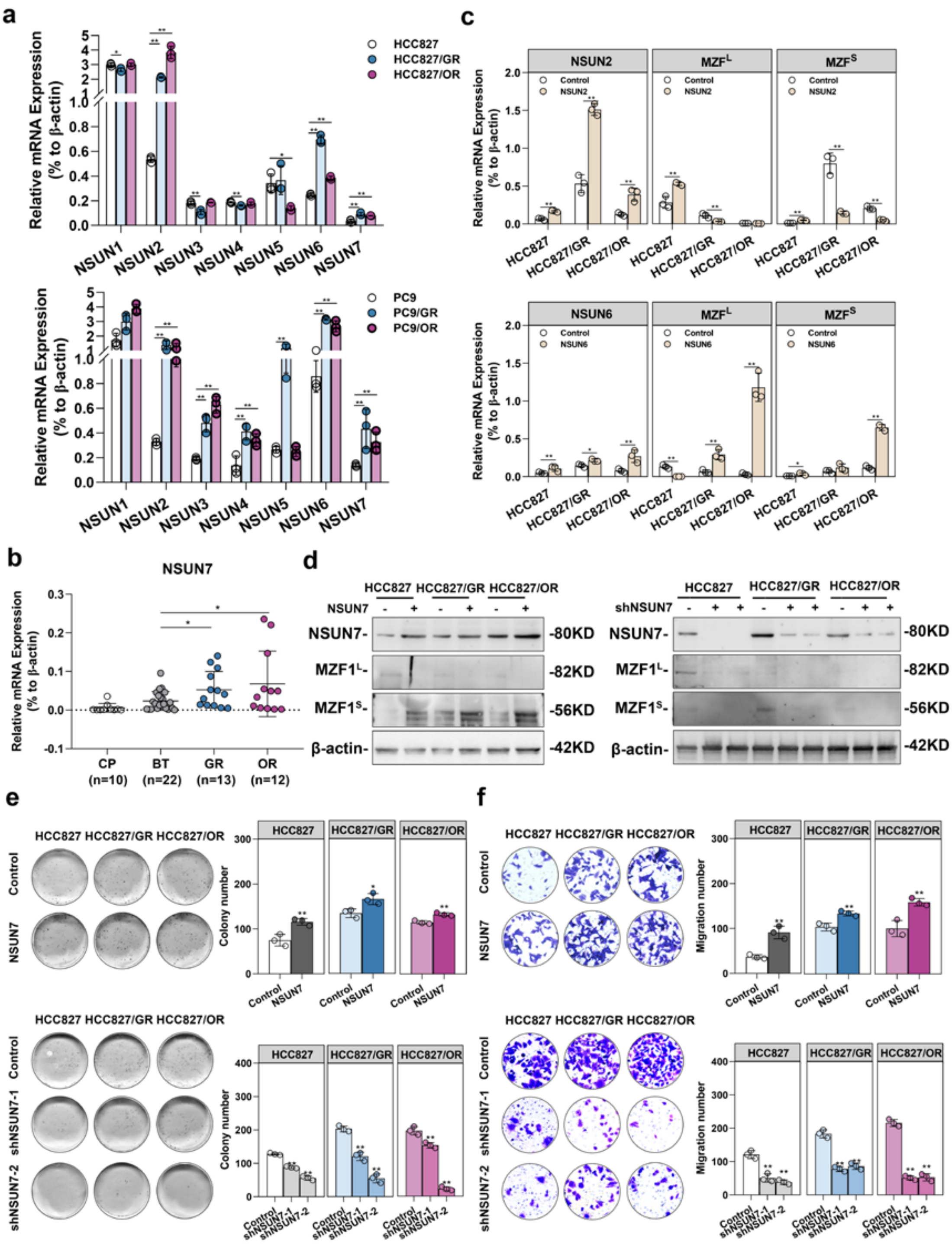

Supplementary Fig. 7 Expression and function of NSUNs in HCC827, HCC827/GR and HCC827/OR. (a) RT-qPCR of NSUNs expression in HCC827, HCC827/GR and HCC827/OR cells. **(b)** RT–qPCR of NSUN7 expressed in BALF from Cohort1 patients with CP (chronic pneumonia, n=10), BT (patient before treatment, n=22), GR (gefitinib resistant patient, n=13) and OR (osimertinib resistant patient, n=12). **(c)** RT-qPCR analysis of MZF1^{L} and MZF1^{S} expression following

NSUN2 or NSUN6 overexpression in HCC827, HCC827/GR and HCC827/OR cells. **(d)** Western blots analysis of the expression of NSUN7, MZF1^L and MZF1^S in NSUN7 overexpressed/knockdown GR and OR cells. **(e)-(f)** Colony formation (e) and Transwell assays (f) evaluated proliferative and migratory capacities stably transfected with NSUN7or NSUN7 knockdown. $*P < 0.05$, $**P < 0.01$, significant differences from the control.

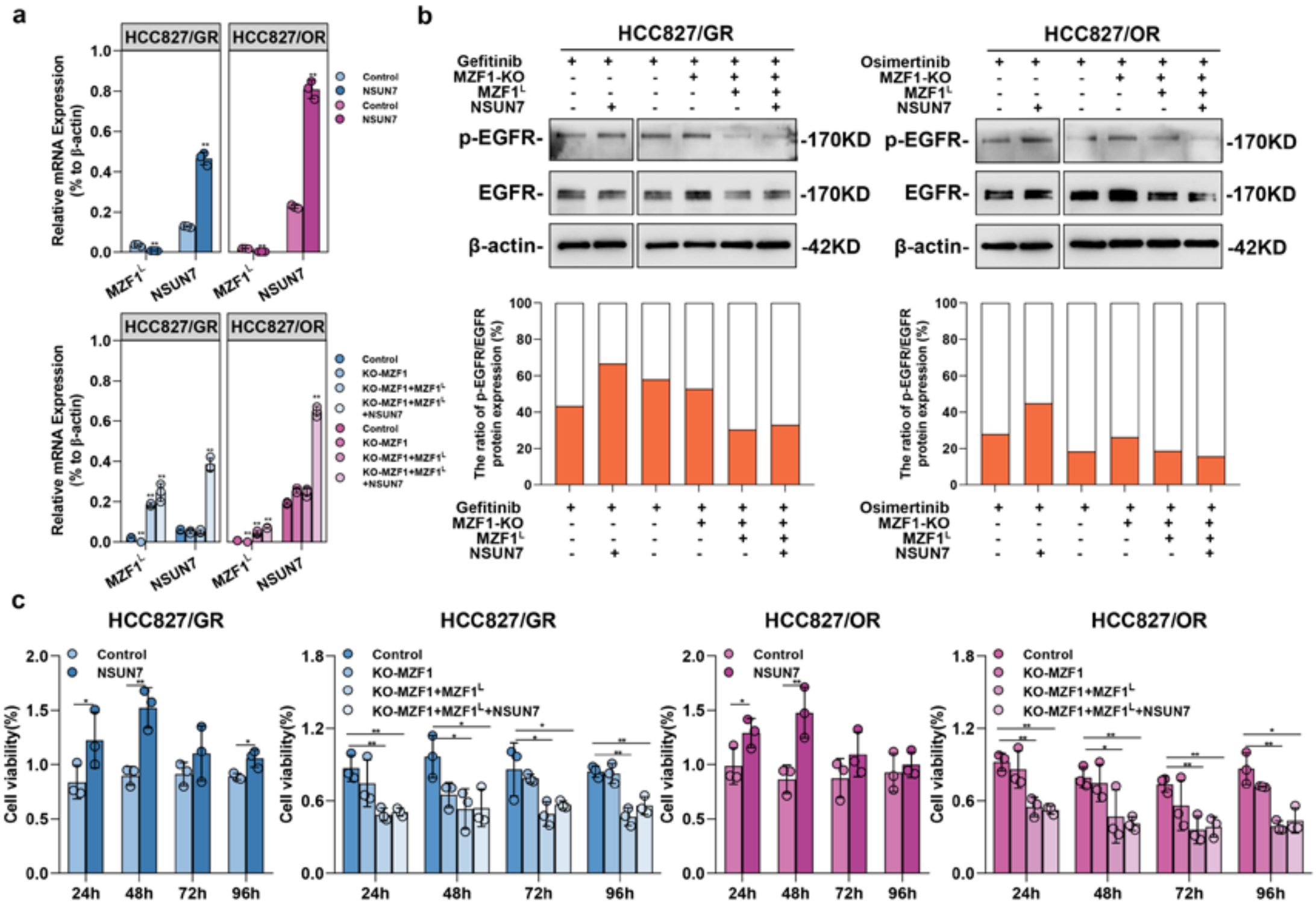

Supplementary Fig. 8 The expression of EGFR and p-EGFR in MZF1^{L} and/ or NSUN7 overexpressed HCC827/GR and HCC827/OR cells. (a) RT-qPCR analysis of MZF1^{L} and NSUN7 mRNA expression in HCC827/GR and HCC827/OR cells treated with EGFR-TKIs and rescued with MZF1^{L} and/or NSUN7. **(b)** Western blot and quantification of p-EGFR/EGFR in HCC827/GR and HCC827/OR cells treated with EGFR-TKIs and rescued with MZF1^{L} and/or NSUN7. **(c)** Time-course cell viability assays of indicated cell groups (24-96h), with values normalized to untreated HCC827/GR and HCC827/OR control cells. *P < 0.05, **P < 0.01, significant differences from the control.

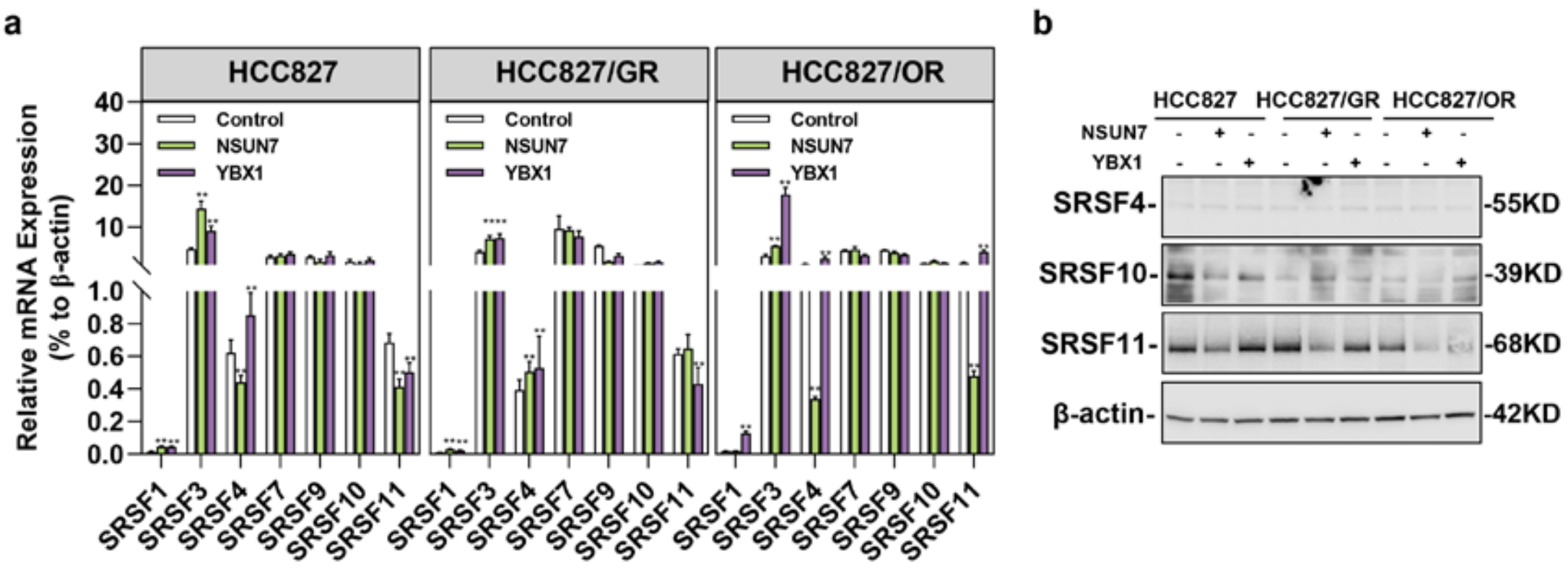

Supplementary Fig. 9 Expression of SRSFs in HCC827, HCC827/GR and HCC827/OR cells. (a) RT-qPCR of SRSF1, SRSF3, SRSF4, SRSF7, SRSF9, SRSF10 and SRSF11 expression following NSUN7 or YBX1 overexpression in HCC827, HCC827/GR and HCC827/OR cells. **(b)** Western blot analysis of SRSF4, SRSF10 and SRSF11 expression following NSUN7 or YBX1 overexpression in HCC827, HCC827/GR and HCC827/OR cells. *$P < 0.05$, **$P < 0.01$, significant differences from the control.

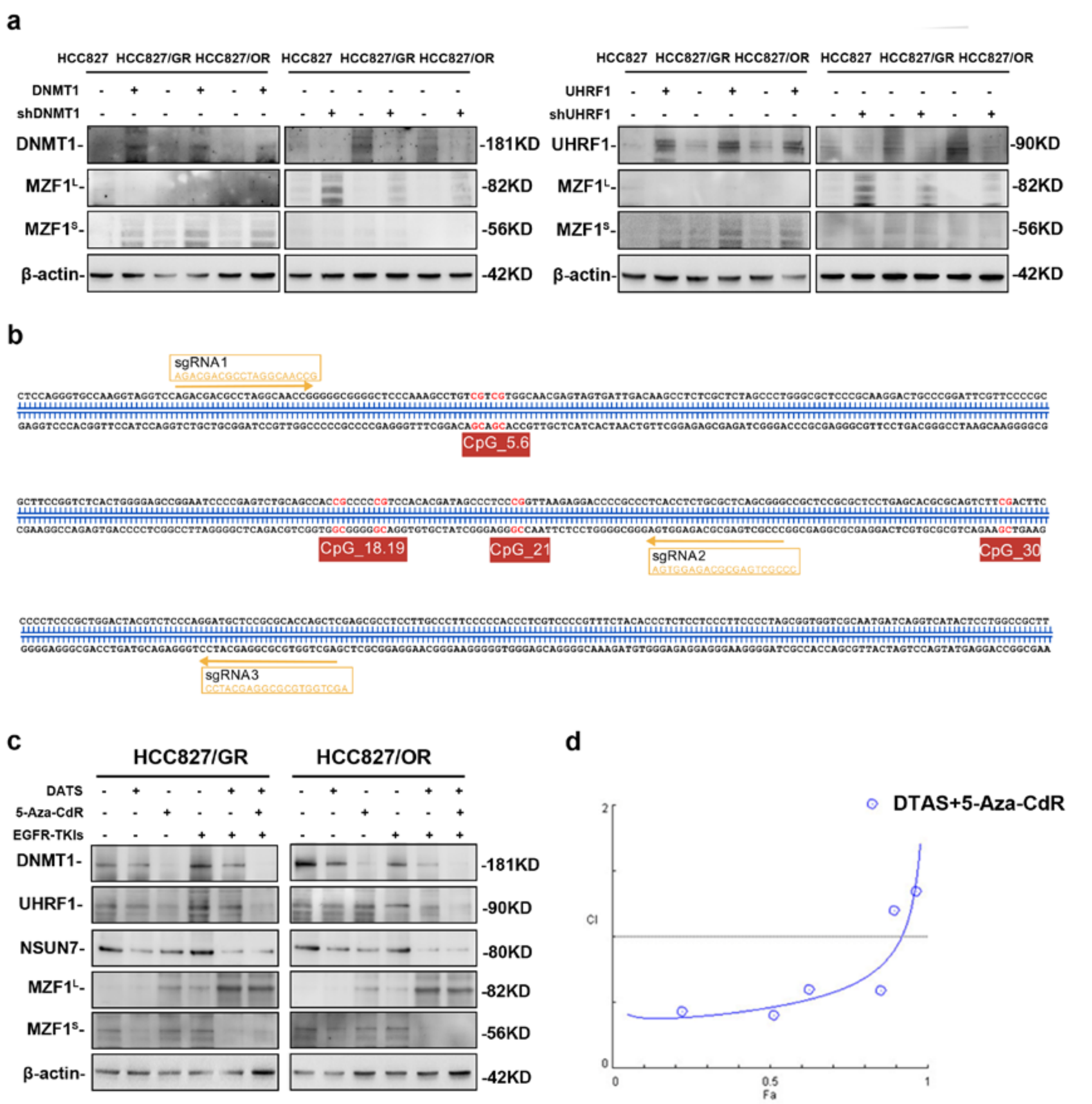

Supplementary Fig. 10 DNA 5-mC regulated MZF1 alternative splicing. (a) Western blot analyzed the effects of UHRF1/DNMT1 overexpression or knockdown on MZF1 splicing variants. **(b)** Schematic diagram of targeted methylation site-specific modification at the MZF1 promoter using the dCas9-Tet1-CD system guided by sgRNA. **(c)** Western blot detected DNMT1, UHRF1, NSUN7, and MZF1 splice variants after DATS, 5-Aza-CdR, and/or EGFR-TKIs treatment. **(d)** Combination index plot for DATS and 5-Aza-CdR combination therapy.

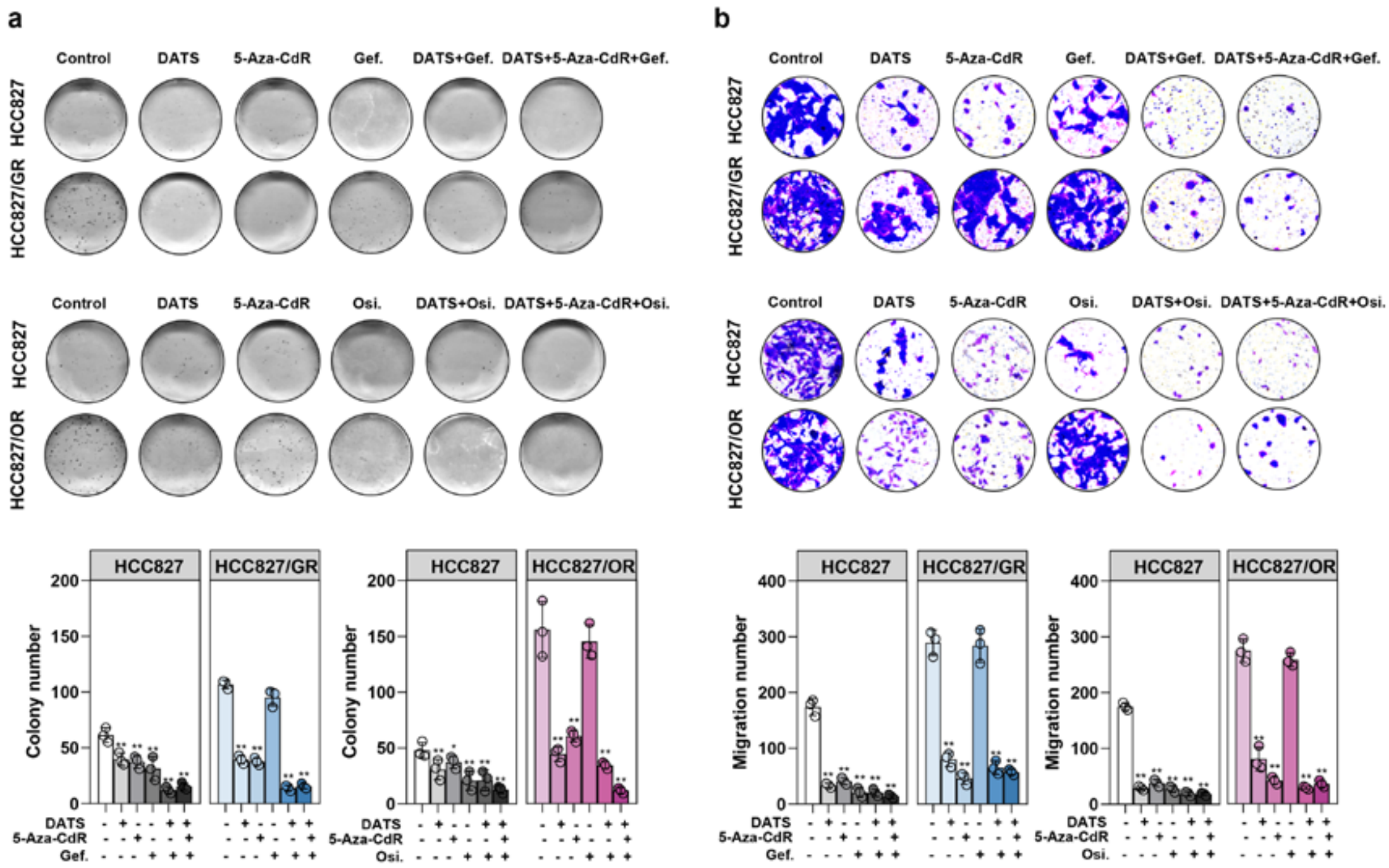

Supplementary Fig. 11 DATS combined with 5-Aza-CdR suppressed EGFR-TKIs resistance. (a)-(b) Colony formation (a) and Transwell (b) assays evaluated proliferative and migratory capacities after DATS, 5-Aza-CdR, and/or EGFR-TKIs treatment. **P* < 0.05, ***P* < 0.01, significant differences from the control.

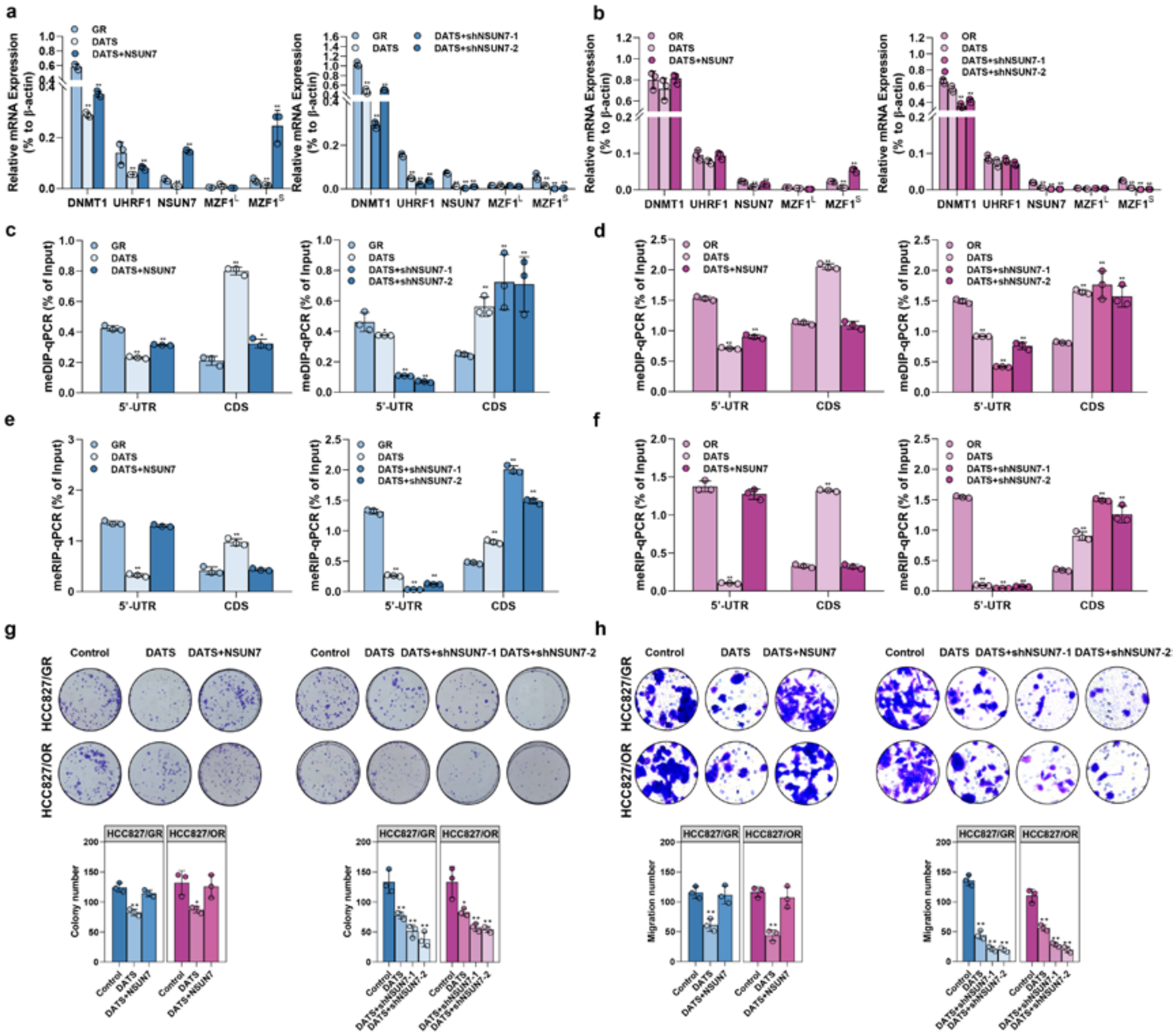

Supplementary Fig. 12 DATS inhibited EGFR-TKIs resistance by suppressing NSUN7 expression. (a)-(b) RT-qPCR detected DNMT1, UHRF1, NSUN7, and MZF1 splice variants after DATS, and/or NSUN7/shNSUN7 treatment in HCC827/GR (a) and HCC827/OR(b). **(c)-(d)** MeDIP-qPCR analysis was showed 5-mC enrichment at MZF1 5'-UTR and CDS regions after DATS, and/or NSUN7/shNSUN7 treatment in HCC827/GR (c) and HCC827/OR(d). **(e)-(f)** MeRIP-qPCR analysis was showed 5-mC enrichment at MZF1 5'-UTR and CDS regions after DATS, and/or NSUN7/shNSUN7 treatment in HCC827/GR (e) and HCC827/OR(f). **(g)-(h)** Colony formation (g) and Transwell (h) assays evaluated proliferative and migratory capacities after DATS, and/or NSUN7/shNSUN7 treatment in HCC827/GR and HCC827/OR. **P* < 0.05, ***P* < 0.01, significant differences from the control.

Supplementary Table 1. Oligonucleotides

Target gene	Primer ID	Sequence (5' to 3')	Oligo use for
MZF1^{L}	F	GGGGGCATCTTCTCCCCA	RT–qPCR
	R	CACCTTGCCACATACATCGC	
MZF1^{S}	F	CCGTGCTGGACCAGATCTTT	
	R	GGCCCCTGGGGAGAAGA	
NSUN1 (NOP2)	F	CCCTCAGTCCCAGACAGGAAA	
	R	CTGAGTCTGCCCCTTTGGAG	
NUSN2	F	GAACTTGCCTGGCACACAAAT	
	R	TGCTAACAGCTTCTTGACGACTA	
NSUN3	F	TGGGTCTGTTTGGAATCCTATT	
	R	TGCACCACCTTAAATCATTGTTAC	
NSUN4	F	CCATCAATCCGTGTCAGTCTC	
	R	GCTTAGCACTTACATGATCCCAG	
NSUN5	F	CGCTACCATGAGGTCCACTAC	
	R	GCATCTCGCACCACGTCTT	
NSUN6	F	CAGAATGCCTTATTGTTAGGGCT	
	R	ACCATATCAAGTTTAACCGCCTT	
NSUN7	F	CGCCCTCTCGATTTACCATA	
	R	GCAACTGTGTACCACGAACC	
ALYREF	F	GCAGGCCAAAACAACTTCCC	
	R	AGTTCCTGAATATCGGCGTCT	
YBX1	F	AAGGAGAAAAGGGTGCGGAG	
	R	CCTACGACGTGGATAGCGTC	
SRSF1	F	AATATGAATAGATTCCCCGTA	
	R	ATCATATTAAACGCAAGCA	
SRSF3	F	GGCAATCTTGGAAACAATGG	
	R	TTCACCATTCGACAGTTCCA	
SRSF4	F	AAAAGTCGGAGCAGGAGTCA	
	R	CTTCTCTTCCTGCCCTTCCT	
SRSF7	F	AAGAAGCAGCCGATCAAAG	
	R	AAAGGGTGAACTTGAGAGC	
SRSF9	F	CATGGAATATGCCCTGCGTAAAC	
	R	ACCGTGAGTAGCCATAGCTGGTG	
SRSF10	F	TGAGGATGTTCGTGATGCTGA	
	R	CCTCCTTTCATAACTTCGGCTT	
SRSF11	F	GACTAATGTCTCCCCGAGCG	
	R	AGACTGGCAAAGGCGAATCA	
β-actin	F	TTAGTTGCGTTACACCCTTTC	
	R	ACCTTCACCGTTCCAGTTT	
MZF1 sgRNA-1		AGACGACGCCTAGGCAACCG	sgRNA

MZF1 sgRNA-2		CCCGCTGAGCGCAGAGGTGA	
MZF1 sgRNA-3		AGCTGGTGCGCGGAGCATCC	
NSUN7-1		GGGCTATCCGGACTCCGTTTA	shRNA
NSUN7-2		GGACAAATCTCGAAGTCTTGC	
DNMT1		GACGACCCTGACCTCAAATAT	
UHRF1		CCGCACCAAGGAATGTACCAT	
IP-5’UTR	F	CCTGACCCTCTGATCCACTC	MeRIP-qPCR RIP-qPCR
	R	ACGGAGAGAGACCAGAGACT	
IP-CDS	F	GCACCAGAAGATCCACACG	
	R	ACTCTCCGATGCTCTTCCAG	
Positive control	F	GGCGTAATGGAATGCTTGA	
	R	CCTCGCCTAGTCTGGAAGC	
Negative control	F	GGTTCAGTTTATTGTCCTAAAATCAG	
	R	TCAGCCAGACCAAAGCAAAT	
MZF1-M	F	TTTATAGAAAAATTAAGAAATTAGTCGG	MS-qPCR
	R	AAATACAATAACGAAATCATACGTC	
MZF1-U	F	TTTATAGAAAAATTAAGAAATTAGTTGG	
	R	AAATACAATAACAAAATCATACATC	
MZF1	F	aggaagagag TTTGTGAGTTTGTTTTTTTGTTTTT	MassARRAY
	R	cagtaatacgactcactatagggagaaggct AATTAACAAAATCCTACACCAATCA	

Supplementary Table 2. Antibodies used in this study.

Antibody	Type	Vendor	Cat#	WB	IF	IHC	ChIP or MeDI
Anti-MZF1^L	rabbit polyclonal	Customer order		1:10000	1:800	1:1000	NA
Anti-MZF1^S	rabbit polyclonal			1:10000	1:800	1:1000	NA
Anti-EGFR	mouse monoclonal	Proteintech	66455-1-Ig	1:3000	NA	1:500	NA
Anti-phospho-EGFR	rabbit polyclonal	Proteintech	30277-1-AP	1:1000	NA	1:100	NA
Anti-ATP1A1	mouse monoclonal	Proteintech	55187-1-AP	1:5000	NA	NA	NA
Anti-Lamin A/C	rabbit polyclonal	Proteintech	10298-1-AP	1:6000	NA	NA	NA
Anti-β-actin	mouse monoclonal	Sigma Aldrich	A4551	1:10000	NA	NA	NA
Anti-5-methylcytidine (5mC)	rabbit monoclonal	Active Motif	91311	NA	NA	NA	0.5 μg
Anti-ALYREF	rabbit monoclonal	Proteintech	16690-1-AP	1:800	NA	NA	NA
Anti-YBX1	rabbit monoclonal	Proteintech	20339-1-AP	1:5000	NA	NA	4 μg
Anti-NSUN7	rabbit monoclonal	Proteintech	17546-1-AP	1:800	NA	NA	4 μg
Anti-SRSF1	rabbit monoclonal	Abcam	ab133689	1:1000	NA	NA	4 μg
Anti-SRSF3	rabbit monoclonal	Abcam	ab198291	1:1000	NA	NA	4 μg
Anti-SRSF4	rabbit monoclonal	Abcam	ab235606	1:800	NA	NA	NA
Anti-SRSF10	rabbit monoclonal	Thermo Fisher	PA5-30170	1:800	NA	NA	NA
Anti-SRSF11	rabbit polyclonal	Thermo Fisher	PA5-52606	1:800	NA	NA	NA
Anti-DNMT1	mouse monoclonal	Abcam	ab188453	1:1000	NA	1:200	2 μg
Anti-UHRF1	rabbit monoclonal	Active Motif	61341	1:2000	NA	1:200	2 μg
Goat Anti-Mouse IgG H+L (HRP)		Abcam	ab6789	1:2500	NA	NA	NA
Goat Anti-Rabbit IgG H+L (HRP)		Abcam	ab6721	1:2500	NA	NA	NA

Supplementary Table 3. Reagents used in this study.

**Reagents**	**Vendor**	**Cat#**	**Concentration (*in vitro*)**	**Concentration (*in vivo*)**
Gefitinib	Sigma Aldrich	SML1657	1 μM	50mg/kg
Osimertinib	MCE	HY-15572	0.1 μM	10mg/kg
5-aza-2'-deoxycytidine	Sigma Aldrich	189825	5 μM	0.5mg/kg
DATS	MCE	HY-117235	25 μM	40mg/kg
TPEN	MCE	HY-100202	2.5μM	NA

Supplementary Table 4. The association of MZF1 transcripts expression with clinicpathologic parameters in NSCLC (BALF, n=57)

Characteristics	Cases	MZF1^L expression			MZF1^S expression		
		High (%)	Low (%)	*P*-value	High (%)	Low (%)	*P*-value
**Total**	57	20(35.1)	37(64.9)		18(31.6)	39(68.4)	
**Gender**							
Male	26	12(46.2)	14(53.8)		9(34.6)	17(65.4)	
Female	31	8(25.8)	23(74.2)	0.1089	9(29.0)	22(71.0)	0.6515
**Age at diagnosis**							
≥60	39	12(30.8)	27(69.2)		12(30.8)	27(69.2)	
<60	18	8(44.4)	10(55.6)	0.3146	6(33.3)	12(66.7)	0.8465
**Pathological type**							
Pneumonia	10	9(90.0)	1(10.0)		2(20.0)	8(80.0)	
Adenocarcinoma	43	10(23.3)	33(76.7)	**0.0003**	15(34.9)	28(65.1)	0.3637
Squamous cell carcinomas	4	1(25.0)	3(75.0)	**0.0150**	1(25.0)	3(75.0)	0.8368
**Tumor invasive depth**							
1	18	8(44.4)	10(55.6)		3(16.7)	15(83.3)	
2	14	2(14.3)	12(85.7)	**0.0679**	6(42.9)	8(57.1)	0.1021
3	8	0(0.0)	8(100.0)	**0.0234**	3(37.5)	5(62.5)	0.2446
4	7	1(14.3)	6(85.7)	0.1584	3(42.9)	4(57.1)	0.1686
NA	10						
**Lymph node metastasis**							
0	22	9(40.9)	13(59.1)		4(18.2)	18(81.8)	
1	2	0(0.0)	2(100.0)	0.2526	1(50.0)	1(50.0)	0.2888
2	12	2(16.7)	10(83.3)	0.1487	4(33.3)	8(66.7)	0.3196

3	11	0(0.0)	11(100.0)	**0.0129**	7(63.6)	4(36.4)	**0.0090**
NA	10						
**Distant metastasis**							
Yes	17	1(5.9)	16(94.1)		9(52.9)	8(47.1)	
No	30	10(33.3)	20(66.7)	**0.0327**	7(23.3)	23(76.7)	**0.0396**
NA	10						
**Clinic stage**							
I	18	7(38.9)	11(61.1)		3(16.7)	15(83.3)	
II	1	0(0.0)	1(100.0)	0.4326	1(100.0)	0(0.0)	**0.0466**
III	9	3(33.3)	6(66.7)	0.7781	3(33.3)	6(66.7)	0.3261
IV	19	1(5.3)	18(94.7)	**0.0130**	9(47.4)	10(52.6)	**0.0462**
NA	10						
**EGFR resistance**							
No	22	10(45.5)	12(54.5)		2(9.1)	20(90.9)	
GR	13	1(76.9)	12(23.1)	**0.0201**	8(61.5)	5(38.5)	**0.0009**
OR	12	0(0.0)	12(100.0)	**0.0054**	6(50.0)	6(50.0)	**0.0072**
NA	10						

Supplementary Table 5. Summary of patients' clinical information (BALF, n=57).

Sample ID	Sex	Age	Pathological type	T	N	M	Clinical stage
CP001	Male	70	pneumonia				
CP002	Female	63	pneumonia				
CP003	Male	38	pneumonia				
CP004	Female	72	pneumonia				
CP005	Male	86	pneumonia				
CP006	Male	69	pneumonia				
CP007	Female	50	pneumonia				
CP008	Male	75	pneumonia				
CP009	Female	55	pneumonia				
CP010	Female	54	pneumonia				
BT001	Female	65	Lung adenocarcinoma	1	0	0	I
BT002	Male	66	Lung adenocarcinoma	4	2	0	III
BT003	Female	75	Lung adenocarcinoma	1	0	1	IV
BT004	Male	75	Lung adenocarcinoma	1	0	0	I
BT005	Male	68	Lung adenocarcinoma	1	0	0	I
BT006	Male	62	Lung squamous cell carcinomas	2	2	0	III
BT007	Female	63	Lung adenocarcinoma	1	0	0	I
BT008	Male	62	Lung adenocarcinoma	1	0	0	I
BT009	Male	63	Lung adenocarcinoma	1	0	0	I
BT010	Male	57	Lung adenocarcinoma	1	0	0	I
BT011	Female	68	Lung adenocarcinoma	1	0	0	I
BT012	Female	65	Lung adenocarcinoma	1	0	0	I
BT013	Female	62	Lung adenocarcinoma	1	0	0	I
BT014	Male	63	Lung adenocarcinoma	2	2	0	I
BT015	Female	58	Lung adenocarcinoma	1	0	0	III
BT016	Male	57	Lung adenocarcinoma	1	0	0	I
BT017	Male	40	Lung adenocarcinoma	1	0	0	I
BT018	Male	65	Lung adenocarcinoma	1	0	0	I
BT019	Male	76	Lung squamous cell carcinomas	1	0	0	I
BT020	Male	73	Lung squamous cell carcinomas	2	0	0	I
BT021	Female	31	Lung adenocarcinoma	1	0	0	I
BT022	Female	55	Lung adenocarcinoma	2	0	1	IV
GR001	Female	70	Lung adenocarcinoma	3	3	0	IV
GR002	Male	66	Lung adenocarcinoma	2	2	0	III
GR003	Female	45	Lung adenocarcinoma	3	3	1	IV
GR004	Female	76	Lung adenocarcinoma	3	2	0	III
GR005	Female	37	Lung adenocarcinoma	3	2	1	IV
GR006	Female	57	Lung adenocarcinoma	2	3	1	IV
GR007	Female	69	Lung adenocarcinoma	4	3	1	IV
GR008	Male	63	Lung squamous cell carcinomas	2	1	0	II

GR009	Male	65	Lung adenocarcinoma	1	0	0	I
GR010	Female	62	Lung adenocarcinoma	4	3	1	IV
GR011	Male	64	Lung adenocarcinoma	4	2	1	IV
GR012	Female	58	Lung adenocarcinoma	2	2	0	III
GR013	Male	54	Lung adenocarcinoma	3	0	1	IV
OR001	Male	64	Lung adenocarcinoma	4	3	1	IV
OR002	Female	63	Lung adenocarcinoma	4	0	1	IV
OR003	Female	54	Lung adenocarcinoma	3	3	0	III
OR004	Female	70	Lung adenocarcinoma	2	3	1	IV
OR005	Female	61	Lung adenocarcinoma	2	2	1	IV
OR006	Female	66	Lung adenocarcinoma	2	2	0	III
OR007	Female	62	Lung adenocarcinoma	3	3	0	IV
OR008	Male	58	Lung adenocarcinoma	2	2	0	III
OR009	Female	67	Lung adenocarcinoma	3	3	1	IV
OR010	Male	51	Lung adenocarcinoma	2	1	1	IV
OR011	Female	61	Lung adenocarcinoma	2	2	1	IV
OR012	Female	66	Lung adenocarcinoma	4	3	1	IV

Supplementary Table 6. Summary of patients' clinical information (Tissue, n=179)

Sample ID	Sex	Age	Pathological type	T	N	M	Clinical stage
CP001	Male	70	pneumonia				
CP002	Female	63	pneumonia				
CP003	Male	41	pneumonia				
CP004	Male	86	pneumonia				
CP005	Male	69	Bronchial polyp				
CP006	Male	75	pneumonia				
CP007	Male	47	pneumonia				
CP008	Male	65	pneumonia				
CP009	Male	64	pneumonia				
CP010	Female	55	pneumonia				
CP011	Female	59	Paracancerous				
CP012	Male	59	Paracancerous				
CP013	Female	50	Paracancerous				
CP014	Male	60	Paracancerous				
CP015	Male	36	Paracancerous				
CP016	Male	49	Paracancerous				
CP017	Male	77	Paracancerous				
CP018	Male	59	Paracancerous				
CP019	Male	41	Paracancerous				
CP020	Male	58	Paracancerous				
CP021	Female	52	Paracancerous				
CP022	Female	66	Paracancerous				
CP023	Female	63	Paracancerous				
CP024	Female	54	Paracancerous				
CP025	Male	68	Paracancerous				
CP026	Male	52	Paracancerous				
CP027	Female	65	Paracancerous				
CP028	Female	65	Paracancerous				
CP029	Male	73	Paracancerous				
CP030	Female	52	Paracancerous				
CP031	Female	45	Paracancerous				
CP032	Male	61	Paracancerous				
CP033	Male	32	Paracancerous				
CP034	Male	70	Paracancerous				
CP035	Female	73	Paracancerous				
CP036	Female	68	Paracancerous				
CP037	Male	58	Paracancerous				
CP038	Male	63	Paracancerous				
CP039	Female	60	Paracancerous				
CP040	Male	69	pneumonia				

CP041	Male	44	pneumonia				
CP042	Male	66	Paracancerous				
CP043	Female	76	Paracancerous				
CP044	Female	53	Paracancerous				
CP045	Female	76	Paracancerous				
CP046	Male	60	Paracancerous				
CP047	Female	65	Paracancerous				
CP048	Male	62	Paracancerous				
CP049	Female	63	Paracancerous				
CP050	Female	53	Paracancerous				
CP051	Female	37	Paracancerous				
CP052	Male	62	Paracancerous				
CP053	Male	63	Paracancerous				
CP054	Male	57	Paracancerous				
CP055	Male	45	Paracancerous				
CP056	Male	69	Paracancerous				
CP057	Female	76	Paracancerous				
CP058	Female	67	Paracancerous				
CP059	Male	68	Paracancerous				
CP060	Female	80	Paracancerous				
CP061	Female	73	Paracancerous				
BT001	Male	62	Lung squamous cell carcinomas	2	2	0	III
BT002	Female	63	Lung adenocarcinoma	1	0	0	I
BT003	Female	53	Lung adenocarcinoma	1	0	0	I
BT004	Female	37	Lung adenocarcinoma	1	0	0	I
BT005	Male	62	Lung adenocarcinoma	1	0	0	I
BT006	Male	63	Lung adenocarcinoma	1	0	0	I
BT007	Male	57	Lung adenocarcinoma	1	0	0	I
BT008	Male	45	Lung adenocarcinoma	1	0	0	I
BT009	Female	68	Lung adenocarcinoma	1	0	0	I
BT010	Male	62	Lung squamous cell carcinomas	4	3	1	IV
BT011	Female	65	Lung adenocarcinoma	1	0	0	I
BT012	Female	62	Lung adenocarcinoma	1	0	0	I
BT013	Male	63	Lung adenocarcinoma	2	0	0	I
BT014	Male	68	Lung squamous cell carcinomas	1	0	0	I
BT015	Male	68	Lung squamous cell carcinomas	NA	NA	NA	NA
BT016	Female	58	Lung adenocarcinoma	2	2	0	III
BT017	Male	57	Lung adenocarcinoma	1	0	0	I
BT018	Male	62	Lung squamous cell carcinomas	2	2	0	III
BT019	Male	82	Lung squamous cell carcinomas	4	2	1	IV
BT020	Female	55	Lung adenocarcinoma	1	0	0	I
BT021	Male	40	Lung adenocarcinoma	1	0	0	I
BT022	Female	59	Lung adenocarcinoma	1	0	0	I
BT023	Male	65	Lung adenocarcinoma	1	0	0	I

BT024	Male	76	Lung squamous cell carcinomas	1	0	0	I
BT025	Male	73	Lung squamous cell carcinomas	2	0	0	I
BT026	Male	65	Lung squamous cell carcinomas	4	3	1	IV
BT027	Female	31	Lung adenocarcinoma	1	0	0	I
BT028	Female	53	Lung adenocarcinoma	2	0	0	II
BT029	Female	76	Lung adenocarcinoma	2	2	0	III
BT030	Male	60	Lung adenocarcinoma	1	0	0	I
BT031	Female	76	Lung adenocarcinoma	2	2	0	III
BT032	Female	65	Lung adenocarcinoma	1	0	0	I
BT033	Male	66	Lung squamous cell carcinomas	3	0	0	III
BT034	Male	69	Lung squamous cell carcinomas	3	0	0	III
BT035	Male	67	Lung adenocarcinoma	4	2	1	IV
BT036	Female	54	Lung adenocarcinoma	1	2	0	III
BT037	Female	55	Lung adenocarcinoma	3	3	1	III
BT038	Male	54	Lung squamous cell carcinomas	4	3	1	IV
BT039	Male	69	Lung squamous cell carcinomas	4	2	1	IV
BT040	Male	62	Lung squamous cell carcinomas	4	3	1	IV
BT041	Male	66	Lung squamous cell carcinomas	4	3	1	IV
BT042	Female	52	Lung adenocarcinoma	1	0	0	I
BT043	Female	66	Lung adenocarcinoma	1	0	0	I
BT044	Female	63	Lung adenocarcinoma	1	0	0	I
BT045	Female	54	Lung adenocarcinoma	1	0	0	I
BT046	Male	68	Lung squamous cell carcinomas	1	0	0	I
BT047	Male	52	Lung adenocarcinoma	1	0	0	I
GR001	Female	70	Lung adenocarcinoma	3	3	0	IV
GR002	Male	66	Lung adenocarcinoma	2	2	0	III
GR003	Female	62	Lung adenocarcinoma	4	3	1	IV
GR004	Female	63	Lung adenocarcinoma	4	2	1	IV
GR005	Female	56	Lung adenocarcinoma	4	2	0	III
GR006	Female	54	Lung adenocarcinoma	3	3	0	III
GR007	Female	45	Lung adenocarcinoma	3	3	1	IV
GR008	Female	70	Lung adenocarcinoma	2	3	1	IV
GR009	Female	37	Lung adenocarcinoma	3	2	1	IV
GR010	Female	61	Lung adenocarcinoma	2	2	1	IV
GR011	Female	57	Lung adenocarcinoma	2	3	1	IV
GR012	Female	78	Lung adenocarcinoma	3	2	1	IV
GR013	Female	67	Lung adenocarcinoma	2	0	1	IV
GR014	Female	63	Lung adenocarcinoma	4	3	1	IV
GR015	Female	66	Lung adenocarcinoma	2	2	0	III
GR016	Female	65	Lung adenocarcinoma	1	0	0	I
GR017	Female	65	Lung adenocarcinoma	1	0	0	I
GR018	Male	73	Lung adenocarcinoma	2	2	0	III
GR019	Female	52	Lung adenocarcinoma	1	0	0	I
GR020	Female	45	Lung adenocarcinoma	2	1	0	III

GR021	Male	61	Lung adenocarcinoma	1	0	0	I
GR022	Male	32	Lung adenocarcinoma	3	2	1	I
GR023	Male	70	Lung adenocarcinoma	1	0	0	I
GR024	Female	73	Lung adenocarcinoma	1	0	0	I
GR025	Female	68	Lung adenocarcinoma	2	0	0	II
GR026	Male	58	Lung adenocarcinoma	1	0	0	I
GR027	Male	63	Lung adenocarcinoma	1	0	0	I
GR028	Female	60	Lung adenocarcinoma	1	0	0	I
GR029	Male	60	Lung squamous cell carcinomas	1	0	0	I
GR030	Male	57	Lung squamous cell carcinomas	3	2	1	IV
GR031	Male	70	Lung squamous cell carcinomas	4	3	1	IV
GR032	Male	62	Lung squamous cell carcinomas	2	1	0	II
GR033	Male	68	Lung squamous cell carcinomas	4	0	1	IV
GR034	Female	73	Lung adenocarcinoma	4	1	0	III
GR035	Male	53	Lung squamous cell carcinomas	2	2	0	III
GR036	Male	65	Lung squamous cell carcinomas	3	2	1	IV
GR037	Male	66	Lung squamous cell carcinomas	4	3	0	IV
GR038	Male	69	Lung squamous cell carcinomas	3	3	0	III
OR001	Female	76	Lung adenocarcinoma	3	2	0	III
OR002	Female	67	Lung adenocarcinoma	3	3	1	IV
OR003	Male	68	Lung squamous cell carcinomas	3	2	0	III
OR004	Female	80	Lung adenocarcinoma	4	0	1	IV
OR005	Female	73	Lung adenocarcinoma	2	2	0	III
OR006	Male	56	Lung adenocarcinoma	NA	NA	NA	NA
OR007	Female	60	Lung adenocarcinoma	1	1	0	IV
OR008	Male	48	Lung adenocarcinoma	4	1	0	III
OR009	Female	74	Lung adenocarcinoma	3	0	1	IV
OR010	Female	50	Lung adenocarcinoma	NA	NA	NA	NA
OR011	Female	60	Lung adenocarcinoma	1	1	0	II
OR012	Male	44	Lung adenocarcinoma	2	2	0	III
OR013	Female	69	Lung adenocarcinoma	4	3	1	IV
OR014	Male	66	Lung squamous cell carcinomas	1	3	1	IV
OR015	Male	66	Lung squamous cell carcinomas	4	0	1	IV
OR016	Male	57	Lung squamous cell carcinomas	3	1	0	III
OR017	Male	58	Lung squamous cell carcinomas	1	1	0	II
OR018	Female	71	Lung squamous cell carcinomas	2	3	1	IV
OR019	Male	67	Lung squamous cell carcinomas	4	0	1	IV
OR020	Male	67	Lung squamous cell carcinomas	4	2	1	IV
OR021	Male	68	Lung squamous cell carcinomas	2	1	0	II
OR022	Male	74	Lung squamous cell carcinomas	4	0	1	IV
OR023	Male	53	Lung squamous cell carcinomas	4	3	1	IV
OR024	Male	61	Lung squamous cell carcinomas	4	0	0	III
OR025	Male	53	Lung squamous cell carcinomas	3	1	0	III
OR026	Male	58	Lung squamous cell carcinomas	3	2	0	III

OR027	Male	60	Lung squamous cell carcinomas	2	2	0	III
OR028	Male	56	Lung squamous cell carcinomas	4	1	0	III
OR029	Male	67	Lung squamous cell carcinomas	3	2	0	III
OR030	Male	69	Lung squamous cell carcinomas	2	2	0	III
OR031	Male	55	Lung squamous cell carcinomas	4	0	0	III
OR032	Male	75	Lung squamous cell carcinomas	4	2	0	IV
OR033	Male	65	Lung squamous cell carcinomas	3	0	0	III

Supplementary Table 7. GO enrichment for the genes overlapping with DMRs from HCC827 vs HCC827/GR

Term	Database	ID	Input number	Background number	Rich factor	P Value	Corrected P Value
nucleoplasm	cellular_component	GO:0005654	260	3630	2.06	3.39E-10	9.88E-07
RNA binding	molecular_function	GO:0003723	121	1366	2.55	5.67E-10	9.88E-07
mRNA splicing, via spliceosome	biological_process	GO:0000398	33	246	3.86	5.22E-07	0.000606476
nuclear speck	cellular_component	GO:0016607	42	400	3.02	5.74E-06	0.004997155
cadherin binding	molecular_function	GO:0045296	32	305	3.02	6.98E-05	0.043166204
RNA splicing	biological_process	GO:0008380	21	160	3.77	7.43E-05	0.043166204
nucleolus	cellular_component	GO:0005730	66	839	2.26	0.000111253	0.055372224
negative regulation of transcription by RNA polymerase II	biological_process	GO:0000122	64	832	2.21	0.000244948	0.106674866
mRNA processing	biological_process	GO:0006397	19	158	3.46	0.000432632	0.167476596
cell cycle	biological_process	GO:0007049	25	245	2.93	0.000587843	0.204804667
RNA polymerase II CTD heptapeptide repeat kinase activity	molecular_function	GO:0008353	5	12	11.97	0.000723622	0.212589476
RNA metabolic process	biological_process	GO:0016070	9	46	5.62	0.000732226	0.212589476
negative regulation of transcription, DNA-templated	biological_process	GO:0045892	43	536	2.31	0.001094813	0.293128787
transcription by RNA polymerase II	biological_process	GO:0006366	20	187	3.07	0.0011779	0.293128787
preribosome, large subunit precursor	cellular_component	GO:0030687	6	23	7.5	0.001609571	0.360587641
response to endoplasmic reticulum stress	biological_process	GO:0034976	11	75	4.21	0.001655971	0.360587641
Cajal body	cellular_component	GO:0015030	9	53	4.88	0.00176201	0.3611085
protein domain specific binding	molecular_function	GO:0019904	23	239	2.77	0.001913545	0.370377333
regulation of phosphoprotein phosphatase activity	biological_process	GO:0043666	8	44	5.23	0.002145635	0.393441668

ribosomal large subunit assembly	biological_process	GO:0000027	6	26	6.63	0.002736077	0.456944662
focal adhesion	cellular_component	GO:0005925	34	418	2.34	0.002759696	0.456944662
protein phosphatase regulator activity	molecular_function	GO:0019888	7	36	5.59	0.002885414	0.456944662
costamere	cellular_component	GO:0043034	5	19	7.56	0.003822249	0.53849829
catalytic step 2 spliceosome	cellular_component	GO:0071013	11	86	3.68	0.004298505	0.53849829
protein localization to adherens junction	biological_process	GO:0071896	3	5	17.24	0.004301213	0.53849829
granular component	cellular_component	GO:0001652	3	5	17.24	0.004301213	0.53849829
protein phosphorylation	biological_process	GO:0006468	35	451	2.23	0.004797459	0.53849829
positive regulation of protein import into nucleus	biological_process	GO:0042307	7	40	5.03	0.00481767	0.53849829
activation of MAPKKK activity	biological_process	GO:0000185	4	12	9.58	0.004824677	0.53849829
regulation of alternative mRNA splicing, via spliceosome	biological_process	GO:0000381	10	75	3.83	0.004872594	0.53849829
mRNA binding	molecular_function	GO:0003729	19	200	2.73	0.005090544	0.53849829
negative regulation of protein serine/threonine kinase activity	biological_process	GO:0071901	5	21	6.84	0.005490124	0.53849829
viral process	biological_process	GO:0016032	35	456	2.21	0.005602738	0.53849829
negative regulation of type I interferon production	biological_process	GO:0032480	6	31	5.56	0.005796374	0.53849829
regulation of circadian rhythm	biological_process	GO:0042752	9	65	3.98	0.005936122	0.53849829
positive regulation of type I interferon-mediated signaling pathway	biological_process	GO:0060340	4	13	8.84	0.00608654	0.53849829
negative regulation of receptor signaling pathway via JAK-STAT	biological_process	GO:0046426	4	13	8.84	0.00608654	0.53849829
neural tube closure	biological_process	GO:0001843	10	78	3.68	0.006228169	0.53849829
RNA polymerase III type 3 promoter sequence-specific DNA binding	molecular_function	GO:0001006	3	6	14.37	0.006236681	0.53849829

response to alkaloid	biological_process	GO:0043279	3	6	14.37	0.006236681	0.53849829
p53 binding	molecular_function	GO:0002039	9	66	3.92	0.006481834	0.53849829
apical junction complex	cellular_component	GO:0043296	5	22	6.53	0.006491656	0.53849829
regulation of RNA splicing	biological_process	GO:0043484	7	43	4.68	0.006808703	0.551663308
cellular response to dopamine	biological_process	GO:1903351	4	14	8.21	0.007549866	0.578879936
viral genome replication	biological_process	GO:0019079	4	14	8.21	0.007549866	0.578879936
regulation of cell cycle	biological_process	GO:0051726	13	121	3.09	0.007643076	0.578879936
cellular protein modification process	biological_process	GO:0006464	10	81	3.55	0.007860036	0.582646107
intrinsic apoptotic signaling pathway in response to endoplasmic reticulum stress	biological_process	GO:0070059	6	34	5.07	0.00853604	0.603478178
N6-methyladenosine-containing RNA binding	molecular_function	GO:1990247	3	7	12.32	0.00861318	0.603478178
positive regulation of transcription, DNA-templated	biological_process	GO:0045893	40	556	2.07	0.00871105	0.603478178
regulation of autophagy	biological_process	GO:0010506	9	70	3.69	0.009057782	0.603478178
negative regulation of stem cell differentiation	biological_process	GO:2000737	4	15	7.66	0.009226854	0.603478178
positive regulation of transcription regulatory region DNA binding	biological_process	GO:2000679	4	15	7.66	0.009226854	0.603478178
regulation of cholesterol biosynthetic process	biological_process	GO:0045540	6	35	4.93	0.009626177	0.603478178
transcription factor binding	molecular_function	GO:0008134	26	325	2.3	0.009648652	0.603478178
ubiquitin protein ligase binding	molecular_function	GO:0031625	24	294	2.35	0.010108773	0.603478178
RNA splicing, via transesterification reactions	biological_process	GO:0000375	5	25	5.75	0.010251393	0.603478178
chromatin binding	molecular_function	GO:0003682	33	443	2.14	0.010281918	0.603478178
ribonucleoprotein complex	cellular_component	GO:1990904	15	156	2.76	0.010787173	0.603478178
negative regulation of epithelial cell migration	biological_process	GO:0010633	4	16	7.18	0.011128683	0.603478178
positive regulation of mammary gland epithelial cell proliferation	biological_process	GO:0033601	3	8	10.78	0.011450146	0.603478178

microtubule minus-end	cellular_component	GO:0036449	3	8	10.78	0.011450146	0.603478178
histone H2A-K119 monoubiquitination	biological_process	GO:0036353	3	8	10.78	0.011450146	0.603478178
mammary gland epithelial cell proliferation	biological_process	GO:0033598	3	8	10.78	0.011450146	0.603478178
minor groove of adenine-thymine-rich DNA binding	molecular_function	GO:0003680	3	8	10.78	0.011450146	0.603478178
mitotic spindle	cellular_component	GO:0072686	11	100	3.16	0.01159525	0.603478178
negative regulation of Ras protein signal transduction	biological_process	GO:0046580	5	26	5.53	0.011778564	0.603478178
mRNA 3'-UTR AU-rich region binding	molecular_function	GO:0035925	5	26	5.53	0.011778564	0.603478178
phagocytic vesicle membrane	cellular_component	GO:0030670	9	74	3.5	0.012341399	0.623151223
protein-containing complex	cellular_component	GO:0032991	43	623	1.98	0.012604807	0.627359267
positive regulation of brown fat cell differentiation	biological_process	GO:0090336	4	17	6.76	0.013265464	0.635876499
3'-UTR-mediated mRNA destabilization	biological_process	GO:0061158	4	17	6.76	0.013265464	0.635876499
mRNA stabilization	biological_process	GO:0048255	5	27	5.32	0.013453098	0.635876499
regulation of signal transduction by p53 class mediator	biological_process	GO:1901796	13	131	2.85	0.013505988	0.635876499
somatic stem cell population maintenance	biological_process	GO:0035019	8	63	3.65	0.014340445	0.656764151
bleb	cellular_component	GO:0032059	3	9	9.58	0.014761542	0.656764151
cAMP response element binding protein binding	molecular_function	GO:0008140	3	9	9.58	0.014761542	0.656764151
activation of GTPase activity	biological_process	GO:0090630	11	104	3.04	0.014836355	0.656764151
membrane organization	biological_process	GO:0061024	13	133	2.81	0.015009478	0.656764151
Cul4-RING E3 ubiquitin ligase complex	cellular_component	GO:0080008	5	28	5.13	0.015280755	0.656764151
transcription corepressor activity	molecular_function	GO:0003714	17	194	2.52	0.015351966	0.656764151
regulation of phosphatidylinositol 3-kinase activity	biological_process	GO:0043551	4	18	6.39	0.015646218	0.656764151

GABA receptor binding	molecular_function	GO:0050811	4	18	6.39	0.015646218	0.656764151
rRNA binding	molecular_function	GO:0019843	6	40	4.31	0.016590935	0.688128771
cyclin-dependent protein serine/threonine kinase activity	molecular_function	GO:0004693	5	29	4.95	0.017266935	0.695167783
regulation of protein localization to plasma membrane	biological_process	GO:1903076	4	19	6.05	0.018278856	0.695167783
protein phosphatase type 2A complex	cellular_component	GO:0000159	4	19	6.05	0.018278856	0.695167783
negative regulation of cellular senescence	biological_process	GO:2000773	4	19	6.05	0.018278856	0.695167783
negative regulation of G1/S transition of mitotic cell cycle	biological_process	GO:2000134	6	41	4.21	0.018313852	0.695167783
SH2 domain binding	molecular_function	GO:0042169	6	41	4.21	0.018313852	0.695167783
Tat protein binding	molecular_function	GO:0030957	3	10	8.62	0.01855643	0.695167783
primary miRNA processing	biological_process	GO:0031053	3	10	8.62	0.01855643	0.695167783
ER membrane protein complex	cellular_component	GO:0072546	3	10	8.62	0.01855643	0.695167783
intracellular anatomical structure	cellular_component	GO:0005622	12	123	2.8	0.01917565	0.697398707
cyclin binding	molecular_function	GO:0030332	5	30	4.79	0.019416669	0.697398707
MLL1 complex	cellular_component	GO:0071339	5	30	4.79	0.019416669	0.697398707
positive regulation of peptidyl-threonine phosphorylation	biological_process	GO:0010800	5	30	4.79	0.019416669	0.697398707
phosphotyrosine residue binding	molecular_function	GO:0001784	6	42	4.11	0.02015514	0.701559776
protein-containing complex binding	molecular_function	GO:0044877	26	349	2.14	0.02067882	0.701559776
transcription repressor complex	cellular_component	GO:0017053	7	55	3.66	0.021088973	0.701559776
cellular glucose homeostasis	biological_process	GO:0001678	4	20	5.75	0.021170177	0.701559776
microtubule plus-end binding	molecular_function	GO:0051010	4	20	5.75	0.021170177	0.701559776
neurotrophin TRK receptor signaling pathway	biological_process	GO:0048011	4	20	5.75	0.021170177	0.701559776
endomembrane system	cellular_component	GO:0012505	12	125	2.76	0.021259568	0.701559776

negative regulation of cell growth	biological_process	GO:0030308	12	125	2.76	0.021259568	0.701559776
cytosol	cellular_component	GO:0005829	273	5095	1.54	0.021344815	0.701559776
pre-mRNA intronic binding	molecular_function	GO:0097157	3	11	7.84	0.022839511	0.716169987
positive regulation of histone deacetylation	biological_process	GO:0031065	3	11	7.84	0.022839511	0.716169987
regulation of microtubule polymerization	biological_process	GO:0031113	3	11	7.84	0.022839511	0.716169987
leucine zipper domain binding	molecular_function	GO:0043522	3	11	7.84	0.022839511	0.716169987
chromosome	cellular_component	GO:0005694	12	127	2.72	0.02350656	0.716169987
negative regulation of ERK1 and ERK2 cascade	biological_process	GO:0070373	8	70	3.28	0.02405032	0.716169987
positive regulation of gene expression, epigenetic	biological_process	GO:0045815	5	32	4.49	0.02422494	0.716169987
intermediate filament cytoskeleton organization	biological_process	GO:0045104	4	21	5.47	0.024325877	0.716169987
mRNA splice site selection	biological_process	GO:0006376	4	21	5.47	0.024325877	0.716169987
histone deacetylase binding	molecular_function	GO:0042826	11	114	2.77	0.025875321	0.716169987
fibrillar center	cellular_component	GO:0001650	12	129	2.67	0.025923312	0.716169987
U4/U6 x U5 tri-snRNP complex	cellular_component	GO:0046540	5	33	4.35	0.026891526	0.716169987
dendrite cytoplasm	cellular_component	GO:0032839	5	33	4.35	0.026891526	0.716169987
helicase activity	molecular_function	GO:0004386	5	33	4.35	0.026891526	0.716169987
extrinsic apoptotic signaling pathway via death domain receptors	biological_process	GO:0008625	5	33	4.35	0.026891526	0.716169987
early endosome membrane	cellular_component	GO:0031901	14	161	2.5	0.0274333	0.716169987
protein phosphatase binding	molecular_function	GO:0019903	9	86	3.01	0.027471012	0.716169987
cellular response to starvation	biological_process	GO:0009267	8	72	3.19	0.027509774	0.716169987
early phagosome	cellular_component	GO:0032009	3	12	7.18	0.02761161	0.716169987
positive regulation of keratinocyte migration	biological_process	GO:0051549	3	12	7.18	0.02761161	0.716169987
fatty-acyl-CoA biosynthetic process	biological_process	GO:0046949	3	12	7.18	0.02761161	0.716169987
5'-3' exonuclease activity	molecular_function	GO:0008409	3	12	7.18	0.02761161	0.716169987

negative regulation of tyrosine phosphorylation of STAT protein	biological_process	GO:0042532	3	12	7.18	0.02761161	0.716169987
signal transduction in response to DNA damage	biological_process	GO:0042770	3	12	7.18	0.02761161	0.716169987
actin filament network formation	biological_process	GO:0051639	3	12	7.18	0.02761161	0.716169987
histone H2A monoubiquitination	biological_process	GO:0035518	3	12	7.18	0.02761161	0.716169987
regulation of MAP kinase activity	biological_process	GO:0043405	3	12	7.18	0.02761161	0.716169987
lysine-acetylated histone binding	molecular_function	GO:0070577	4	22	5.23	0.027750559	0.716169987
RNA catabolic process	biological_process	GO:0006401	4	22	5.23	0.027750559	0.716169987
perinuclear region of cytoplasm	cellular_component	GO:0048471	45	697	1.86	0.02847245	0.723908235
azurophil granule membrane	cellular_component	GO:0035577	7	59	3.41	0.028649533	0.723908235
Ras protein signal transduction	biological_process	GO:0007265	8	73	3.15	0.029362402	0.723908235
cytoplasmic stress granule	cellular_component	GO:0010494	8	73	3.15	0.029362402	0.723908235
ATP-dependent chromatin remodeling	biological_process	GO:0043044	5	34	4.23	0.029737745	0.723908235
protein localization to nucleus	biological_process	GO:0034504	5	34	4.23	0.029737745	0.723908235
protein K48-linked deubiquitination	biological_process	GO:0071108	5	34	4.23	0.029737745	0.723908235
RNA helicase activity	molecular_function	GO:0003724	8	74	3.11	0.031299051	0.723908235
protein deubiquitination	biological_process	GO:0016579	20	262	2.19	0.031447193	0.723908235
positive regulation of dendritic spine development	biological_process	GO:0060999	4	23	5	0.03144776	0.723908235
signaling adaptor activity	molecular_function	GO:0035591	4	23	5	0.03144776	0.723908235
microtubule plus-end	cellular_component	GO:0035371	4	23	5	0.03144776	0.723908235
regulation of apoptotic process	biological_process	GO:0042981	16	197	2.33	0.032335938	0.723908235
DNA-dependent ATPase activity	molecular_function	GO:0008094	5	35	4.11	0.03276656	0.723908235
cellular response to nitrogen starvation	biological_process	GO:0006995	3	13	6.63	0.032870133	0.723908235
microtubule minus-end binding	molecular_function	GO:0051011	3	13	6.63	0.032870133	0.723908235
postsynaptic actin cytoskeleton organization	biological_process	GO:0098974	3	13	6.63	0.032870133	0.723908235

negative regulation of transcription elongation from RNA polymerase II promoter	biological_process	GO:0034244	3	13	6.63	0.032870133	0.723908235
establishment of epithelial cell polarity	biological_process	GO:0090162	3	13	6.63	0.032870133	0.723908235
transcription coactivator activity	molecular_function	GO:0003713	19	248	2.2	0.034264122	0.723908235
phosphatidylinositol biosynthetic process	biological_process	GO:0006661	8	76	3.03	0.035430253	0.723908235
protein kinase activity	molecular_function	GO:0004672	17	216	2.26	0.035571919	0.723908235
negative regulation of epidermal growth factor receptor signaling pathway	biological_process	GO:0042059	5	36	3.99	0.035980502	0.723908235
mitochondrial DNA metabolic process	biological_process	GO:0032042	2	5	11.5	0.036562057	0.723908235
positive regulation of chromosome segregation	biological_process	GO:0051984	2	5	11.5	0.036562057	0.723908235
histone demethylase activity (H3-K27 specific)	molecular_function	GO:0071558	2	5	11.5	0.036562057	0.723908235
CRD-mediated mRNA stabilization	biological_process	GO:0070934	2	5	11.5	0.036562057	0.723908235
positive regulation of endoplasmic reticulum unfolded protein response	biological_process	GO:1900103	2	5	11.5	0.036562057	0.723908235
apoptotic process involved in morphogenesis	biological_process	GO:0060561	2	5	11.5	0.036562057	0.723908235
endoplasmic reticulum tubular network membrane	cellular_component	GO:0098826	2	5	11.5	0.036562057	0.723908235
establishment of mitotic spindle localization	biological_process	GO:0040001	2	5	11.5	0.036562057	0.723908235
positive regulation of long-term neuronal synaptic plasticity	biological_process	GO:0048170	2	5	11.5	0.036562057	0.723908235
positive regulation of focal adhesion disassembly	Undef	GO:0120183	2	5	11.5	0.036562057	0.723908235
histone methyltransferase activity (H4-K20 specific)	molecular_function	GO:0042799	2	5	11.5	0.036562057	0.723908235
histone H3-K27 demethylation	biological_process	GO:0071557	2	5	11.5	0.036562057	0.723908235
interchromatin granule	cellular_component	GO:0035061	2	5	11.5	0.036562057	0.723908235
polar microtubule	cellular_component	GO:0005827	2	5	11.5	0.036562057	0.723908235

establishment or maintenance of cell polarity	biological_process	GO:0007163	6	49	3.52	0.036608635	0.723908235
regulation of translation	biological_process	GO:0006417	7	63	3.19	0.037856225	0.723908235
positive regulation of receptor recycling	biological_process	GO:0001921	3	14	6.16	0.038609474	0.723908235
apical protein localization	biological_process	GO:0045176	3	14	6.16	0.038609474	0.723908235
ruffle assembly	biological_process	GO:0097178	3	14	6.16	0.038609474	0.723908235
membrane fusion	biological_process	GO:0061025	5	37	3.88	0.039381669	0.723908235
protein autophosphorylation	biological_process	GO:0046777	14	170	2.37	0.039430512	0.723908235
U2-type precatalytic spliceosome	cellular_component	GO:0071005	6	50	3.45	0.039498031	0.723908235
spindle midzone	cellular_component	GO:0051233	4	25	4.6	0.039668691	0.723908235
nucleus	cellular_component	GO:0005634	274	5208	1.51	0.040558201	0.723908235
cellular response to amino acid stimulus	biological_process	GO:0071230	6	51	3.38	0.042527965	0.723908235
nuclear matrix	cellular_component	GO:0016363	10	109	2.64	0.042663604	0.723908235
protein C-terminus binding	molecular_function	GO:0008022	15	189	2.28	0.043635823	0.723908235
positive regulation of muscle cell differentiation	biological_process	GO:0051149	4	26	4.42	0.044194429	0.723908235
TBP-class protein binding	molecular_function	GO:0017025	4	26	4.42	0.044194429	0.723908235
fibroblast growth factor receptor signaling pathway	biological_process	GO:0008543	8	80	2.87	0.044760769	0.723908235
positive regulation of JNK cascade	biological_process	GO:0046330	8	80	2.87	0.044760769	0.723908235
hematopoietic stem cell proliferation	biological_process	GO:0071425	3	15	5.75	0.044821396	0.723908235
Ino80 complex	cellular_component	GO:0031011	3	15	5.75	0.044821396	0.723908235
G protein-coupled acetylcholine receptor signaling pathway	biological_process	GO:0007213	3	15	5.75	0.044821396	0.723908235
PRC1 complex	cellular_component	GO:0035102	3	15	5.75	0.044821396	0.723908235
1-phosphatidylinositol-3-kinase regulator activity	molecular_function	GO:0046935	3	15	5.75	0.044821396	0.723908235
negative regulation of cell-cell adhesion	biological_process	GO:0022408	3	15	5.75	0.044821396	0.723908235
positive regulation of dendrite development	biological_process	GO:1900006	3	15	5.75	0.044821396	0.723908235

NuRD complex	cellular_component	GO:0016581	3	15	5.75	0.044821396	0.723908235
establishment of endothelial intestinal barrier	biological_process	GO:0090557	3	15	5.75	0.044821396	0.723908235
cellular response to oxidative stress	biological_process	GO:0034599	9	95	2.72	0.04515664	0.723908235
ribosome binding	molecular_function	GO:0043022	6	52	3.32	0.045699817	0.723908235
positive regulation of type I interferon production	biological_process	GO:0032481	6	52	3.32	0.045699817	0.723908235
nuclear receptor transcription coactivator activity	molecular_function	GO:0030374	6	52	3.32	0.045699817	0.723908235
rhythmic process	biological_process	GO:0048511	7	66	3.05	0.04590663	0.723908235
hematopoietic progenitor cell differentiation	biological_process	GO:0002244	7	66	3.05	0.04590663	0.723908235
mitotic metaphase plate congression	biological_process	GO:0007080	5	39	3.68	0.046751907	0.723908235
internal peptidyl-lysine acetylation	biological_process	GO:0018393	2	6	9.58	0.047315672	0.723908235
SAGA-type complex	cellular_component	GO:0070461	2	6	9.58	0.047315672	0.723908235
sex chromatin	cellular_component	GO:0001739	2	6	9.58	0.047315672	0.723908235
CRD-mediated mRNA stability complex	cellular_component	GO:0070937	2	6	9.58	0.047315672	0.723908235
membrane invagination	biological_process	GO:0010324	2	6	9.58	0.047315672	0.723908235
morphogenesis of a polarized epithelium	biological_process	GO:0001738	2	6	9.58	0.047315672	0.723908235
negative regulation of ruffle assembly	biological_process	GO:1900028	2	6	9.58	0.047315672	0.723908235
chromo shadow domain binding	molecular_function	GO:0070087	2	6	9.58	0.047315672	0.723908235
U4 snRNA binding	molecular_function	GO:0030621	2	6	9.58	0.047315672	0.723908235
monoubiquitinated protein deubiquitination	biological_process	GO:0035520	2	6	9.58	0.047315672	0.723908235
regulation of SMAD protein signal transduction	biological_process	GO:0060390	2	6	9.58	0.047315672	0.723908235
chromosome separation	biological_process	GO:0051304	2	6	9.58	0.047315672	0.723908235
dense body	cellular_component	GO:0097433	2	6	9.58	0.047315672	0.723908235
lens fiber cell morphogenesis	biological_process	GO:0070309	2	6	9.58	0.047315672	0.723908235
late endosome to Golgi transport	biological_process	GO:0034499	2	6	9.58	0.047315672	0.723908235
common-partner SMAD protein phosphorylation	biological_process	GO:0007182	2	6	9.58	0.047315672	0.723908235

paraspeckles	cellular_component	GO:0042382	2	6	9.58	0.047315672	0.723908235
PcG protein complex	cellular_component	GO:0031519	4	27	4.26	0.04899678	0.723908235
cellular response to fibroblast growth factor stimulus	biological_process	GO:0044344	4	27	4.26	0.04899678	0.723908235
positive regulation of viral transcription	biological_process	GO:0050434	4	27	4.26	0.04899678	0.723908235
negative regulation of microtubule depolymerization	biological_process	GO:0007026	4	27	4.26	0.04899678	0.723908235
ribosomal large subunit biogenesis	biological_process	GO:0042273	4	27	4.26	0.04899678	0.723908235
bone morphogenesis	biological_process	GO:0060349	4	27	4.26	0.04899678	0.723908235
transcription regulator complex	cellular_component	GO:0005667	17	226	2.16	0.049411501	0.723908235
midbody	cellular_component	GO:0030496	13	160	2.34	0.04982566	0.723908235

Supplementary Table 8. GO enrichment for the genes overlapping with DMRs from HCC827 vs HCC827/OR

Term	Database	ID	Input number	Background number	Rich factor	P Value	Corrected P Value
nucleoplasm	cellular_component	GO:0005654	204	3630	2.03	7.14E-08	0.00022603
RNA binding	molecular_function	GO:0003723	91	1366	2.4	6.71E-07	0.001062704
mRNA splicing, via spliceosome	biological_process	GO:0000398	28	246	4.1	1.09E-06	0.00114539
ribonucleoprotein complex	cellular_component	GO:1990904	18	156	4.16	7.15E-05	0.056576946
negative regulation of transcription by RNA polymerase II	biological_process	GO:0000122	52	832	2.25	0.000578855	0.315851894
nuclear speck	cellular_component	GO:0016607	30	400	2.7	0.000598582	0.315851894
mRNA binding	molecular_function	GO:0003729	18	200	3.24	0.001114486	0.504066091
mRNA processing	biological_process	GO:0006397	15	158	3.42	0.001721261	0.56821575
cell cycle	biological_process	GO:0007049	20	245	2.94	0.00182302	0.56821575
regulation of RNA splicing	biological_process	GO:0043484	7	43	5.87	0.002101406	0.56821575
mRNA splice site selection	biological_process	GO:0006376	5	21	8.58	0.002185792	0.56821575
RNA polymerase II CTD heptapeptide repeat kinase activity	molecular_function	GO:0008353	4	12	12.01	0.002222428	0.56821575
positive regulation of long-term neuronal synaptic plasticity	biological_process	GO:0048170	3	5	21.62	0.002333166	0.56821575
RNA processing	biological_process	GO:0006396	8	58	4.97	0.002644746	0.598090412
perinuclear region of cytoplasm	cellular_component	GO:0048471	42	697	2.17	0.003357391	0.634206585
response to alkaloid	biological_process	GO:0043279	3	6	18.02	0.003405405	0.634206585
paraspeckles	cellular_component	GO:0042382	3	6	18.02	0.003405405	0.634206585
cytosol	cellular_component	GO:0005829	230	5095	1.63	0.004430979	0.697959767
pronucleus	cellular_component	GO:0045120	3	7	15.45	0.004733973	0.697959767
apoptotic process	biological_process	GO:0006915	34	546	2.24	0.004859045	0.697959767

cellular response to unfolded protein	biological_process	GO:0034620	5	26	6.93	0.004859094	0.697959767
regulation of JNK cascade	biological_process	GO:0046328	4	16	9.01	0.005269721	0.697959767
heterochromatin	cellular_component	GO:0000792	5	27	6.67	0.005589165	0.697959767
primary miRNA binding	molecular_function	GO:0070878	3	8	13.51	0.006334423	0.697959767
transcription regulator complex	cellular_component	GO:0005667	17	226	2.71	0.008038098	0.697959767
costamere	cellular_component	GO:0043034	4	19	7.59	0.008834276	0.697959767
circadian rhythm	biological_process	GO:0007623	8	74	3.9	0.009884175	0.697959767
negative regulation of inclusion body assembly	biological_process	GO:0090084	3	10	10.81	0.010399613	0.697959767
ER membrane protein complex	cellular_component	GO:0072546	3	10	10.81	0.010399613	0.697959767
response to endoplasmic reticulum stress	biological_process	GO:0034976	8	75	3.84	0.010599046	0.697959767
regulation of alternative mRNA splicing, via spliceosome	biological_process	GO:0000381	8	75	3.84	0.010599046	0.697959767
intracellular protein transport	biological_process	GO:0006886	19	273	2.51	0.011061205	0.697959767
RNA metabolic process	biological_process	GO:0016070	6	46	4.7	0.011135846	0.697959767
ubiquitin protein ligase binding	molecular_function	GO:0031625	20	294	2.45	0.011589672	0.697959767
dendrite cytoplasm	cellular_component	GO:0032839	5	33	5.46	0.011649938	0.697959767
protein localization to Golgi apparatus	biological_process	GO:0034067	4	21	6.86	0.011916678	0.697959767
regulation of cellular response to heat	biological_process	GO:1900034	8	77	3.74	0.012142452	0.697959767
response to activity	biological_process	GO:0014823	6	47	4.6	0.012180681	0.697959767
focal adhesion	cellular_component	GO:0005925	26	418	2.24	0.012592999	0.697959767
MAP-kinase scaffold activity	molecular_function	GO:0005078	3	11	9.83	0.012882562	0.697959767
positive regulation of production of miRNAs involved in gene silencing by miRNA	biological_process	GO:1903800	3	11	9.83	0.012882562	0.697959767
cellular response to potassium ion	biological_process	GO:0035865	3	11	9.83	0.012882562	0.697959767
regulation of translation	biological_process	GO:0006417	7	63	4	0.013507994	0.697959767
RNA catabolic process	biological_process	GO:0006401	4	22	6.55	0.01368593	0.697959767

chromatin binding	molecular_function	GO:0003682	27	443	2.2	0.014042245	0.697959767
RNA polymerase II cis-regulatory region sequence-specific DNA binding	molecular_function	GO:0000978	37	656	2.03	0.014056901	0.697959767
cellular response to reactive oxygen species	biological_process	GO:0034614	5	35	5.15	0.014392202	0.697959767
cellular response to leukemia inhibitory factor	biological_process	GO:1990830	9	97	3.34	0.015239308	0.697959767
preribosome, large subunit precursor	cellular_component	GO:0030687	4	23	6.27	0.015613384	0.697959767
poly(A) binding	molecular_function	GO:0008143	4	23	6.27	0.015613384	0.697959767
gene silencing by miRNA	biological_process	GO:0035195	3	12	9.01	0.015674221	0.697959767
protein phosphatase regulator activity	molecular_function	GO:0019888	5	36	5.01	0.015912692	0.697959767
chromatin DNA binding	molecular_function	GO:0031490	7	66	3.82	0.016731754	0.697959767
cellular response to amino acid stimulus	biological_process	GO:0071230	6	51	4.24	0.017049695	0.697959767
negative regulation of transcription, DNA-templated	biological_process	GO:0045892	31	536	2.08	0.017098735	0.697959767
positive regulation of transcription by RNA polymerase II	biological_process	GO:0045944	59	1159	1.83	0.017147892	0.697959767
cellular response to fatty acid	biological_process	GO:0071398	4	24	6.01	0.017703089	0.697959767
positive regulation of type I interferon production	biological_process	GO:0032481	6	52	4.16	0.018449002	0.697959767
negative regulation of kinase activity	biological_process	GO:0033673	3	13	8.32	0.018778481	0.697959767
chaperone complex	cellular_component	GO:0101031	3	13	8.32	0.018778481	0.697959767
positive regulation of transcription elongation from RNA polymerase II promoter	biological_process	GO:0032968	3	13	8.32	0.018778481	0.697959767
response to unfolded protein	biological_process	GO:0006986	6	53	4.08	0.019924761	0.697959767
DNA-binding transcription repressor activity, RNA polymerase II-specific	molecular_function	GO:0001227	17	253	2.42	0.020753204	0.697959767
phosphoprotein phosphatase activity	molecular_function	GO:0004721	5	39	4.62	0.021103704	0.697959767
histone deacetylation	biological_process	GO:0016575	5	39	4.62	0.021103704	0.697959767
protein phosphatase binding	molecular_function	GO:0019903	8	86	3.35	0.021176273	0.697959767

insulin receptor signaling pathway	biological_process	GO:0008286	8	86	3.35	0.021176273	0.697959767
regulation of cholesterol metabolic process	biological_process	GO:0090181	3	14	7.72	0.02219747	0.697959767
ruffle assembly	biological_process	GO:0097178	3	14	7.72	0.02219747	0.697959767
Golgi ribbon formation	biological_process	GO:0090161	3	14	7.72	0.02219747	0.697959767
S100 protein binding	molecular_function	GO:0044548	3	14	7.72	0.02219747	0.697959767
positive regulation of RNA splicing	biological_process	GO:0033120	3	14	7.72	0.02219747	0.697959767
mRNA 3'-UTR AU-rich region binding	molecular_function	GO:0035925	4	26	5.54	0.02238315	0.697959767
kinetochore	cellular_component	GO:0000776	8	87	3.31	0.022411612	0.697959767
positive regulation of pri-miRNA transcription by RNA polymerase II	biological_process	GO:1902895	5	40	4.5	0.023051867	0.697959767
positive regulation of ATPase activity	biological_process	GO:0032781	5	40	4.5	0.023051867	0.697959767
cellular response to heat	biological_process	GO:0034605	6	55	3.93	0.023112324	0.697959767
transcription repressor complex	cellular_component	GO:0017053	6	55	3.93	0.023112324	0.697959767
nucleolus	cellular_component	GO:0005730	44	839	1.89	0.023468924	0.697959767
RNA splicing	biological_process	GO:0008380	12	160	2.7	0.023743658	0.697959767
N-acylethanolamine metabolic process	biological_process	GO:0070291	2	5	14.42	0.024493187	0.697959767
positive regulation of chromosome segregation	biological_process	GO:0051984	2	5	14.42	0.024493187	0.697959767
histone demethylase activity (H3-K27 specific)	molecular_function	GO:0071558	2	5	14.42	0.024493187	0.697959767
positive regulation of isotype switching to IgA isotypes	biological_process	GO:0048298	2	5	14.42	0.024493187	0.697959767
carbon-sulfur lyase activity	molecular_function	GO:0016846	2	5	14.42	0.024493187	0.697959767
establishment of protein localization to mitochondrion	biological_process	GO:0072655	2	5	14.42	0.024493187	0.697959767
UMP salvage	biological_process	GO:0044206	2	5	14.42	0.024493187	0.697959767
external side of apical plasma membrane	cellular_component	GO:0098591	2	5	14.42	0.024493187	0.697959767
CRD-mediated mRNA stabilization	biological_process	GO:0070934	2	5	14.42	0.024493187	0.697959767

histone H3-K27 demethylation	biological_process	GO:0071557	2	5	14.42	0.024493187	0.697959767
negative regulation of gene silencing by miRNA	biological_process	GO:0060965	2	5	14.42	0.024493187	0.697959767
3M complex	cellular_component	GO:1990393	2	5	14.42	0.024493187	0.697959767
COPI-coated vesicle membrane	cellular_component	GO:0030663	2	5	14.42	0.024493187	0.697959767
interchromatin granule	cellular_component	GO:0035061	2	5	14.42	0.024493187	0.697959767
regulation of microvillus assembly	biological_process	GO:0032534	2	5	14.42	0.024493187	0.697959767
negative regulation of amyloid fibril formation	biological_process	GO:1905907	2	5	14.42	0.024493187	0.697959767
early endosome membrane	cellular_component	GO:0031901	12	161	2.69	0.024699994	0.697959767
cellular response to starvation	biological_process	GO:0009267	7	72	3.5	0.02473855	0.697959767
protein domain specific binding	molecular_function	GO:0019904	16	239	2.41	0.02499561	0.697959767
endomembrane system	cellular_component	GO:0012505	10	125	2.88	0.025822809	0.697959767
Ino80 complex	cellular_component	GO:0031011	3	15	7.21	0.025931717	0.697959767
positive regulation of translation	biological_process	GO:0045727	7	73	3.46	0.026289673	0.697959767
cytoplasmic stress granule	cellular_component	GO:0010494	7	73	3.46	0.026289673	0.697959767
Hsp90 protein binding	molecular_function	GO:0051879	5	42	4.29	0.027287522	0.697959767
mRNA cis splicing, via spliceosome	biological_process	GO:0045292	4	28	5.15	0.027749327	0.697959767
condensed nuclear chromosome	cellular_component	GO:0000794	4	28	5.15	0.027749327	0.697959767
cadherin binding	molecular_function	GO:0045296	19	305	2.25	0.029007017	0.697959767
positive regulation of I-kappaB kinase/NF-kappaB signaling	biological_process	GO:0043123	13	185	2.53	0.029578678	0.697959767
insulin-like growth factor receptor binding	molecular_function	GO:0005159	3	16	6.76	0.0299803	0.697959767
anterograde synaptic vesicle transport	biological_process	GO:0048490	3	16	6.76	0.0299803	0.697959767
positive regulation of calcineurin-NFAT signaling cascade	biological_process	GO:0070886	3	16	6.76	0.0299803	0.697959767
negative regulation of epithelial cell migration	biological_process	GO:0010633	3	16	6.76	0.0299803	0.697959767
protein targeting to vacuole	biological_process	GO:0006623	3	16	6.76	0.0299803	0.697959767

autophagosome assembly	biological_process	GO:0000045	6	59	3.67	0.03047409	0.697959767
mRNA 3'-end processing	biological_process	GO:0031124	6	59	3.67	0.03047409	0.697959767
neural tube development	biological_process	GO:0021915	4	29	4.97	0.030694992	0.697959767
cyclin-dependent protein serine/threonine kinase activity	molecular_function	GO:0004693	4	29	4.97	0.030694992	0.697959767
negative regulation of mitotic cell cycle	biological_process	GO:0045930	4	29	4.97	0.030694992	0.697959767
liver development	biological_process	GO:0001889	7	76	3.32	0.031336692	0.697959767
nuclear stress granule	cellular_component	GO:0097165	2	6	12.01	0.03187986	0.697959767
COPI coating of Golgi vesicle	biological_process	GO:0048205	2	6	12.01	0.03187986	0.697959767
microtubule bundle	cellular_component	GO:0097427	2	6	12.01	0.03187986	0.697959767
thyroid hormone transmembrane transporter activity	molecular_function	GO:0015349	2	6	12.01	0.03187986	0.697959767
lymphocyte proliferation	biological_process	GO:0046651	2	6	12.01	0.03187986	0.697959767
mRNA transcription	biological_process	GO:0009299	2	6	12.01	0.03187986	0.697959767
SAGA-type complex	cellular_component	GO:0070461	2	6	12.01	0.03187986	0.697959767
sulfur amino acid catabolic process	biological_process	GO:0000098	2	6	12.01	0.03187986	0.697959767
CRD-mediated mRNA stability complex	cellular_component	GO:0070937	2	6	12.01	0.03187986	0.697959767
negative regulation of ruffle assembly	biological_process	GO:1900028	2	6	12.01	0.03187986	0.697959767
complement binding	molecular_function	GO:0001848	2	6	12.01	0.03187986	0.697959767
tooth eruption	biological_process	GO:0044691	2	6	12.01	0.03187986	0.697959767
regulation of skeletal muscle cell differentiation	biological_process	GO:2001014	2	6	12.01	0.03187986	0.697959767
aggresome assembly	biological_process	GO:0070842	2	6	12.01	0.03187986	0.697959767
C3HC4-type RING finger domain binding	molecular_function	GO:0055131	2	6	12.01	0.03187986	0.697959767
late endosome to Golgi transport	biological_process	GO:0034499	2	6	12.01	0.03187986	0.697959767
regulation of phosphoprotein phosphatase activity	biological_process	GO:0043666	5	44	4.1	0.031987267	0.697959767
negative regulation of cell population proliferation	biological_process	GO:0008285	23	394	2.1	0.032778568	0.697959767
chaperone-mediated protein folding	biological_process	GO:0061077	4	30	4.81	0.033817648	0.697959767

cyclin binding	molecular_function	GO:0030332	4	30	4.81	0.033817648	0.697959767
trans-Golgi network transport vesicle	cellular_component	GO:0030140	3	17	6.36	0.03434099	0.697959767
positive regulation of brown fat cell differentiation	biological_process	GO:0090336	3	17	6.36	0.03434099	0.697959767
cellular response to low-density lipoprotein particle stimulus	biological_process	GO:0071404	3	17	6.36	0.03434099	0.697959767
U2-type prespliceosome	cellular_component	GO:0071004	3	17	6.36	0.03434099	0.697959767
cellular response to organic cyclic compound	biological_process	GO:0071407	5	45	4	0.034514875	0.697959767
positive regulation of MAP kinase activity	biological_process	GO:0043406	6	61	3.54	0.034669545	0.697959767
membrane organization	biological_process	GO:0061024	10	133	2.71	0.036306361	0.697959767
anterograde axonal transport	biological_process	GO:0008089	4	31	4.65	0.037118224	0.697959767
positive regulation of phosphatidylinositol 3-kinase activity	biological_process	GO:0043552	4	31	4.65	0.037118224	0.697959767
Golgi organization	biological_process	GO:0007030	9	115	2.82	0.037287623	0.697959767
cellular response to insulin stimulus	biological_process	GO:0032869	8	97	2.97	0.037649365	0.697959767
positive regulation of stress-activated MAPK cascade	biological_process	GO:0032874	3	18	6.01	0.039010381	0.697959767
RNA polymerase III complex	cellular_component	GO:0005666	3	18	6.01	0.039010381	0.697959767
camera-type eye morphogenesis	biological_process	GO:0048593	3	18	6.01	0.039010381	0.697959767
RNA polymerase binding	molecular_function	GO:0070063	3	18	6.01	0.039010381	0.697959767
alternative mRNA splicing, via spliceosome	biological_process	GO:0000380	3	18	6.01	0.039010381	0.697959767
fibroblast growth factor receptor signaling pathway	biological_process	GO:0008543	7	80	3.15	0.039019948	0.697959767
positive regulation of transcription, DNA-templated	biological_process	GO:0045893	30	556	1.94	0.03967574	0.697959767
positive regulation by host of viral genome replication	biological_process	GO:0044829	2	7	10.3	0.040015418	0.697959767
ubiquitinyl hydrolase activity	molecular_function	GO:0101005	2	7	10.3	0.040015418	0.697959767
N6-methyladenosine-containing RNA binding	molecular_function	GO:1990247	2	7	10.3	0.040015418	0.697959767

left/right pattern formation	biological_process	GO:0060972	2	7	10.3	0.040015418	0.697959767
chaperone-mediated autophagy	biological_process	GO:0061684	2	7	10.3	0.040015418	0.697959767
Smc5-Smc6 complex	cellular_component	GO:0030915	2	7	10.3	0.040015418	0.697959767
negative regulation of fibroblast apoptotic process	biological_process	GO:2000270	2	7	10.3	0.040015418	0.697959767
mannosyl-oligosaccharide 1,2-alpha-mannosidase activity	molecular_function	GO:0004571	2	7	10.3	0.040015418	0.697959767
spinal cord motor neuron cell fate specification	biological_process	GO:0021520	2	7	10.3	0.040015418	0.697959767
positive regulation of ER-associated ubiquitin-dependent protein catabolic process	biological_process	GO:1903071	2	7	10.3	0.040015418	0.697959767
modulation by virus of host cellular process	biological_process	GO:0019054	2	7	10.3	0.040015418	0.697959767
positive regulation of hair follicle development	biological_process	GO:0051798	2	7	10.3	0.040015418	0.697959767
salivary gland morphogenesis	biological_process	GO:0007435	2	7	10.3	0.040015418	0.697959767
negative regulation of interleukin-4 production	biological_process	GO:0032713	2	7	10.3	0.040015418	0.697959767
SMAD protein complex assembly	biological_process	GO:0007183	2	7	10.3	0.040015418	0.697959767
regulation of cellular pH	biological_process	GO:0030641	2	7	10.3	0.040015418	0.697959767
transcription corepressor activity	molecular_function	GO:0003714	13	194	2.42	0.040070595	0.697959767
manganese ion binding	molecular_function	GO:0030145	6	64	3.38	0.041630351	0.697959767
platelet formation	biological_process	GO:0030220	3	19	5.69	0.043984009	0.697959767
protein-membrane adaptor activity	molecular_function	GO:0043495	3	19	5.69	0.043984009	0.697959767
regulation of apoptotic process	biological_process	GO:0042981	13	197	2.38	0.044087429	0.697959767
regulation of circadian rhythm	biological_process	GO:0042752	6	65	3.33	0.044132073	0.697959767
U4/U6 x U5 tri-snRNP complex	cellular_component	GO:0046540	4	33	4.37	0.044254807	0.697959767
spleen development	biological_process	GO:0048536	4	33	4.37	0.044254807	0.697959767
extrinsic apoptotic signaling pathway via death domain receptors	biological_process	GO:0008625	4	33	4.37	0.044254807	0.697959767
mRNA export from nucleus	biological_process	GO:0006406	8	101	2.85	0.045318897	0.697959767

chromatin remodeling	biological_process	GO:0006338	8	102	2.83	0.047384713	0.697959767
ribonucleoprotein complex binding	molecular_function	GO:0043021	4	34	4.24	0.048090674	0.697959767
cerebral cortex cell migration	biological_process	GO:0021795	2	8	9.01	0.04883578	0.697959767
GDP metabolic process	biological_process	GO:0046710	2	8	9.01	0.04883578	0.697959767
maintenance of cell polarity	biological_process	GO:0030011	2	8	9.01	0.04883578	0.697959767
diacylglycerol biosynthetic process	biological_process	GO:0006651	2	8	9.01	0.04883578	0.697959767
response to type I interferon	biological_process	GO:0034340	2	8	9.01	0.04883578	0.697959767
clathrin heavy chain binding	molecular_function	GO:0032050	2	8	9.01	0.04883578	0.697959767
euchromatin	cellular_component	GO:0000791	2	8	9.01	0.04883578	0.697959767
mannose metabolic process	biological_process	GO:0006013	2	8	9.01	0.04883578	0.697959767
germinal center formation	biological_process	GO:0002467	2	8	9.01	0.04883578	0.697959767
exocrine pancreas development	biological_process	GO:0031017	2	8	9.01	0.04883578	0.697959767
regulation of early endosome to late endosome transport	biological_process	GO:2000641	2	8	9.01	0.04883578	0.697959767
death-inducing signaling complex assembly	biological_process	GO:0071550	2	8	9.01	0.04883578	0.697959767
minor groove of adenine-thymine-rich DNA binding	molecular_function	GO:0003680	2	8	9.01	0.04883578	0.697959767
hemidesmosome	cellular_component	GO:0030056	2	8	9.01	0.04883578	0.697959767
ubiquinol-cytochrome-c reductase activity	molecular_function	GO:0008121	2	8	9.01	0.04883578	0.697959767
establishment of Golgi localization	biological_process	GO:0051683	2	8	9.01	0.04883578	0.697959767
positive regulation of protein export from nucleus	biological_process	GO:0046827	3	20	5.41	0.049256469	0.697959767
regulated exocytosis	biological_process	GO:0045055	3	20	5.41	0.049256469	0.697959767
regulation of actin cytoskeleton reorganization	biological_process	GO:2000249	3	20	5.41	0.049256469	0.697959767
temperature homeostasis	biological_process	GO:0001659	3	20	5.41	0.049256469	0.697959767

Supplementary Table 9. KEGG pathway enrichment for the genes overlapping with DMRs from HCC827 vs HCC827/GR

Term	Database	ID	Input number	Background number	Rich factor	P Value	Corrected P Value
Chronic myeloid leukemia	KEGG PATHWAY	hsa05220	16	76	6.05	6.95E-05	0.019107
AMPK signaling pathway	KEGG PATHWAY	hsa04152	19	120	4.55	0.000416	0.057231
Cellular senescence	KEGG PATHWAY	hsa04218	22	160	3.95	0.000856	0.078497
FoxO signaling pathway	KEGG PATHWAY	hsa04068	19	132	4.14	0.00116	0.079767
Glioma	KEGG PATHWAY	hsa05214	13	75	4.98	0.001586	0.087246
Epstein-Barr virus infection	KEGG PATHWAY	hsa05169	24	201	3.43	0.002865	0.100981
Non-alcoholic fatty liver disease	KEGG PATHWAY	hsa04932	19	149	3.66	0.003958	0.100981
Pancreatic cancer	KEGG PATHWAY	hsa05212	12	75	4.6	0.004213	0.100981
Autophagy - animal	KEGG PATHWAY	hsa04140	17	128	3.82	0.004314	0.100981
Colorectal cancer	KEGG PATHWAY	hsa05210	13	86	4.34	0.004555	0.100981
Non-small cell lung cancer	KEGG PATHWAY	hsa05223	11	66	4.79	0.00459	0.100981
Acute myeloid leukemia	KEGG PATHWAY	hsa05221	11	66	4.79	0.00459	0.100981
Prostate cancer	KEGG PATHWAY	hsa05215	14	97	4.15	0.004782	0.100981
Endometrial cancer	KEGG PATHWAY	hsa05213	10	58	4.95	0.005466	0.100981
Hepatitis C	KEGG PATHWAY	hsa05160	19	155	3.52	0.005785	0.100981
Longevity regulating pathway	KEGG PATHWAY	hsa04211	13	89	4.2	0.005875	0.100981
Apoptosis	KEGG PATHWAY	hsa04210	17	136	3.59	0.007369	0.112647
Insulin signaling pathway	KEGG PATHWAY	hsa04910	17	137	3.57	0.007851	0.112647
Melanoma	KEGG PATHWAY	hsa05218	11	72	4.39	0.008104	0.112647
Longevity regulating pathway - multiple species	KEGG PATHWAY	hsa04213	10	62	4.64	0.008193	0.112647
Viral carcinogenesis	KEGG PATHWAY	hsa05203	22	201	3.15	0.010238	0.134073

Hepatocellular carcinoma	KEGG PATHWAY	hsa05225	19	168	3.25	0.012132	0.151644
Renal cell carcinoma	KEGG PATHWAY	hsa05211	10	69	4.17	0.015338	0.175744
Adipocytokine signaling pathway	KEGG PATHWAY	hsa04920	10	69	4.17	0.015338	0.175744
B cell receptor signaling pathway	KEGG PATHWAY	hsa04662	11	82	3.86	0.018202	0.200221
Bladder cancer	KEGG PATHWAY	hsa05219	7	41	4.91	0.019527	0.206536
Prolactin signaling pathway	KEGG PATHWAY	hsa04917	9	70	3.69	0.038114	0.374338
RIG-I-like receptor signaling pathway	KEGG PATHWAY	hsa04622	9	70	3.69	0.038114	0.374338
Neurotrophin signaling pathway	KEGG PATHWAY	hsa04722	13	119	3.14	0.041949	0.384531
Salmonella infection	KEGG PATHWAY	hsa05132	10	83	3.46	0.042026	0.384531
Human papillomavirus infection	KEGG PATHWAY	hsa05165	29	330	2.53	0.043671	0.384531
Insulin resistance	KEGG PATHWAY	hsa04931	12	108	3.19	0.044745	0.384531
Glycosaminoglycan biosynthesis - chondroitin sulfate / dermatan sulfate	KEGG PATHWAY	hsa00532	4	20	5.75	0.046729	0.389143
Human cytomegalovirus infection	KEGG PATHWAY	hsa05163	21	225	2.68	0.048112	0.389143

Supplementary Table 10. KEGG pathway enrichment for the genes overlapping with DMRs from HCC827 vs HCC827/OR

Term	Database	ID	Input number	Background number	Rich factor	P Value	Corrected P Value
Central carbon metabolism in cancer	KEGG PATHWAY	hsa05230	10	69	5.22	0.000952	0.247607
Non-alcoholic fatty liver disease	KEGG PATHWAY	hsa04932	15	149	3.63	0.001897	0.247607
AMPK signaling pathway	KEGG PATHWAY	hsa04152	12	120	3.6	0.005418	0.471391
Colorectal cancer	KEGG PATHWAY	hsa05210	9	86	3.77	0.011595	0.64365
Spliceosome	KEGG PATHWAY	hsa03040	12	135	3.2	0.01233	0.64365
EGFR tyrosine kinase inhibitor resistance	KEGG PATHWAY	hsa01521	8	79	3.65	0.019798	0.715787
MAPK signaling pathway	KEGG PATHWAY	hsa04010	20	295	2.44	0.022318	0.715787
mTOR signaling pathway	KEGG PATHWAY	hsa04150	12	153	2.83	0.027831	0.715787
Apoptosis	KEGG PATHWAY	hsa04210	11	136	2.91	0.028607	0.715787
Insulin signaling pathway	KEGG PATHWAY	hsa04910	11	137	2.89	0.029863	0.715787
Hepatitis C	KEGG PATHWAY	hsa05160	12	155	2.79	0.030167	0.715787
mRNA surveillance pathway	KEGG PATHWAY	hsa03015	8	91	3.17	0.038907	0.723297
Influenza A	KEGG PATHWAY	hsa05164	12	167	2.59	0.047213	0.723297
Type II diabetes mellitus	KEGG PATHWAY	hsa04930	5	46	3.92	0.047242	0.723297
Endocytosis	KEGG PATHWAY	hsa04144	16	244	2.36	0.047988	0.723297
Longevity regulating pathway - multiple species	KEGG PATHWAY	hsa04213	6	62	3.49	0.048349	0.723297

Supplementary Table 11. Summary of patients' clinical information (BALF, n=60).

Sample ID	Sex	Age	Pathological type	T	N	M	Clinical stage
CP001	Male	70	pneumonia				
CP002	Female	63	pneumonia				
CP003	Male	41	pneumonia				
CP004	Male	86	pneumonia				
CP005	Male	69	Bronchial polyp				
CP006	Male	75	Bronchial polyp				
CP007	Male	47	pneumonia				
CP008	Male	65	pneumonia				
CP009	Male	64	pneumonia				
CP010	Female	55	pneumonia				
BT001	Male	62	Lung squamous cell carcinomas	2	2	0	III
BT002	Female	63	Lung adenocarcinoma	1	0	0	I
BT003	Female	53	Lung adenocarcinoma	1	0	0	I
BT004	Female	37	Lung adenocarcinoma	1	0	0	I
BT005	Male	62	Lung adenocarcinoma	1	0	0	I
BT006	Male	63	Lung adenocarcinoma	1	0	0	I
BT007	Male	57	Lung adenocarcinoma	1	0	0	I
BT008	Male	45	Lung adenocarcinoma	1	0	0	I
BT009	Female	68	Lung adenocarcinoma	1	0	0	I
BT010	Male	62	Lung squamous cell carcinomas	4	3	1	IV
BT011	Female	65	Lung adenocarcinoma	1	0	0	I
BT012	Female	62	Lung adenocarcinoma	1	0	0	I
BT013	Male	63	Lung adenocarcinoma	2	0	0	I
BT014	Male	68	Lung squamous cell carcinomas	1	0	0	I
BT015	Male	68	Lung squamous cell carcinomas	2	0	0	I
BT016	Female	58	Lung adenocarcinoma	2	2	0	III
BT017	Male	57	Lung adenocarcinoma	1	0	0	I
BT018	Male	62	Lung squamous cell carcinomas	2	2	0	III
BT019	Male	82	Lung squamous cell carcinomas	4	2	1	IV
BT020	Female	55	Lung adenocarcinoma	1	0	0	I
BT021	Male	40	Lung adenocarcinoma	1	0	0	I
BT022	Female	59	Lung adenocarcinoma	1	0	0	I
BT023	Male	65	Lung adenocarcinoma	1	0	0	I
BT024	Male	76	Lung squamous cell carcinomas	1	0	0	I
BT025	Male	73	Lung squamous cell carcinomas	2	0	0	I
BT026	Male	65	Lung squamous cell carcinomas	4	3	1	IV
BT027	Female	31	Lung adenocarcinoma	1	0	0	I
GR001	Female	70	Lung adenocarcinoma	3	3	0	IV
GR002	Male	66	Lung adenocarcinoma	2	2	0	III

GR003	Female	62	Lung adenocarcinoma	4	3	1	IV
GR004	Female	63	Lung adenocarcinoma	4	0	1	IV
GR005	Female	56	Lung adenocarcinoma	4	2	0	III
GR006	Female	54	Lung adenocarcinoma	3	3	0	III
GR007	Female	45	Lung adenocarcinoma	3	3	1	IV
GR008	Female	70	Lung adenocarcinoma	2	3	1	IV
GR009	Female	37	Lung adenocarcinoma	3	2	1	IV
GR010	Female	61	Lung adenocarcinoma	2	2	1	IV
GR011	Female	57	Lung adenocarcinoma	2	3	1	IV
GR012	Female	78	Lung adenocarcinoma	3	2	1	IV
GR013	Female	67	Lung adenocarcinoma	2	0	1	IV
GR014	Female	63	Lung adenocarcinoma	4	3	1	IV
GR015	Female	66	Lung adenocarcinoma	2	2	0	III
OR001	Female	76	Lung adenocarcinoma	3	2	0	III
OR002	Female	67	Lung adenocarcinoma	3	3	1	IV
OR003	Male	68	Lung squamous cell carcinomas	3	2	0	III
OR004	Female	80	Lung adenocarcinoma	4	0	1	IV
OR005	Female	73	Lung adenocarcinoma	2	2	0	III
OR006	Male	56	Lung adenocarcinoma	3	2	0	IV
OR007	Female	60	Lung adenocarcinoma	2	1	1	IV
OR008	Male	48	Lung adenocarcinoma	4	1	0	III

Supplementary Table 12. Combination therapy with DATS and 5-Aza-CdR

DATS (μM)	5-Aza-CdR (μM)	Combination (μM)	Efficiency
6	0.05	6+0.05	0.1236
15	0.125	15+0.125	0.5145
30	0.25	30+0.25	0.6248
60	0.5	60+0.5	0.8518
150	1.25	150+1.25	0.8952
300	2.5	300+2.5	0.9651

Supplementary Table 13. CI Data for Drug Combination

Fa	CI Value	Total Dose
0.05	0.66177	1.95192
0.1	0.57828	3.54550
0.15	0.54781	5.13058
0.2	0.53483	6.77637
0.25	0.53037	8.52702
0.3	0.53107	10.4227
0.35	0.53539	12.5074
0.4	0.54265	14.8340
0.45	0.55255	17.4704
0.5	0.56510	20.5077
0.55	0.58053	24.0731
0.6	0.59931	28.3516
0.65	0.62227	33.6255
0.7	0.65076	40.3509
0.75	0.68706	49.3216
0.8	0.73532	62.0636
0.85	0.80400	81.9725
0.9	0.91431	118.620
0.95	1.14569	215.463
0.97	1.35800	329.467

Supplementary Table 14. Dose for Drug Combination

Fa	Dose DATS	Dose 5-Aza-CdR
0.05	14.2253	0.03675
0.1	19.8555	0.08743
0.15	24.4086	0.14954
0.2	28.5135	0.22400
0.25	32.4195	0.31274
0.3	36.2673	0.41860
0.35	40.1564	0.54550
0.4	44.1721	0.69887
0.45	48.3994	0.88629
0.5	52.9338	1.11861
0.55	57.8930	1.41182
0.6	63.4334	1.79043
0.65	69.7768	2.29382
0.7	77.2593	2.98923
0.75	86.4289	4.00106
0.8	98.2689	5.58616
0.85	114.795	8.36750
0.9	141.119	14.3111
0.95	196.973	34.0510
0.97	249.717	63.0940
